# Synthesis and In Vitro Antimicrobial SAR of Benzyl and Phenyl Guanidine and Aminoguanidine Hydrazone Derivatives

**DOI:** 10.3390/molecules28010005

**Published:** 2022-12-20

**Authors:** Wolfgang Dohle, Xiangdong Su, Yamni Nigam, Edward Dudley, Barry V. L. Potter

**Affiliations:** 1 Medicinal Chemistry & Drug Discovery, Department of Pharmacology, University of Oxford, Mansfield Road, Oxford OX1 3QT, UK; 2 Department of Pharmacy & Pharmacology, University of Bath, Claverton Down, Bath BA2 7AY, UK; 3 Faculty of Medicine, Health and Life Science, Swansea University, Singleton Park, Swansea SA2 8PP, UK

**Keywords:** benzyl guanidine, benzyl aminoguanidine hydrazone, guanylation, antimicrobial activity, methicillin-resistant *Staphylococcus aureus* (MRSA)

## Abstract

A series of benzyl, phenyl guanidine, and aminoguandine hydrazone derivatives was designed and in vitro antibacterial activities against two different bacterial strains (*Staphylococcus aureus* and *Escherichia coli*) were determined. Several compounds showed potent inhibitory activity against the bacterial strains evaluated, with minimal inhibitory concentration (MIC) values in the low µg/mL range. Of all guanidine derivatives, 3-[2-chloro-3-(trifluoromethyl)]-benzyloxy derivative **9m** showed the best potency with MICs of 0.5 µg/mL (*S. aureus*) and 1 µg/mL (*E. coli*), respectively. Several aminoguanidine hydrazone derivatives also showed good overall activity. Compounds **10a**, **10j**, and **10r**–**s** displayed MICs of 4 µg/mL against both *S. aureus* and *E. coli*. In the aminoguanidine hydrazone series, 3-(4-trifluoromethyl)-benzyloxy derivative **10d** showed the best potency against *S. aureus* (MIC 1 µg/mL) but was far less active against *E. coli* (MIC 16 µg/mL). Compound **9m** and the *para*-substituted derivative **9v** also showed promising results against two strains of methicillin-resistant *Staphylococcus aureus* (MRSA). These results provide new and potent structural leads for further antibiotic optimisation strategies.

## 1. Introduction

Bacterial infections with multidrug-resistant pathogens, such as methicillin-resistant *Staphylococcus aureus* (MRSA), vancomycin-resistant *Enterococci* (VRE), and multidrug-resistant *Escherichia coli,* pose an increasing threat to the global human population [1,2,3]. These drug-resistant bacteria can cause lethal infections, making the treatment of infected patients increasingly difficult. Therefore, the discovery of novel therapeutic agents that are active against drug-resistant microorganisms remains a fundamental challenge, especially for medicinal chemistry. Most antibiotics currently in clinical use target one of the metabolic pathways of DNA, RNA, protein, or cell wall synthesis [4]. Due to the emergence of pathogens with reduced susceptibility to currently available antibiotic therapies, there is an urgent need to discover new antibiotics with new targets and mechanisms of action.

In recent years, bacterial cell division has attracted considerable attention as a potential antibiotic target [5,6]. Cell division in bacteria is achieved through a highly dynamic macromolecular complex that is characterized by a time-dependent assembly of specific cell division proteins [7], formed in an orchestrated fashion by the essential tubulin homolog FtsZ (filamentous temperature-sensitive protein Z). Most bacteria depend on FtsZ as the main protein for efficient cell division [8,9]. Therefore, FtsZ has been validated as a highly promising target for antibacterial intervention [5].

**Figure 1:**
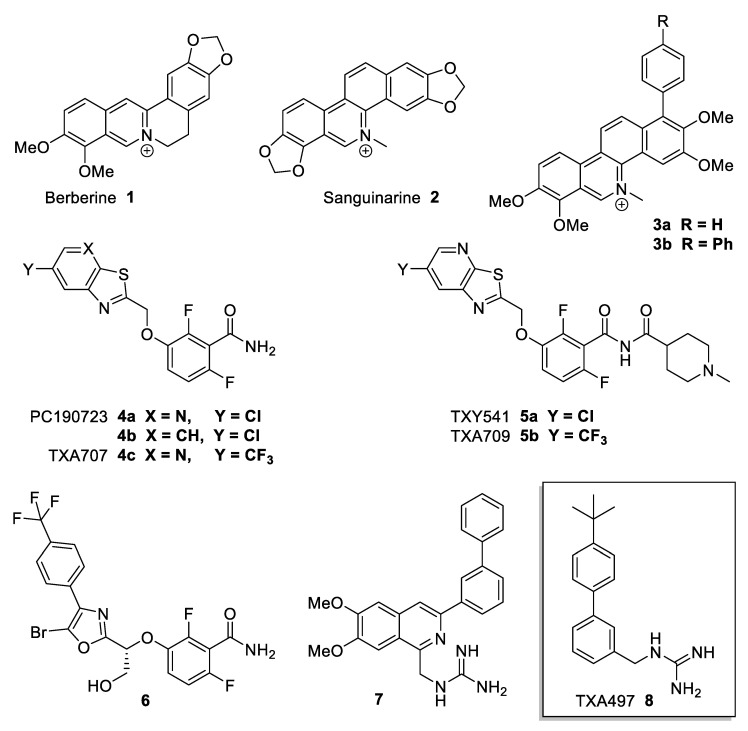
Antibiotics acting on FtsZ.

Antibacterial compounds known to target FtsZ are *eg* Berberine **1** and Sanguinarine **2** (Figure 1). Berberine **1** is a natural plant alkaloid that has been described to target *E. coli* FtsZ [10,11]. It binds to FtsZ with high affinity in a region that overlaps with the GTP binding site of FtsZ, inhibits FtsZ GTPase activity, and destabilises FtsZ protofilaments [10]. However, it showed only weak antibacterial activity against Gram-positive and Gram-negative species in recent studies [12,13]. Sanguinarine **2** is another, structurally similar, natural plant alkaloid that possesses inhibitory activity against several microorganisms, including MRSA [14]. The optimised derivatives **3a,b** showed more enhanced potency [14]. In recent years, a number of compounds have been reported that modulate the assembly/disassembly dynamics of FtsZ, some of which have shown very promising antibacterial activity against important human pathogens and are efficacious even in in vivo models of infection. Benzamide derivative PC190723 **4a** was identified as an FtsZ inhibitor with antibacterial activity against staphylococci, including multidrug-resistant *S. aureus,* with minimal inhibitory concentrations (MICs) in the range of 0.5–1.0 µg/mL [15]. PC190723 **4a** was also effective in a murine septicaemia model of staphylococcal infection and was, thus, the first FtsZ inhibitor with reported in vivo efficacy [15,16]. The closely related analogue **4b** showed an improved MIC of 0.25 µg/mL against *S. aureus,* albeit with an inferior pharmacokinetic profile [17,18]. To improve efficacy, prodrug derivative **5a** was developed but showed dechlorination and monooxygenation as a metabolic pathway, a possibility eliminated in **5b** by the introduction of CF_3_ instead of Cl [19,20,21]. Compound **6**, a recently reported advanced derivative of PC190723 **4a**, showed improved antibacterial activity with an average MIC of 0.12 µg/mL against *S. aureus* and *S. epidermidis* and high oral bioavailability [22,23].

A class of compounds regularly reported in the antibiotic context are guanidine derivatives [24]. Guanidine functionalities are commonly found in many biologically relevant molecules that constitute a versatile class of molecules with a wide range of applications. Compounds, either natural or synthetic, containing guanidine as a core unit, either in open or in cyclic form, display an array of pharmacological properties, including antimicrobial, antiviral, antiparasitic, and antifungal activities [24]. The great appeal of the guanidine moiety can be attributed to its hydrogen-bonding capability and protonatability at physiological pH in the context of interaction with biological targets. Bacterial cell envelopes are negatively charged, which may attract the guanidinium cation via electrostatic interaction and favour the binding of these compounds, leading to the disruption of cell membranes and cell walls. Therefore, guanidine derivatives have been exploited as privileged structural motifs in designing novel drugs for the treatment of various infectious and non-infectious diseases. Over many years, a large variety of synthetic small molecules with one or several guanidine units has emerged [24]. There are also synthetic polymeric guanidine derivatives that display very potent antibiotic activities against MRSA in skin infections and against the growth of *Aspergillus parasiticus* [25,26].

Recent examples of synthetic, small-molecule-type, investigational antibiotics with guanidine motifs are compounds **7** and TXA497 **8** (Figure 1) [27,28,29,30]. Biphenyl derivative **8** especially, despite its structural simplicity, displays quite a few remarkable antimicrobial properties [29,30]. First, **8** shows low MICs (1 µg/mL) against several variations of *S. aureus* including MRSA. Furthermore, **8** also displays a very low MBC value (minimum bactericidal concentration) of 1 µg/mL, leading to a ratio of MBC/MIC = 1 which essentially means that 99.9% of all bacterial cells are killed at the same concentration of minimum inhibition (MIC). The same MBC/MIC ratio for **8** was observed against *E. coli*, but, at 16 µg/mL, the compound concentrations were much higher [30]. However, more remarkably, it was found that **8** exhibits only a minimal potential for inducing resistance in *S. aureus* [30]. Initially, **8** was proposed to act on FtsZ dynamics and it was shown to target the GTP binding site of recombinant FtsZ in vitro [30]. However, in bacterial cells, **8** targets the bacterial cell membrane in addition to FtsZ, with some cells predominantly showing the effects of FtsZ inhibition and others predominantly showing the effects of cell membrane disruption [31]. The guanidino/amidino functionality is a key contributor to the antibacterial activity of this class of compounds.

**Figure 2:**
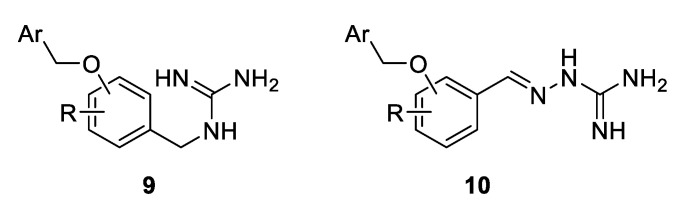
Design of benzyl guanidine and aminoguanidine hydrazone derivatives with a variety of benzyloxy groups.

The structural simplicity of **8** was highly attractive from a medicinal chemistry standpoint. As a part of a program to design a library of novel antimicrobial compounds, only a small set of benzyl guanidine **9** and aminoguanidine hydrazone derivative **10** with a variety of benzyloxy groups was initially envisaged (Figure 2). However, the compound series was later expanded, and new broadly-related, but also diverse, guanidine derivatives were additionally synthesised and their antimicrobial activities against *S. aureus* and *E. coli* were evaluated [32]. The most potent inhibitors were then tested against the drug-resistant strains MRSA 3 and MRSA 15.

## 2. Results and Discussion

### 2.1. Chemistry

**Scheme 1:**
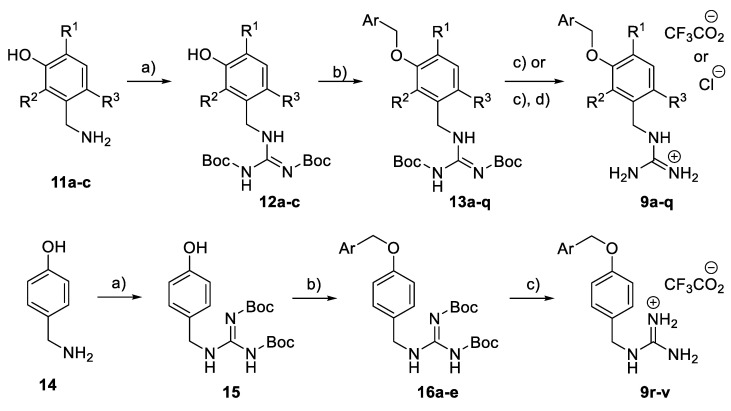
Synthesis of benzyl guanidine derivatives **9a**–**v**. *Reagents and conditions:* (a) BocN=C(SMe)-NHBoc, Et_3_N, DMF, rt; (b) Benzyl halide, K_2_CO_3_, actone; (c) TFA, CH_2_Cl_2_, rt; (d) HCl (0.5M in MeOH), rt.

The *meta*-substituted benzyl guanidine compounds **9a**–**q** were constructed from the corresponding 3-aminomethylphenol derivatives **11a**–**c** via a guanylation reaction using Boc-protected *S*-methylisothiourea [33], followed by the benzylation of the phenol group under basic conditions to give **13a**–**q**. Finally, treatment with trifluoroacetic acid in dichloromethane led to **9a**–**q,** obtained as their guanidinium trifluoroacetate or chloride salts (Scheme 1). Benzyl guanidine derivatives **9r**–**v** were prepared under the same conditions using 4-aminomethylphenol **14** as the starting material.

**Scheme 2:**
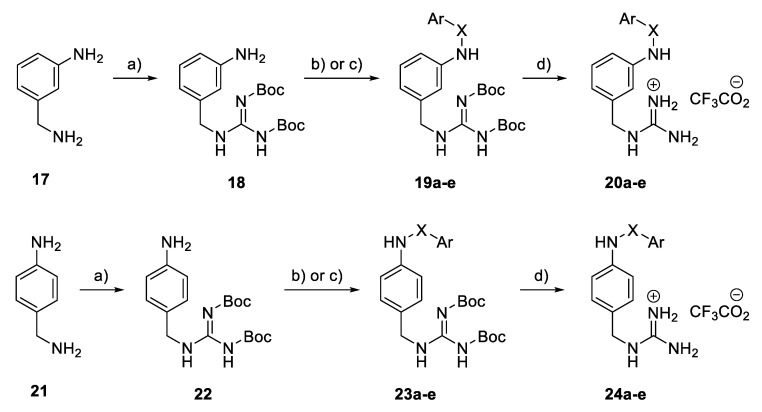
Synthesis of benzyl guanidine derivatives **20a–e** and **24a–e**. *Reagents and conditions:* (a) BocN=C(SMe) NHBoc, Et_3_N, DMF, rt; (b) ArSO_2_Cl, pyridine, CH_2_Cl_2_, 0 °C; (c) Benzoyl chloride, K_2_CO_3_, actone, 80 °C; (d) TFA, CH_2_Cl_2_, rt.

In a benzylguanidine-based structural subset, the *meta*- and *para*-substituted compounds **20a**–**e** and **24a**–**e** (Scheme 2) were constructed from 3- and 4-aminomethylaniline **17** and **21** via a guanylation reaction using Boc-protected *S*-methylisothiourea, followed by the treatment of the resulting **18** and **22,** respectively, with the corresponding arylsulfonyl chloride or benzoyl chloride in the presence of a base to achieve Boc-protected derivatives **19a**–**e** and **23a**–**e**. Treatment with trifluoroacetic acid in dichloromethane led to the removal of the Boc groups, and the final compounds **20a**–**e** and **24a**–**e** were obtained as their guanidinium trifluoroacetate salts.

**Scheme 3:**
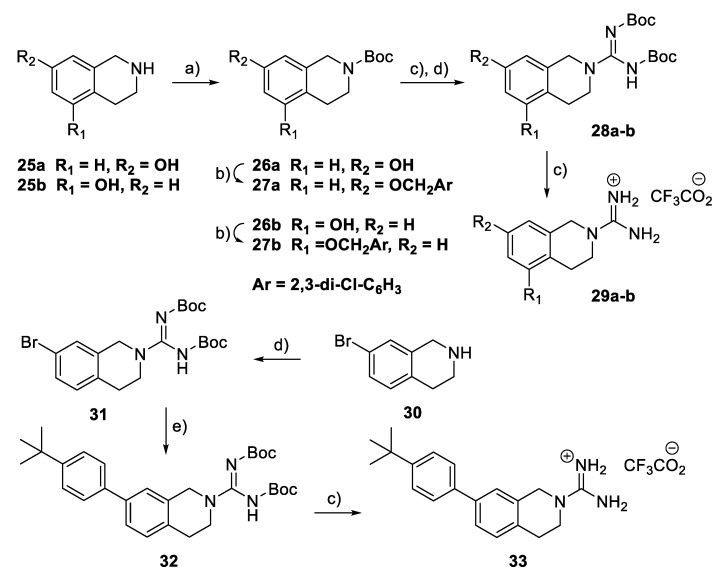
Synthesis of tetrahydroisoquinolinylguanidine derivatives **29a–b** and **33**. *Reagents and conditions:* (a) Boc_2_O, THF-H_2_O, Et_3_N, rt; (b) 2,3-Dichlorobenzyl bromide, K_2_CO_3_, acetone, rt; (c) TFA, CH_2_Cl_2_, rt; (d) BocN=C(SMe)-NHBoc, Et_3_N, DMF, rt; (e) 4-*t*-Butylphenylboronic acid, Pd(Ph_3_)_4_, K_2_CO_3_, dioxane, 100 °C.

To explore the potential effects of conformational restriction of the guanidine moiety, the tetrahydroisoquinoline-based compounds **29a–b** and **33** were prepared via the route shown in Scheme 3. First, compounds **29a–b** were prepared from the corresponding hydroxy-substituted 1,2,3,4-tetrahydroisoquinolines **25a–b** by *N*-Boc protection, benzylation, guanylation, and Boc deprotection. Similarly, guanylation of 7-bromo-1,2,3,4-tetrahydroisoquinoline **30** gave the corresponding 2-carboximidamide derivative **31** that was converted to **33** through a route involving a palladium-catalysed Suzuki coupling [30], followed by the removal of the Boc groups with TFA.

**Scheme 4:**
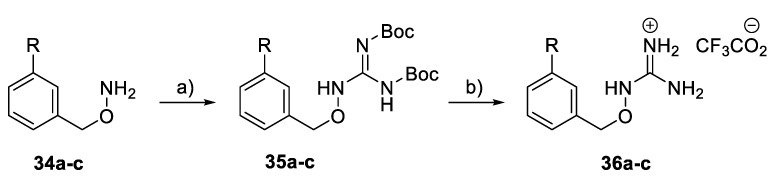
Synthesis of benzyloxy guanidine derivatives **36a–c**. *Reagents and conditions:* (a) BocN=C(SMe)-NHBoc, Et_3_N, DMF, rt; (b) TFA, CH_2_Cl_2_, rt.

A subset of benzoyloxyguanidine compounds **36a–c** (Scheme 4) was synthesised from the corresponding amine via a guanylation reaction, followed by the removal of the Boc protection groups in the presence of TFA.

**Scheme 5:**
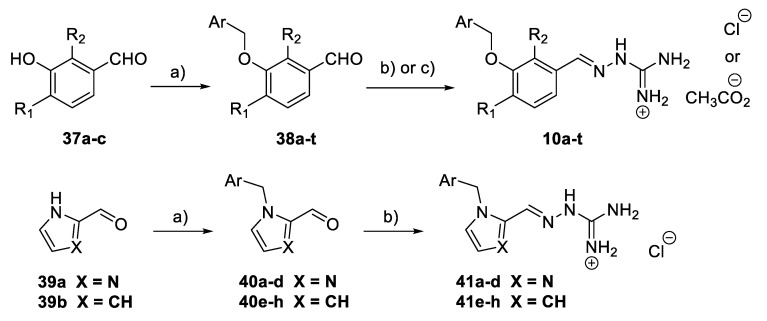
Synthesis of aminoguanidino hydrazone derivatives **10a–t** and **41a–h**. *Reagents and conditions:* (a) Benzyl halide, K_2_CO_3_, DMF, rt; (b) *N*-aminoguanidine bicarbonate, HCl (0.5 M in MeOH), 80 °C; (c) *N*-aminoguanidine bicarbonate, AcOH, MeOH, 80 °C.

A subset of aminoguanidino hydrazone derivatives **10a–t** (Scheme 5) was prepared in two steps from the corresponding 3-hydroxybenzaldehyde derivatives **37a–c**, by benzylation of the hydroxyl group and condensation of the corresponding aldehydes **38a–t** with *N*-aminoguanidine bicarbonate [34]. Most of the target compounds were obtained as their chloride salts and a few as acetates. Imidazole aminoguanidine (**41a–d**) and pyrrole aminoguanidine derivatives (**41e–h**) were synthesised as chloride salts in the same way using HCl (0.5M in MeOH) at 80 °C.

**Scheme 6:**
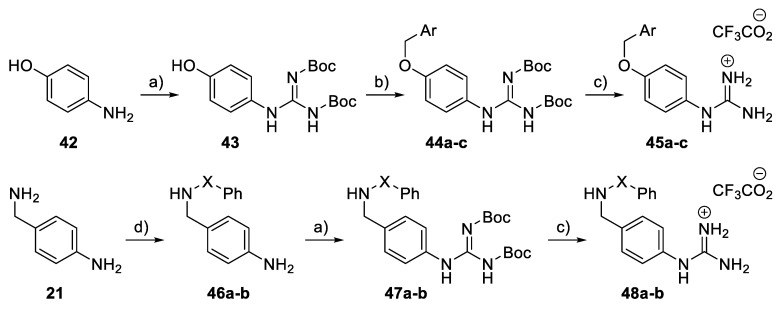
Synthesis of phenyl guanidine derivatives **45a–c** and **48a–b**. *Reagents and conditions:* (a) BocN=C(SMe)-NHBoc, HgCl_2_, Et_3_N, DMF, rt; (b) Benzyl halide, K_2_CO_3_, acetone, rt; (c) TFA, CH_2_Cl_2_, RT; (d) Benzoyl chloride or benzenesulphonyl chloride, Et_3_N, DMF, 0 °C.

For phenylguanidino derivatives (Scheme 6), a guanylation reaction of *para*-aminophenol **42** generated the intermediate **43**, which was subsequently subjected to benzylation to afford the *N,N*′-di-Boc protected guanidine derivatives **44a–c**. Successive treatment with trifluoroacetic acid in dichloromethane gave **45a–c**. Phenyl guanidine derivatives **48a–b** were achieved in four steps. In the first step, 4-aminobenzylamine **21** was treated with either benzoyl chloride or benzenesulphonyl chloride and triethylamine in DMF to give **46a–b**. Guanylation with Boc-protected *S*-methylisothiourea in the presence of mercury (II) chloride [35] then achieved **47a–b**. Subsequent treatment with TFA removed the Boc groups to give **48a–b**.

**Scheme 7:**
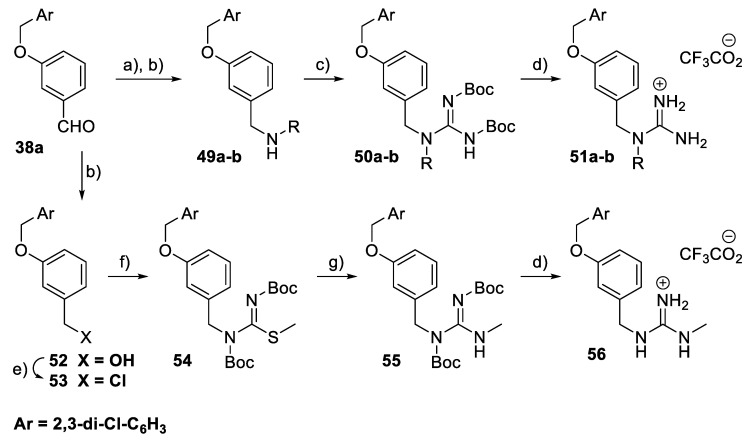
Synthesis of benzyl guanidine derivatives **51a–b** and **56**. *Reagents and conditions:* (a) R-NH_2_, MeOH, rt; (b) NaBH_4_, MeOH, 0 °C; (c) BocN=C(SMe)-NHBoc, Et_3_N, DMF, rt; (d) TFA, CH_2_Cl_2_, rt; (e) CH_3_SO_2_Cl, Et_3_N, CH_2_Cl_2_, rt. (f) BocN=C(SMe)-NHBoc, KOH, CH_2_Cl_2_, H_2_O, rt; (g) MeNH_2_, HgCl_2_, Et_3_N, DMF, rt.

For a subset of benzyl guanidine derivatives **51a–b** and **56** (Scheme 7), the reductive amination reaction of the aldehyde **38a** (Ar = 2,3-dichlorophenyl) generated the amino intermediates **49a–b**, which underwent a guanylation reaction to form the *N,N*′-di-Boc protected guanidine derivatives **50a–b**. Deprotection of the Boc groups generated the guanidinium trifluoroacetate salts **51a–b**. The intermediate **53** was obtained through the reduction of the aldehyde **38a**, followed by halogenation of the resulting benzyl alcohol **52**. Treatment of **53** with *S*-methyl-*N,N*′-bis(*tert*-butoxycarbonyl)isothiourea under basic conditions gave **54**. Nucleophilic substitution of **54** with methylamine afforded the *N,N*′-di-Boc protected guanidine **55**, which was then hydrolysed in TFA to give the final compound **56**.

### 2.2. Biology

#### 2.2.1. In Vitro Antimicrobial Activity against *S. aureus* and *E. coli*

Table 1 reveals that a significant proportion of the synthesised benzyl guanidine derivatives is more potent against *S. aureus* than against *E. coli*. For **9a–m** (R^1^ = R^2^ = R^3^ = H), only **9d**, **9h**, and **9k** are more potent against *E. coli* than against *S. aureus*, with **9h** showing the best activity (MIC = 4 µg/mL) of them. However, the biggest difference in potency against the two strains was found for **9d** with MICs of >256 µg/mL and 8 µg/mL, respectively. The most potent compound in this subset was the 2-Cl-3-CF_3_ derivative **9m** with MICs of 0.5 µg/mL and 1 µg/mL, respectively, but 2,3-dichloro derivative **9g** showed very similar potency with MICs of 1 µg/mL against both microbial strains. For **9n–o** (R^1^ = MeO, R^2^ = R^3^ = H), reduced potency was found. Comparison of **9n** with **9c** showed a significantly reduced potency for an H to MeO substitution. A MIC of 128 µg/mL for **9n** and MICs of 16 µg/mL and 32 µg/mL for **9c** were found. 4-Chloro derivatives **9o** and **9b** showed a similar pattern against *E. coli* but appeared to be equipotent against *S. aureus* with MICs of 8 µg/mL for both compounds. Derivatives **9p–q** (R^1^ = H, R^2^ = R^3^ = F) proved very potent against *S. aureus* with MICs of 0.5 µg/mL and 1 µg/mL, respectively. However, in contrast to **9g** and **9m**, which were two compounds very active against both strains, **9p–q** were significantly less potent against *E. coli*. A few *para*-substituted benzyl guanidine derivatives **9r–v** were also evaluated. The monochlorobenzyl derivatives **9s–t** proved both moderately active and did not show any difference in potency between the two microbial strains. The dichlorobenzyl derivatives **9u–v**, on the other hand, proved significantly more potent against *S. aureus* than against *E. coli*, with **9v** showing the best overall antimicrobial activities with MICs of 0.5 µg/mL and 4 µg/mL, respectively. Substitution of one hydrogen at the *N* atom of the guanidine unit in **9g**, where the benzyl unit is attached with a methyl or a methoxyethyl group, led to a significant decrease in antimicrobial potency, from a MIC of 1 µg/mL for **9g** to MICs of 32 µg/mL for **51a–b**. A slightly better, but still very weak activity, against S. aureus was found for **56**, a derivative where the methyl group was introduced at the terminal *N* atom (MIC of 16 µg/mL).

Benzyl guanidines with aminosulfonylaryl or aminobenzoyl motifs as substituents either in the *meta*- (**20a–e**) or *para*-position (**24a–e**) proved uniquely inactive for both sets of compounds (all MICs > 32 µg/mL, Table 2).

The antimicrobial activities of tetrahydroisoquinoline guanidine derivatives **29a–b** and **33** are summarised in Table 3. A comparison of **29a** and **29b** reveals that substitution in the 5-position seems more favourable than in the 7-positon. However, even 5-substituted derivative **29b** proved only moderately active with MICs of 8 µg/mL for both *S. aureus* and *E. coli*. Replacing the *O*-benzyl linkage in the 7-position with a directly attached aromatic ring system seems to further reduce antimicrobial activity. 4-*tert*-Butylphenyl derivative **33** showed only weak potency with MICs of 64 µg/mL and >128 µg/mL, respectively.

The antimicrobial activities of the three benzyloxy guanidine derivatives against *S. aureus* and *E. coli* are summarised in Table 4. All compounds of this class that we tested so far displayed no significant potency.

The MIC values against *S. aureus* and *E. coli* of aminoguanidine hydrazone derivatives **10a–t** and **41a–h** are summarised in Table 5. Compounds **10a–f** (R^1^ = R^2^ = H) showed overall moderate to good antimicrobial activities with the majority of MICs between 4 µg/mL and 16 µg/mL. Compound **10d** was significantly active against *S. aureus* (MIC of 1 µg/mL) but less against *E. coli* (MIC of 16 µg/mL) and was the most potent compound against *S. aureus* of all aminoguanidine hydrazone derivatives. Methoxy-substituted derivative **10g** (R^1^ = MeO, R^2^ = H) appeared to be less potent (MICs of 16 µg/mL and 8 µg/mL) when directly compared with **10a** (both MICs of 4 µg/mL). Chloro-substituted compounds **10h–t** (R^1^ = H, R^2^ = Cl) showed MICs between 4 µg/mL and 32 µg/mL. The most potent derivatives here were **10j** and **10r–s**, all of which are mono-substituted in the benzyloxy motif (4-Cl, 3-CF_3_, 4-CF_3_). All three compounds showed MICs of 4 µg/mL against both *S. aureus* and *E. coli*. Heterocyclic derivatives, including benzimidazole aminoguanidine hydrazones **41a–d** as well as pyrrole aminoguanidine hydrazones **41e–h,** displayed only moderate antimicrobial activities, with **41d** showing the best potency against *S. aureus* (MIC of 8 µg/mL).

Table 6 shows a summary of the MIC values for *para*-substituted phenyl guanidine derivatives **45a–c** and **48a–b** against *S. aureus* and *E. coli*. All derivatives showed only moderate antimicrobial potency with **45a** displaying the best activity against *S. aureus* (MIC of 8 µg/mL).

#### 2.2.2. Antimicrobial Activity against MRSAs

**Figure 3:**
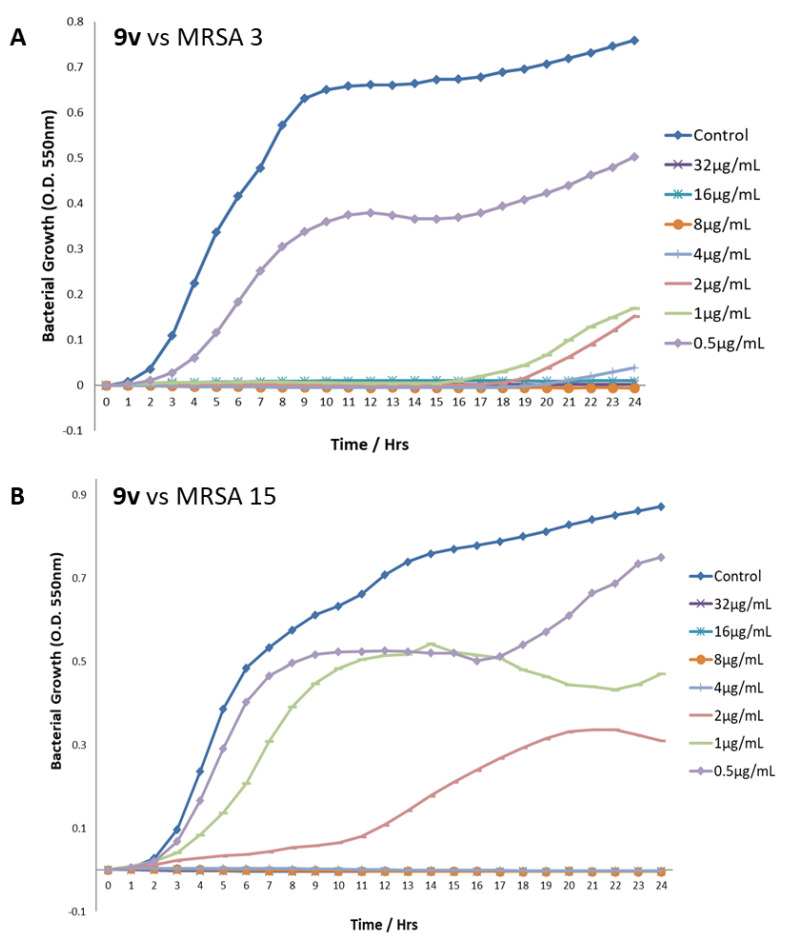
Growth profiles of MRSA strains 3 and 15 after treatment with different concentrations of compound **9v**. (**A**) **9v** vs. MRSA 3. (**B**) **9v** vs. MRSA 15.

The benzyl guanidine derivatives **9m** and **9v** were also tested against MRSA 3 and MRSA 15. Bacterial growth was recorded against time at various concentrations of **9m** and **9v**. The example data for the growth of the MRSAs when treated with differing doses of **9v** are shown in Figure 3. The minimum inhibitory concentration (MIC) and the survival index (SI) were established for each experiment (Table 7). In order to determine the SI, the growth of the treated bacteria was compared to the growth (measured as an increase in optical density over time) of the control, untreated bacteria, and the MIC was determined as the concentration that allowed an SI reduction of greater than 50%. As can be seen in Figure 3, as representative plots, although the total optical density change during the control growth varies slightly between the experiments, the growth curve progress and, hence, the overall growth profile of the control bacteria are very consistent, and each experiment’s individual control bacterial growth curve allows for minor variations in growth between the different compound treatments. The majority of the doses above the determined MIC values demonstrate a complete absence of growth of the bacteria over the 24 h of the experiments, with the concentrations around and below the MIC (where present) clearly showing a dose dependent, if only partial, recovery of the usual growth profile of the bacteria. The reported SI values at the MIC concentrations of each compound range between 1.76 and 12.76%, indicating a vastly reduced growth behaviour of the bacteria in all treatments.

## 3. Materials and Methods

### 3.1. Chemistry

**Methods and Materials:** All chemicals and anhydrous solvents were purchased from either Sigma-Aldrich (now Merck: Gillingham, UK) or Alfa Aesar (Heysham, UK). All organic solvents of AR grade were supplied by Fisher Scientific (Loughborough, UK). Melting points were determined using a Stanford Research Systems Optimelt MPA100 (Stanford Research Systems, Sunnyvale, CA, USA) and were uncorrected. Thin-layer chromatography (TLC) was performed on pre-coated aluminium plates (Merck, silica gel 60 F_254_). The products were visualised either by UV irradiation at 254 nm or by staining with 5% *w*/*v* phosphomolybdic acid in ethanol, followed by heating. Flash column chromatography was performed on pre-packed silica gel columns (RediSep Rf) and gradient elution (solvents indicated in text) on the Combiflash Rf system (Teledyne Isco). ^1^H NMR spectra were recorded with a Bruker 400 or 500 MHz spectrometer. The chemical shifts were reported in parts per million (ppm), either relative to the corresponding solvent residual peaks or tetramethylsilane (TMS) as an internal standard. High-resolution mass spectra (HRMS) were recorded on a Bruker MicroTOF with ESI. All compounds were ≥95% pure by ^1^H NMR spectroscopy.

**General Procedure: Guanylation of substituted 3-(aminomethyl)phenols (11a–c):** In a solution of the substituted 3-(aminomethyl)phenol hydrochloride **11a–c** (6.0 mmol) in DMF (25 mL), *S*-methyl-*N,N*′-bis(*tert*-butoxycarbonyl) isothiourea (1.3 g, 4.5 mmol) was added, followed by Et_3_N (3.0 mL). The mixture was stirred at r.t. overnight and partitioned between EtOAc (100 mL) and citric acid (50 mL, 5% in water). The organic layer was washed with brine, dried (MgSO_4_), and concentrated in vacuo to give an off-white solid. Purification by flash column chromatography (petrol ether to petrol ether/EtOAc 11:9) afforded **12a**–**c**.

***tert*-Butyl *N*-[{[(tert-butoxy)carbonyl]imino}({[(3-hydroxyphenyl)methyl]amino}) methyl]carbamate (12a):** A white solid was obtained (75% yield), m.p. 159–161 °C. ^1^H NMR (400 MHz, CDCl_3_): *δ* 1.48 (9H, s, *t*-Bu), 1.50 (9H, s, *t*-Bu), 4.55 (2H, d, *J* = 5.2 Hz, CH_2_), 6.69–6.80 (3H, m, 3 × ArH), 7.15 (1H, t, *J* = 7.2 Hz, ArH), 8.69 (1H, t, *J* = 5.2 Hz, NH), and 11.6 (1H, s, NH). ^13^C NMR (100 MHz, CDCl_3_): *δ* 28.2, 28.4, 44.7, 79.7, 83.5, 114.6, 114.7, 119.6, 129.9, 139.1, 153.3, 156.3, 156.4, and 163.5. HRMS (ESI): Calcd. for C_18_H_28_N_3_O_5_ (M + H)^+^ 366.2029 and found 366.2008.

***tert*-Butyl *N*-{[(tert-butoxy)carbonyl]imino}({[(3-hydroxy-4-methoxyphenyl) methyl]amino})methyl]carbamate** (**12b**): A white solid was obtained (69% yield), m.p. 127–129 °C. ^1^H NMR (400 MHz, CDCl_3_): *δ* 1.48 (9H, s,* t*-Bu), 1.50 (9H, s, *t*-Bu), 3.87 (3H, s, OCH_3_), 4.51 (2H, d, *J* = 5.2 Hz, CH_2_), 6.76–6.79 (2H, m, 2 × ArH), 6.88 (1H, d, *J* = 1.0 Hz, ArH), 8.49 (1H, br.s, NH), and 11.5 (1H, s, NH). HRMS (ESI): Calcd. for C_19_H_30_N_3_O_6_ (M + H)^+^ 396.2135 and found 396.2148.

***tert*-Butyl *N*-[{[(*tert*-butoxy)carbonyl]imino}({[(2,6-difluoro-3-hydroxyphenyl)methyl]amino})methyl]carbamate** (**12c**): A white solid was obtained (59% yield), m.p. 132–134 °C. ^1^H NMR (400 MHz, CDCl_3_) *δ* 1.47 (9H, s, *t*-Bu), 1.52 (9H, s, *t*-Bu), 4.77 (2H, br.s, CH_2_), 6.79 (1H, m, ArH), 6.99 (1H, m, ArH), 8.70 (1H, br.s, NH), and 11.6 (1H, s, NH). HRMS (ESI): Calcd. for C_18_H_26_F_2_N_3_O_5_ (M + H)^+^ 402.1840 and found 402.1857.

**General Procedure: Benzylation of substituted 3-(*N,N*′-di-Boc-guanydinomethyl)phenols (12a–c):** In a solution of the substituted 3-(*N,N*′-di-Boc-guanydinomethyl)phenol (**12a–c**) (0.56 mmol) in acetone (8 mL), the substituted benzyl bromide (0.56 mmol) was added, followed by K_2_CO_3_ (96 mg). The mixture was stirred at r.t. overnight and partitioned between EtOAc (50 mL) and water (50 mL). The organic layer was washed with brine, dried (MgSO_4_), and concentrated in vacuo to give an off-white solid. Purification by flash column chromatography (petrol ether to petrol ether/EtOAc 3:1) afforded **13a–q**.

***tert*-Butyl *N*-[({[3-(benzyloxy)phenyl]methyl}amino)({[(*tert*-butoxy) carbonyl] amino})methylidene]carbamate (13a):** A white solid was obtained (85% yield), m.p. 90–91 °C. ^1^H NMR (400 MHz, CDCl_3_): *δ* 1.48 (9H, s, *t*-Bu), 1.51 (9H, s, *t*-Bu), 4.60 (2H, d, *J* = 5.2 Hz, CH_2_), 5.05 (2H, s, CH_2_), 6.88–6.92 (2H, m, 2 × ArH), 6.96 (1H, t, *J* = 2.0 Hz, ArH), 7.25 (1H, t, *J* = 8.0 Hz, ArH), 7.30–7.45 (5H, m, 5 × ArH), 8.59 (1H, br.s, NH), and 11.52 (1H, s, NH). HRMS (ESI): Calcd. for C_25_H_33_N_3_NaO_5_^+^ (M + Na)^+^ 478.2318 and found 478.2312.

***tert*-Butyl *N*-[{[(*tert*-butoxy)carbonyl]amino}[({3-[(4-chlorophenyl)methoxy]phenyl}methyl)amino]methylidene]carbamate (13b):** A white solid was obtained (64% yield), m.p. 113–115 °C. ^1^H NMR (400 MHz, CDCl_3_): *δ* 1.48 (9H, s, *t*-Bu), 1.50 (9H, s, *t*-Bu), 4.59 (2H, d, *J* = 5.3 Hz, CH_2_), 5.01 (2H, s, CH_2_), 6.85–6.95 (3H, m, 3 × ArH), 7.22–7.35 (5H, m, 5 × ArH), 8.58 (1H, br.s, NH), and 11.53 (1H, s, NH). HRMS (ESI): Calcd. for C_25_H_33_ClN_3_O_5_ (M + H)^+^ 490.2109 and found 490.2122.

***tert*-Butyl *N*-[{[(*tert*-butoxy)carbonyl]amino}[({3-[(3-chlorophenyl)methoxy]phenyl}methyl)amino]methylidene]carbamate (13c):** A clear oil was obtained (85 % yield). ^1^H NMR (400 MHz, CDCl_3_): *δ* 1.47 (9H, s, *t*-Bu), 1.50 (9H, s, *t*-Bu), 4.59 (2H, d, *J* = 5.5 Hz, CH_2_), 5.01 (2H, s, CH_2_), 6.85–6.95 (3H, m, 3 × ArH), 7.22–7.35 (4H, m, 4 × ArH), 7.43 (1H, s, ArH), 8.60 (1H, br.s, NH), and 11.52 (1H, s, NH). HRMS (ESI): Calcd. for C_25_H_33_ClN_3_O_5_ (M + H)^+^ 490.2109 and found 490.2135.

***tert*-Butyl *N*-[{[(*tert*-butoxy)carbonyl]amino}[({3-[(2,4-dichlorophenyl)methoxy]-phenyl}methyl)amino]methylidene]carbamate (13d):** A white solid was obtained (55% yield), m.p. 111–112 °C. ^1^H NMR (400 MHz, CDCl_3_): *δ* 1.48 (9H, s, *t*-Bu), 1.51 (9H, s, *t*-Bu), 4.60 (2H, d, *J* = 5.1 Hz, CH_2_), 5.11 (2H, s, CH_2_), 6.88–6.94 (3H, m, 3 × ArH), 7.24–7.28 (2H, m, 2 × ArH), 7.41 (1H, d, *J* = 1.9 Hz, ArH), 7.49 (1H, d, *J* = 8.2 Hz, ArH), 8.59 (1H, br.s, NH), and 11.54 (1H, s, NH). HRMS (ESI): Calcd. for C_25_H_31_Cl_2_N_3_NaO_5_ (M + Na)^+^ 546.1538 and found 546.1507.

***tert*-Butyl *N*-[{[(*tert*-butoxy)carbonyl]amino}[({3-[(3,4-dichlorophenyl) methoxy] phenyl}methyl)amino]methylidene]carbamate (13e):** A white solid was obtained (52% yield), m.p. 131–132 °C. ^1^H NMR (400 MHz, CDCl_3_): *δ* 1.48 (9H, s, *t*-Bu), 1.51 (9H, s, *t*-Bu), 4.60 (2H, d, *J* = 5.2 Hz, CH_2_), 5.00 (2H, s, CH_2_), 6.75–6.90 (3H, m, 3 × ArH), 7.22–7.30 (2H, m, 2 × ArH), 7.45 (1H, d, *J* = 8.2 Hz, ArH), 7.53 (1H, d, *J* = 2.0 Hz, ArH), 8.59 (1H, br.s, NH), and 11.53 (1H, s, NH). HRMS (ESI): Calcd. for C_25_H_31_Cl_2_N_3_NaO_5_ (M + Na)^+^ 546.1538 and found 546.1525.

***tert*-Butyl *N*-[{[(*tert*-Butoxy)carbonyl]amino}[({3-[(2,5-dichlorophenyl) methoxy] phenyl}methyl)amino]methylidene]carbamate (13f):** A white solid was obtained (69% yield), m.p. 127–129 °C. ^1^H NMR (400 MHz, CDCl_3_): *δ* 1.48 (9H, s, *t*-Bu), 1.51 (9H, s, *t*-Bu), 4.61 (2H, d, *J* = 5.1 Hz, CH_2_), 5.10 (2H, s, CH_2_), 6.89–6.95 (3H, m, 3 × ArH), 7.22–7.33 (3H, m, 3 × ArH), 7.58 (1H, d, *J* = 2.5 Hz, ArH), 8.60 (1H, br.s, NH), and 11.53 (1H, s, NH). HRMS (ESI): Calcd. for C_25_H_31_Cl_2_N_3_NaO_5_ (M + Na)^+^ 546.1538 and found 546.1506.

***tert*-Butyl *N*-[{[(*tert*-butoxy)carbonyl]amino}[({3-[(2,3-dichlorophenyl) methoxy] phenyl}methyl)amino]methylidene]carbamate (13g):** A white solid was obtained (65% yield), m.p. 98–100 °C. ^1^H NMR (400 MHz, CDCl_3_): *δ* 1.48 (9H, s, *t*-Bu), 1.51 (9H, s, *t*-Bu), 4.59 (2H, d, *J* = 5.2 Hz, CH_2_), 5.16 (2H, s, CH_2_), 6.87–6.94 (3H, m, 3 × ArH), 7.26 (2H, m, 2 × ArH), 7.46 (2H, m, 2 × ArH), 8.59 (1H, br.s, NH), and 11.54 (1H, s, NH). ^13^C NMR (100 MHz, CDCl_3_): *δ* 28.2, 28.4, 45.0, 67.6, 79.6, 83.4, 114.0, 114.6, 120.9, 126.8, 127.6, 129.8, 130.1, 130.7, 133.2, 137.2, 139.2, 153.3, 156.3, 158.7, and 163.7. HRMS (ESI): Calcd. for C_25_H_31_Cl_2_N_3_NaO_5_ (M + Na)^+^ 546.1538 and found 546.1529.

***tert*-Butyl *N*-[{[(*tert*-butoxy)carbonyl]amino}({[(3-{[4-(trifluoromethyl)phenyl] methoxy}phenyl)methyl]amino})methylidene]carbamate (13h):** A white solid was obtained (79% yield), m.p. 108–110 °C. ^1^H NMR (400 MHz, CDCl_3_) δ 1.48 (9H, s, *t*-Bu), 1.51 (9H, s, *t*-Bu), 4.59 (2H, d, *J* = 5.2 Hz, CH_2_), 5.12 (2H, s, CH_2_), 6.88–6.95 (3H, m, 3 × ArH), 7.25 (1H, t, *J* = 8.0 Hz, ArH), 7.55 (2H, d, *J* = 8.2 Hz, 2 × ArH), 7.65 (2H, d, *J* = 8.2 Hz, 2 × ArH), 8.59 (1H, br.s, NH), and 11.53 (1H, s, NH). HRMS (ESI): Calcd. for C_26_H_33_F_3_N_3_O_5_ (M + H)^+^ 524.2372 and found 524.2354.

***tert*-Butyl *N*-[{[(tert-butoxy)carbonyl]amino}({[(3-{[3-(trifluoromethyl)phenyl] methoxy}phenyl)methyl]amino})methylidene]carbamate (13i):** A clear oil was obtained (71% yield). ^1^H NMR (400 MHz, CDCl_3_): δ 1.48 (9H, s, *t*-Bu), 1.51 (9H, s, *t*-Bu), 4.60 (2H, d, *J* = 5.2 Hz, CH_2_), 5.12 (2H, s, CH_2_), 6.87–6.97 (3H, m, 3 × ArH), 7.27 (1H, t, *J* = 7.8 Hz, ArH), 7.50 (1H, t, *J* = 7.6 Hz, ArH), 7.58 (1H, d, *J* = 8.5 Hz, ArH), 7.62 (1H, d, *J* = 8.5 Hz, ArH), 7.70 (1H, s, ArH), 8.59 (1H, br.s, NH), and 11.54 (1H, s, NH). HRMS (ESI): Calcd. for C_26_H_32_F_3_N_3_NaO_5_ (M + Na)^+^ 546.2192 and found 546.2206.

***tert*-Butyl *N*-[{[(*tert*-butoxy)carbonyl]amino}[({3-[(4-bromophenyl)methoxy] phenyl}methyl)amino]methylidene]carbamate (13j):** A white solid was obtained (59% yield), m.p. 121–122 °C. ^1^H NMR (400 MHz, CDCl_3_): *δ* 1.48 (9H, s, *t*-Bu), 1.50 (9H, s, *t*-Bu), 4.59 (2H, d, *J* = 5.3 Hz, CH_2_), 5.00 (2H, s, CH_2_), 6.84–6.94 (3H, m, 3 × ArH), 7.25 (1H, t, *J* = 7.9 Hz, ArH), 7.29–7.33 (2H, m, 2 × ArH), 7.50 (2H, d, *J* = 8.5 Hz, 2 × ArH), 8.58 (1H, br.s, NH), and 11.52 (1H, s, NH). HRMS (ESI): Calcd. for C_25_H_32_BrN_3_NaO_5_ (M + Na)^+^ 556.1423 and found 556.1405.

***tert*-Butyl *N*-[{[(*tert*-butoxy)carbonyl]amino}[({3-[(4-fluorophenyl)methoxy] phenyl}methyl)amino]methylidene]carbamate (13k):** A white solid was obtained (57% yield), m.p. 98–99 °C. ^1^H NMR (400 MHz, CDCl_3_): *δ* 1.49 (9H, s, *t*-Bu), 1.51 (9H, s, *t*-Bu), 4.59 (2H, d, *J* = 5.3 Hz, CH_2_), 5.01 (2H, s, CH_2_), 6.86–6.94 (3H, m, 3 × ArH), 7.04–7.08 (2H, m, 2 × ArH), 7.25 (1H, t, *J* = 8.2 Hz, ArH), 7.38–7.42 (2H, m, 2 × ArH), 8.58 (1H, br.s, NH), and 11.53 (1H, s, NH). HRMS (ESI): Calcd. for C_25_H_33_FN_3_O_5_ (M + H)^+^ 474.2404 and found 474.2425.

***tert*-Butyl *N*-[{[(t*ert*-butoxy)carbonyl]amino}({[(3-{[2-chloro-3-(trifluoromethyl) phenyl]methoxy}phenyl)methyl]amino})methylidene]carbamate (13m):** A white solid was obtained (76% yield), m.p. 100–102 °C. ^1^H NMR (400 MHz, CDCl_3_): δ 1.49 (9H, s, *t*-Bu), 1.52 (9H, s, *t*-Bu), 4.73 (2H, d, *J* = 5.2 Hz, CH_2_), 5.21 (2H, s, CH_2_), 6.91 (1H, d, *J* = 7.9 Hz, ArH), 6.97 (1H, d, *J* = 7.9 Hz, ArH), 6.99 (1H, s, ArH), 7.29 (1H, t, *J* = 7.8 Hz, ArH), 7.40 (1H, t, *J* = 7.8 Hz, ArH), 7.68 (1H, d, *J* = 7.8 Hz, ArH), 7.79 (1H, t, *J* = 7.8 Hz, ArH), 8.65 (1H, br.s, NH), and 11.55 (1H, s, NH). HRMS (ESI): Calcd. for C_26_H_32_ClF_3_N_3_O_5_ (M + H)^+^ 558.1983 and found 558.1971.

***tert*-Butyl *N*-[{[(*tert*-butoxy)carbonyl]amino}[({3-[(3-chlorophenyl)methoxy]-4-methoxyphenyl}methyl)amino]methylidene]carbamate (13n):** A white solid was obtained (82% yield), m.p. 79–81 °C. ^1^H NMR (400 MHz, CDCl_3_): δ 1.47 (9H, s, *t*-Bu), 1.51 (9H, s, *t*-Bu), 3.87 (3H, s, OCH_3_), 4.49 (2H, d, *J* = 5.1 Hz, CH_2_), 5.09 (2H, s, CH_2_), 6.75–6.80 (3H, m, 3 × ArH), 7.25–7.35 (3H, m, 3 × ArH), 7.45 (1H, s, ArH), 8.49 (1H, br.s, NH), and 11.52 (1H, s, NH). HRMS (ESI): Calcd. for C_26_H_34_ClN_3_NaO_6_ (M + Na)^+^ 542.2034 and found 542.2014.

***tert*-Butyl *N*-[{[(*tert*-butoxy)carbonyl]amino}[({3-[(4-chlorophenyl)methoxy]-4-methoxyphenyl}methyl)amino]methylidene]carbamate (13o):** A white solid was obtained (79% yield), m.p. 126–128 °C. ^1^H NMR (400 MHz, CDCl_3_): δ 1.47 (9H, s, *t*-Bu), 1.51 (9H, s, *t*-Bu), 3.80 (3H, s, OCH_3_), 4.49 (2H, d, *J* = 5.1 Hz, CH_2_), 5.09 (2H, s, CH_2_), 6.85–6.90 (3H, m, 3 × ArH), 7.30–7.40 (4H, m, 4 × ArH), 8.49 (1H, br.s, NH), and 11.53 (1H, s, NH). HRMS (ESI): Calcd. for C_26_H_34_ClN_3_NaO_6_ (M + Na)^+^ 542.2034 and found 542.2050.

***tert*-Butyl *N*-[{[(*tert*-butoxy)carbonyl]amino}[({3-[(2,3-dichlorophenyl)methoxy]-2,6-difluorophenyl}methyl)amino]methylidene]carbamate (13p):** A white solid was obtained (65% yield), m.p. 140–142 °C. ^1^H NMR (400 MHz, CDCl_3_): δ 1.47 (9H, s, *t*-Bu), 1.52 (9H, s, *t*-Bu), 4.74 (2H, d, *J* = 5.1 Hz, CH_2_), 5.19 (2H, s, CH_2_), 6.82–6.89 (2H, m, 2 × ArH), 7.25 (1H, t, *J* = 8.2 Hz, ArH), 7.42 (1H, d, *J* = 8.2 Hz, ArH), 7.50 (1H, d, *J* = 8.1 Hz, ArH), 8.52 (1H, br.s, NH), and 11.54 (1H, s, NH). HRMS (ESI): Calcd. for C_25_H_30_Cl_2_F_2_N_3_O_5_ (M + H)^+^ 560.1530 and found 560.1511.

***tert*-Butyl *N*-[{[(tert-butoxy)carbonyl]amino}({[(2,6-difluoro-3-{[3-(trifluoromethyl) phenyl]methoxy}phenyl)methyl]amino})methylidene]carbamate (13q):** A white solid was obtained (62% yield), m.p. 85–86 °C. ^1^H NMR (400 MHz, CDCl_3_): δ 1.46 (9H, s, *t*-Bu), 1.51 (9H, s, *t*-Bu), 4.72 (2H, d, *J* = 5.1 Hz, CH_2_), 5.13 (2H, s, CH_2_), 6.82–6.90 (2H, m, 2 × ArH), 7.51 (1H, t, *J* = 8.1 Hz, ArH), 7.59–7.63 (2H, m, 2 × ArH), 7.69 (1H, s, ArH), 8.47 (1H, br.s, NH), and 11.53 (1H, s, NH). HRMS (ESI): Calcd. for C_26_H_31_F_5_N_3_O_5_ (M + H)^+^ 560.2184 and found 560.2172.

**General Procedure: Synthesis of benzyl guanidine derivatives (9a–q):** In a solution of the substituted *N,N*′-di-Boc-(guanydinomethyl)benzene (**13a–q**) (0.3 mmol) in CH_2_Cl_2_ (2 mL), TFA (1 mL) was added. The mixture was shaken at r.t. overnight and then evaporated to dryness. Et_2_O (1 mL) was added, and the precipitate was collected, washed with Et_2_O, and dried in vacuo to give **9a–q** as a white or off-white solid.

**1-(3-(Benzyloxy)benzyl)guanidinium 2,2,2-trifluoroacetate (9a):** A white solid was obtained (95% yield). ^1^H NMR (400 MHz, CD_3_OD): *δ* 4.26 (2H, s, CH_2_), 4.98 (2H, s, CH_2_), 6.80–6.95 (2H, m, 2 × ArH), 7.18–7.29 (2H, m, 2 × ArH), 7.24 (2H, m, 2 × ArH), and 7.33 (2H, m, 2 × ArH). HRMS (ESI): Calcd. for C_15_H_18_N_3_O (M + H)^+^ 256.1450 and found 256.1454.

**1-(3-(4-Chlorobenzyloxy)benzyl)guanidinium 2,2,2-trifluoroacetate (9b):** An off-white solid was obtained (96% yield). ^1^H NMR (400 MHz, CD_3_OD): *δ* 4.39 (2H, s, CH_2_), 5.16 (2H, s, CH_2_), 6.98–7.05 (3H, m, 3 × ArH), 7.37 (1H, t, *J* = 8.3 Hz, ArH), and 7.43–7.50 (4H, m, 4 × ArH). HRMS (ESI): Calcd. for C_15_H_17_ClN_3_O (M + H)^+^ 290.1060 and found 290.1066.

**1-(3-(3-Chlorobenzyloxy)benzyl)guanidinium 2,2,2-trifluoroacetate (9c):** An off-white solid was obtained (85% yield). ^1^H NMR (400 MHz, CD_3_OD): *δ* 4.38 (2H, s, CH_2_), 5.16 (2H, s, CH_2_), 6.98–7.05 (3H, m, 3 × ArH), 7.32–7.40 (4H, m, 4 × ArH), and 7.51 (1H, s, ArH). HRMS (ESI): Calcd. for C_15_H_17_ClN_3_O (M + H)^+^ 290.1060 and found 290.1067.

**1-(3-(2,4-Dichlorobenzyloxy)benzyl)guanidinium 2,2,2-trifluoroacetate (9d):** A white solid was obtained (98% yield). ^1^H NMR (400 MHz, DMSO-*d_6_*): *δ* 4.40 (2H, d, *J* = 5.0 Hz, CH_2_), 5.20 (2H, s, CH_2_), 6.95–7.05 (3H, m, 3 × ArH), 7.38 (1H, t, *J* = 8.0 Hz, ArH), 7.55 (1H, dd, *J* = 7.9, 1.9 Hz, ArH), 7.67 (1H, d, *J* = 7.9 Hz, ArH), 7.78 (1H, t, *J* = 2.1 Hz, ArH), and 8.05 (1H, br.s, NH). HRMS (ESI): Calcd. for C_15_H_16_Cl_2_N_3_O (M + H)^+^ 324.0670 and found 324.0665.

**1-(3-(3,4-Dichlorobenzyloxy)benzyl)guanidinium 2,2,2-trifluoroacetate (9e):** A white solid was obtained (99% yield). ^1^H NMR (400 MHz, DMSO-*d_6_*): *δ* 4.39 (2H, d, *J* = 5.1 Hz, CH_2_), 5.19 (2H, s, CH_2_), 6.93–7.05 (3H, m, ArH), 7.38 (1H, td, *J* = 7.9, 1.9 Hz, ArH), 7.50 (1H, dd, *J* = 8.0, 1.9 Hz, ArH), 7.72 (1H, d, *J* = 8.1 Hz, ArH), 7.78 1H, (t, *J* = 1.9 Hz, ArH), and 8.02 (1H, br.s, NH). HRMS (ESI): Calcd. for C_15_H_16_Cl_2_N_3_O (M + H)^+^ 324.0670 and found 324.0667.

**1-(3-(2,5-Dichlorobenzyloxy)benzyl)guanidinium 2,2,2-trifluoroacetate (9f):** A white solid was obtained (98% yield). ^1^H NMR (400 MHz, DMSO-*d_6_*): *δ* 4.42 (2H, d, *J* = 6.0 Hz, CH_2_), 5.20 (2H, s, CH_2_), 6.95–7.08 (3H, m, 3 × ArH), 7.40 (1H, td, *J* = 8.0, 1.8 Hz, ArH), 7.55 (1H, dd, *J* = 8.0, 2.1 Hz, ArH), 7.63 (1H, d, *J* = 8.0 Hz, ArH), 7.72 (1H, t, *J* = 2.1 Hz, ArH), and 8.10 (1H, br.s, NH). HRMS (ESI): Calcd. for C_15_H_16_Cl_2_N_3_O (M + H)^+^ 324.0670 and found 324.0675.

**1-(3-(2,3-Dichlorobenzyloxy)benzyl)guanidinium 2,2,2-trifluoroacetate (9g):** A white solid was obtained (92% yield). ^1^H NMR (400 MHz, DMSO-*d_6_*): *δ* 4.40 (2H, d, *J* = 5.5 Hz, CH_2_), 5.25 (2H, s, CH_2_), 6.95–7.08 (3H, m, 3 × ArH), 7.40 (1H, td, *J* = 8.0, 1.8 Hz, ArH), 7.48 (1H, t, *J* = 8.0 Hz, ArH), 7.63 (1H, dd, *J* = 8.0, 1.8 Hz, ArH), 7.72 (1H, dd, *J* = 8.0, 1.8 Hz, ArH), and 8.05 (1H, br.s, NH). ^13^C NMR (100 MHz, DMSO-*d_6_*): *δ* 43.9, 67.3, 113.6, 113.9, 120.0, 128.5, 129.9, 130.3, 130.5, 132.0, 136.9, 139.0, 156.8, and 157.2. (HRMS (ESI): Calcd. for C_15_H_16_Cl_2_N_3_O (M + H)^+^ 324.0670 and found 324.0661.

**1-(3-(2,3-Dichlorobenzyloxy)benzyl)guanidinium chloride (9g.HCl):** Compound **9g** (5 mg) was converted to the hydrogen chloride salt by dissolving in a HCl-methanol (0.5M, 2 mL) solution and concentrating *in vacuo.* A white solid was obtained (4 mg). HRMS (ESI): Calcd. for C_15_H_16_Cl_2_N_3_O (M + H)^+^ 324.0670 and found 324.0677.

**1-(3-[4-(Trifluoromethyl)benzyloxy]benzyl)guanidinium 2,2,2-trifluoroacetate (9h):** A white solid was obtained (97% yield). ^1^H NMR (400 MHz, DMSO-*d_6_*): *δ* 4.40 (d2H, *J* = 5.5 Hz, CH_2_), 5.25 (2H, s, CH_2_), 6.95–7.12 (2H, m, 2 × ArH), 7.38 (2H, m, 2 × ArH), 7.72 (2H, d, *J* = 8.2 Hz, 2 × ArH), 7.82 (2H, d, *J* = 8.2 Hz, 2 × ArH), and 8.05 (1H, br.s, NH). HRMS (ESI): Calcd. for C_26_H_33_F_3_N_3_O_5_ (M + H)^+^ 524.2372 and found 524.2360.

**1-(3-[3-(Trifluoromethyl)benzyloxy]benzyl)guanidinium 2,2,2-trifluoroacetate (9i):** An off-white solid was obtained (98% yield). ^1^H NMR (400 MHz, DMSO-*d_6_*): *δ* 4.40 (2H, d, *J* = 6.1 Hz, CH_2_), 5.27 (2H, s, CH_2_), 6.95–7.08 (3H, m, 3 × ArH), 7.13 (1H, td, *J* = 7.9, 1.7 Hz, ArH), 7.71 (1H, t, *J* = 7.9 Hz, ArH), 7.78 (1H, d, *J* = 8.0 Hz, ArH), 7.82 (1H, d, *J* = 8.0 Hz, ArH), 7.87 (1H, s, ArH), and 8.05 (1H, br.s, NH). HRMS (ESI): Calcd. for C_26_H_33_F_3_N_3_O_5_ (M + H)^+^ 524.2372 and found 524.2397.

**1-(3-(4-Bromobenzyloxy)benzyl)guanidinium 2,2,2-trifluoroacetate (9j):** A white solid was obtained (97% yield). ^1^H NMR (400 MHz, DMSO-*d_6_*): *δ* 4.39 (2H, d, *J* = 6.2 Hz, CH_2_), 5.14 (2H, s, CH_2_), 6.95–7.08 (3H, m, 3 × ArH), 7.37 (1H, t, *J* = 8.0 Hz, ArH), 7.46 (2H, d, *J* = 8.0 Hz, 2 × ArH), 7.65 (2H, d, *J* = 8.1 Hz, 2 × ArH), and 8.10 (1H, br.s, NH). HRMS (ESI): Calcd. for C_15_H_16_BrN_3_NaO (M + Na)^+^ 356.0374 and found 356.0375.

**1-(3-(4-Fluorobenzyloxy)benzyl)guanidinium 2,2,2-trifluoroacetate (9k):** A white solid was obtained (90% yield). ^1^H NMR (400 MHz, DMSO-*d_6_*): *δ* 4.40 (2H, d, *J* = 6.2 Hz, CH_2_), 5.11 (2H, s, CH_2_), 6.97–7.10 (3H, m, 3 × ArH), 7.35 (1H, td, *J* = 8.0, 1.5 Hz, ArH), 7.45 (2H, d, *J* = 8.2 Hz, 2 × ArH), 7.60 (2H, d, *J* = 8.2 Hz, 2 × ArH), and 8.07 (1H, br.s, NH). HRMS (ESI): Calcd. for C_15_H_17_FN_3_O (M + H)^+^ 274.1356 and found 274.1366.

**1-(3-[2-Chloro-3-(trifluoromethyl)benzyloxy]benzyl)guanidinium 2,2,2-trifluoroacetate (9m):** A white solid was obtained (99% yield). ^1^H NMR (400 MHz, DMSO-*d_6_*): *δ* 4.41 (2H, d, *J* = 6.1 Hz, CH_2_), 5.31 (2H, s, CH_2_), 6.96–7.08 (3H, m, 3 × ArH), 7.40 (1H, t, *J* = 8.0 Hz, ArH), 7.68 (1H, t, *J* = 8.1 Hz, ArH), 7.95 (1H, d, *J* = 7.9 Hz, ArH), 7.99 (1H, d, *J* = 7.9 Hz, ArH), and 8.12 (1H, br.s, NH). HRMS (ESI): Calcd. for C_16_H_16_ClF_3_N_3_O (M + H)^+^ 358.0934 and found 358.0979.

**1-(3-[2-Chloro-3-(trifluorobenzyloxy)benzyl]guanidinium chloride (9m.HCl):** Compound **9m** (9 mg) was converted to the hydrogen chloride salt by dissolving in a HCl-methanol (0.5 M, 2 mL) solution and concentrating *in vacuo.* A white solid was obtained (7 mg). HRMS (ESI): Calcd. for C_16_H_16_ClF_3_N_3_O (M + H)^+^ 358.0934 and found 358.0897.

**1-(3-[3-Chlorobenzyloxy]-4-methoxybenzyl)guanidinium 2,2,2-trifluoroacetate (9n):** A white solid was obtained (97% yield). ^1^H NMR (400 MHz, DMSO-*d_6_*): *δ* 4.36 (2H, d, *J* = 6.0 Hz, CH_2_), 5.27 (2H, s, CH_2_), 6.90–7.15 (3H, m, 3 × ArH), 7.39–7.45 (3H, m, 3 × ArH), 7.65 (1H, s, ArH), and 8.12 (1H, br.s, NH). HRMS (ESI): Calcd. for C_16_H_18_ClN_3_NaO_2_ (M + Na)^+^ 342.0985 and found 342.0977.

**1-(3-[4-Chlorobenzyloxy]-4-methoxybenzyl)guanidinium 2,2,2-trifluoroacetate (9o):** An off-white solid was obtained (95% yield). ^1^H NMR (400 MHz, DMSO-*d_6_*): *δ* 4.40 (2H, d, *J* = 6.0 Hz, CH_2_), 5.29 (2H, s, CH_2_), 6.87–7.02 (3H, m, 3 × ArH), 7.30–7.39 (4H, m, 4 × ArH), and 8.07 (1H, br.s, NH). HRMS (ESI): Calcd. for C_16_H_18_ClN_3_NaO_2_ (M + Na)^+^ 342.0985 and found 342.0994.

**1-(3-(2,3-Dichlorobenzyloxy]-2,6-difluorobenzyl)guanidinium 2,2,2-trifluoroacetate (9p):** A white solid was obtained (93% yield). ^1^H NMR (400 MHz, DMSO-*d_6_*): *δ* 4.50 (2H, s, CH_2_), 5.35 (2H, s, CH_2_), 7.30–7.40 (2H, m, 2 × ArH), 7.50 (1H, t, *J* = 7.9 Hz, ArH), 7.63 (1H, d, *J* = 7.9 Hz, ArH), 7.75 (1H, d, *J* = 8.0 Hz, ArH), and 7.96 (1H, br.s, NH). HRMS (ESI): Calcd. for C_15_H_14_Cl_2_F_2_N_3_O (M + H)^+^ 360.0482 and found 360.0493.

**1-(2,6-Difluoro-3-[3-(trifluoromethyl)benzyloxy]benzyl)guanidinium 2,2,2-trifluoroacetate (9q):** A white solid was obtained (98% yield). ^1^H NMR (400 MHz, DMSO-*d_6_*): *δ* 4.49 (2H, d, *J* = 5.3 Hz, CH_2_), 5.35 (2H, s, CH_2_), 7.18 (1H, dt, *J* = 7.9, 1.6 Hz, ArH), 7.32–7.40 (2H, m, 2 × ArH), 7.72 (1H, t, *J* = 8.1 Hz, ArH), 7.81 (1H, d, *J* = 7.9 Hz, ArH), 7.88 (1H, s, ArH), and 8.00 (1H, br.s, NH). HRMS (ESI): Calcd. for C_16_H_15_F_5_N_3_O (M + H)^+^ 360.1135 and found 360.1248.

***tert*-Butyl *N*-[{[(*tert*-butoxy)carbonyl]imino}({[(4-hydroxyphenyl)methyl]amino}) methyl]carbamate** (**15**): In a solution of 4-(aminomethyl)phenol hydrochloride **14** (6.0 mmol) in DMF (20 mL), *S*-methyl-*N*,*N*’-bis(*tert*-butoxycarbonyl)isothiourea (1.6 g, 5.6 mmol) was added, followed by Et_3_N (2.0 mL). The mixture was stirred at r.t. overnight and partitioned between EtOAc (100 mL) and citric acid (50 mL, 5% in water). The organic layer was washed with brine, dried (MgSO_4_), and concentrated in vacuo to give an off-white solid. Purification by flash column chromatography (petrol ether to petrol ether/EtOAc 11:9) afforded **15** as a white solid (1.4 g, 68% yield). mp 137–138 °C. ^1^H NMR (400 MHz, CDCl_3_): *δ* 1.47 (9H, s, *t*-Bu), 1.49 (9H, s, *t*-Bu), 4.51 (2H, d, *J* = 5.2 Hz, CH_2_), 6.91 (2H, d, *J* = 8.1 Hz, 2 × ArH), 7.10 (2H, d, *J* = 8.1 Hz, 2 × ArH), 8.50 (1H, br.s, NH), and 11.5 (1H, s, NH). HRMS (ESI): Calcd. for C_18_H_28_N_3_O_5_ (M + H)^+^ 366.2029 and found 366.2037.

**General Procedure: Benzylation of 4-(*N,N*′-di-Boc-guanydinomethyl)phenol (15):** In a solution of 4-(*N,N*′-di-Boc-guanydinomethyl)phenol **15** (0.56 mmol) in acetone (8 mL), the substituted benzyl bromide (0.56 mmol) was added, followed by K_2_CO_3_ (96 mg, 0.7 mmol). The mixture was stirred at r.t. overnight and then partitioned between EtOAc (50 mL) and water (50 mL). The organic layer was washed with brine, dried (MgSO_4_), and concentrated in vacuo to give an off-white solid. Purification by flash column chromatography eluting with a gradient solvent (petrol ether to petrol ether/EtOAc 3:1) afforded **16a–e**.

***tert*-Butyl *N*-[({[4-(benzyloxy)phenyl]methyl}amino)({[(tert-butoxy) carbonyl]-amino})methylidene]carbamate (16a):** A white solid was obtained (82% yield), m.p. 115–116 °C. ^1^H NMR (400 MHz, CDCl_3_): *δ* 1.34 (9H, s, *t*-Bu), 1.49 (9H, s, *t*-Bu), 5.10 (2H, s, CH_2_), 5.15 (2H, br.s, CH_2_), 6.88 (2H, dt, *J* = 8.8, 2.0 Hz, 2 × ArH), 7.19 (2H, dt, *J* = 8.8, 2.1 Hz, 2 × ArH), 7.28–7.44 (5H, m, 5 × ArH), 9.30 (1H, br.s, NH), and 10.5 (1H, br.s, NH). HRMS (ESI): Calcd. for C_25_H_33_N_3_NaO_5_ (M + Na)^+^ 478.2318 and found 478.2329.

***tert*-Butyl *N*-[{[(*tert*-butoxy)carbonyl]amino}[({4-[(4-chlorophenyl)methoxy] phenyl}methyl)amino]methylidene]carbamate (16b):** A white solid was obtained (79% yield), m.p. 120–121 °C. ^1^H NMR (400 MHz, CDCl_3_): *δ* 1.47 (9H, s, *t*-Bu), 1.52 (9H, s, *t*-Bu), 4.65 (2H, br.s, CH_2_), 5.01 (2H, s, CH_2_), 6.92 (2H, d, *J* = 8.2 Hz, 2 × ArH), 7.25 (2H, d, *J* = 8.2 Hz, 2 × ArH), 7.35 (4H, s, 4 × ArH), 8.75 (1H, br.s, NH), and 11.54 (1H, br.s, NH).

***tert*-Butyl *N*-[{[(*tert*-butoxy)carbonyl]amino}[({4-[(3-chlorophenyl)methoxy] phenyl}methyl)amino]methylidene]carbamate (16c):** A white solid was obtained (67% yield), m.p. 120–122 °C. ^1^H NMR (400 MHz, CDCl_3_): *δ* 1.47 (9H, s, *t*-Bu), 1.52 (9H, s, *t*-Bu), 4.60 (2H, br.s, CH_2_), 5.02 (2H, s, CH_2_), 6.93 (2H, dd, *J* = 7.1, 2.0 Hz, 2 × ArH), 7.20–7.30 (5H, m, 5 × ArH), 7.43 (1H, s, ArH), 8.62 (1H, br.s, NH), and 11.5 (1H, br.s, NH).

***tert*-Butyl *N*-[{[(*tert*-butoxy)carbonyl]amino}[({4-[(3,4-dichlorophenyl)methoxy] phenyl}methyl)amino]methylidene]carbamate (16d):** A white solid was obtained (79% yield), m.p. 156–158 °C. ^1^H NMR (400 MHz, CDCl_3_): *δ* 1.47 (9H, s, *t*-Bu), 1.52 (9H, s, *t*-Bu), 4.63 (2H, br.s, CH_2_), 5.00 (2H, s, CH_2_), 7.22 (1H, d, *J* = 7.9 Hz, ArH), 7.43 (1H, d, *J* = 8.1 Hz, ArH), 7.51 (1H, d, *J* = 1.6 Hz, ArH), 6.91 (2H, d, *J* = 7.9 Hz, 2 × ArH), 8.80 (1H, br.s, NH), and 11.5 (1H, br.s, NH).

***tert*-Butyl *N*-[{[(*tert*-butoxy)carbonyl]amino}[({4-[(2,3-dichlorophenyl)methoxy] phenyl}methyl)amino]methylidene]carbamate (16e):** A white solid was obtained (89% yield), m.p. 99–101 °C. ^1^H NMR (400 MHz, CDCl_3_): *δ* 1.48 (9H, s, *t*-Bu), 1.53 (9H, s, *t*-Bu), 4.73 (2H, br.s, CH_2_), 5.16 (2H, s, CH_2_), 6.95 (2H, d, *J* = 8.1 Hz, 2 × ArH), 7.25–7.31 (3H, m, 3 × ArH), 7.43 (1H, d, *J* = 8.2 Hz, ArH), 7.47 (1H, d, *J* = 8.2 Hz, ArH), 8.90 (1H, br.s, NH), and 11.6 (1H, br.s, NH).

**General Procedure: Synthesis of benzyl guanidine derivatives (9r–v):** In a solution of the *para*-substituted *N,N*′-di-Boc-(4-guanidinomethyl)benzene (100 mg) (**16a–e)** in CH_2_Cl_2_ (2 mL), TFA (1 mL) was added. The mixture was shaken at r.t. overnight and then evaporated to dryness. Et_2_O (1 mL) was added, and the precipitate was collected, washed with ether, and dried in vacuo to give **9r–v** as a white or off-white solid.

**1-(4-(Benzyloxy)benzyl)guanidinium 2,2,2-trifluoroacetate (9r):** A off-white solid was obtained (96% yield). ^1^H NMR (400 MHz, CD_3_OD): *δ* 4.38 (2H, br.s, CH_2_), 5.16 (2H, s, CH_2_), 7.06 (2H, d, *J* = 8.3 Hz, 2 × ArH), 7.32 (2H, d, *J* = 8.3 Hz, 2 × ArH), 7.36–7.43 (3H, m, 3 × ArH), and 7.50 (2H, d, *J* = 8.2 Hz, 2 × ArH). HRMS (ESI): Calcd. for C_15_H_18_N_3_O (M + H)^+^ 256.1450 and found 256.1539.

**1-(4-(4-Chlorobenzyloxy)benzyl)guanidinium 2,2,2-trifluoroacetate (9s):** A white solid was obtained (87% yield). ^1^H NMR (400 MHz, DMSO-*d_6_*): *δ* 4.55 (2H, br.s, CH_2_), 5.17 (2H, s, CH_2_), 6.94 (2H, d, *J* = 8.2 Hz, 2 × ArH), 7.27 (2H, d, *J* = 8.1 Hz, 2 × ArH), 7.37–7.41 (4H, m, 4 × ArH), and 8.15 (br s, 1H, NH). HRMS (ESI): Calcd. for C_15_H_17_ClN_3_O (M + H)^+^ 290.1060 and found 290.1066.

**1-(4-(3-Chlorobenzyloxy)benzyl)guanidinium 2,2,2-trifluoroacetate (9t):** A white solid was obtained (93% yield). ^1^H NMR (400 MHz, DMSO-*d_6_*): *δ* 4.33 (2H, br.s, CH_2_), 5.19 (2H, s, CH_2_), 7.08 (3H, m, 3 × ArH), 7.30 (2H, m, 2 × ArH), 7.40–7.50 (3H, m, 3 × ArH), and 7.95 (1H, br.s, NH). HRMS (ESI): Calcd. for C_15_H_17_ClN_3_O (M + H)^+^ 290.1060 and found 290.1071.

**1-(4-(3,4-Dichlorobenzyloxy)benzyl)guanidinium 2,2,2-trifluoroacetate (9u):** A white solid was obtained (93% yield). ^1^H NMR (400 MHz, DMSO-*d_6_*): *δ* 4.33 (2H, d, *J* = 6.0 Hz, CH_2_), 5.19 (2H, s, CH_2_), 7.09 (2H, d, *J* = 8.6 Hz, 2 × ArH), 7.31 (2H, d, *J* = 8.5 Hz, 2 × ArH), 7.50 (1H, dd, *J* = 7.8, 2.1 Hz, 2 × ArH), 7.72 (1H, d, *J* = 7.7 Hz, ArH), 7.77 (1H, d, *J* = 2.0 Hz, ArH), and 7.87 (1H, br.s, NH). HRMS (ESI): Calcd. for C_15_H_15_Cl_2_N_3_O (M + H)^+^ 324.0670 and found 324.0658.

**1-(4-(2,3-Dichlorobenzyloxy)benzyl)guanidinium 2,2,2-trifluoroacetate (9v):** A white solid was obtained (98% yield). ^1^H NMR (400 MHz, DMSO-*d_6_*): *δ* 4.38 (2H, br.s, CH_2_), 5.35 (2H, s, CH_2_), 7.14 (2H, d, *J* = 8.2 Hz, 2 × ArH), 7.32 (2H, d, *J* = 8.3 Hz, 2 × ArH), 7.46 (1H, t, *J* = 7.9 Hz, ArH), 7.63 (1H, d, *J* = 8.1 Hz, ArH), 7.72 (1H, d, *J* = 7.9 Hz, ArH), and 8.05 (1H, br.s, NH). ^13^C NMR (DMSO-*d_6_*): *δ* 43.5, 67.3, 114.9, 128.4, 128.4, 128.9, 130.2, 130.5, 132.0, 137.0, 156.8, and 157.5. HRMS (ESI): Calcd. for C_15_H_15_Cl_2_N_3_O (M + H)^+^ 324.0670 and found 324.0720.

**General procedure: Synthesis of Boc-protected aminobenzyl guanidine derivatives (18**, **22):** 3-Aminobenzylamine **17** or 4-aminobenzylamine **21** (10 mmol) was dissolved in DMF (8 mL). *S*-methyl-*N*,*N*’-bis(*tert*-butoxycarbonyl)isothiourea (10.5 mmol) and Et_3_N (20 mmol) were added successively at 0 °C. The reaction mixture was stirred for 18 h at r.t. and then evaporated at 70 °C. Water (180 mL) and brine (20 mL) were added, and the mixture was extracted with Et_2_O (2 × 200 mL). The organic layer was dried (Na_2_SO_4_), filtered, and concentrated *in vacuo.* Purification by flash column chromatography (CH_2_Cl_2_, 100%) gave **18** and **22**.

***tert*-Butyl *N*-[(1*E*)-{[(3-aminophenyl)methyl]amino}({[(*tert*-butoxy)carbonyl] imino})methyl]carbamate (18):** 2.85 g, 78%, white foam. ^1^H NMR (400 MHz, DMSO-*d_6_*): *δ* 1.44 (9H, s, *t*-Bu), 1.53 (9H, s, *t*-Bu), 4.41 (2H, d, *J* = 5.6 Hz, CH_2_), 5.14 (2H, br.s, NH_2_), 6.46 (1H, d, *J* = 7.2 Hz, ArH), 6.50 (1H, s, ArH), 6.51 (1H, d, *J* = 7.2 Hz, ArH), 7.02 (1H, t, *J* = 8.0 Hz, ArH), 8.56 (1H, t, *J* = 5.6 Hz, NH), and 11.60 (1H, br.s, NH). HRMS (ESI): Calcd. for C_18_H_29_N_4_O_4_ (M + H)^+^ 365.2183 and found 365.2189.

***tert*-Butyl *N*-[(1*E*)-{[(4-aminophenyl)methyl]amino}({[(*tert*-butoxy)carbonyl] imino})methyl]carbamate (22):** 2.98 g, 81%, white foam. ^1^H NMR (400 MHz, DMSO-*d_6_*): *δ* 1.45 (9H, s, *t*-Bu), 1.50 (9H, s, *t*-Bu), 4.34 (2H, d, *J* = 5.6 Hz, CH_2_), 5.10 (2H, br.s, NH_2_), 6.58 (2H, d, *J* = 7.6 Hz, 2 × ArH), 7.03 (2H, d, *J* = 8.0 Hz, 2 × ArH), 8.41 (1H, t, *J* = 5.6 Hz, NH), and 11.55 (1H, br.s, NH). HRMS (ESI): Calcd. for C_18_H_29_N_4_O_4_ (M + H)^+^ 365.2183 and found 365.2187.

**General Procedure: Synthesis of Boc-protected sulphonamide derivatives (19a–d, 23a–d):** Compound **18** or **22** (0.2 mmol) was dissolved in CH_2_Cl_2_ (1.2 mL) and pyridine (0.8 mL) at 0 °C. The corresponding benzene sulphonyl chloride (0.22 mmol) was added, and the reaction mixture was stirred for 2 h at 0 °C. Water (40 mL) and brine (10 mL) were added, and the mixture was extracted with Et_2_O (2 × 50 mL). The organic layer was dried (Na_2_SO_4_), filtered, and concentrated to dryness. The residue was then co-evaporated with toluene (2 × 5 mL) and CH_2_Cl_2_ (2 × 5 mL) to give **19a–d** or **23a–d**.

***tert*-Butyl *N*-[(1*E*)-{[(*tert*-butoxy)carbonyl]imino}({[3-(3-chlorobenzene sulfonamido)phenyl]methyl}amino)methyl]carbamate (19a):** 99 mg, 92%, white foam. ^1^H NMR (400 MHz, CDCl_3_): *δ* 1.47 (9H, s, *t*-Bu), 1.48 (9H, s, *t*-Bu), 4.51 (2H, s, CH_2_), 6.98–7.06 (3H, m, 3 × ArH), 7.18 (1H, t, *J* = 8.0 Hz, ArH), 7.34 (1H, t, *J* = 7.8 Hz, ArH), 7.46 (1H, ddd, *J* = 8.0, 2.0, 0.8 Hz, ArH), 7.54 (1H, br.s, NH), 7.64 (1H, ddd, *J* = 7.8, 1.6, 1.2 Hz, ArH), 7.79 (1H, t, *J* = 1.8 Hz, ArH), 8.60 (1H, br.s, NH), and 11.52 (1H, br.s, NH). HRMS (ESI): Calcd. for C_24_H_32_ClN_4_O_6_S (M + H)^+^ 539.1726 and found 539.1737.

***tert*-Butyl *N*-[(1*E*)-{[(*tert*-butoxy)carbonyl]imino}({[3-(4-chlorobenzene sulfonamido)phenyl]methyl}amino)methyl]carbamate (19b):** 98 mg, 91%, foam. ^1^H NMR (400 MHz, CDCl_3_): *δ* 1.47 (9H, s, *t*-Bu), 1.48 (9H, s, *t*-Bu), 4.53 (2H, s, CH_2_), 7.00 (1H, d, *J* = 2.4 Hz, ArH), 7.02 (1H, d, *J* = 2.4 Hz, ArH), 7.07 (1H, s, ArH), 7.17 (1H, t, *J* = 7.8 Hz, ArH), 7.35 (2H, dt, *J* = 8.8, 2.2 Hz, 2 × ArH), 7.65 (1H, br.s, NH), 7.70 (2H, dt, *J* = 8.8, 2.2 Hz, 2 × ArH), 8.61 (1H, br.s, NH), and 11.52 (1H, br.s, NH). HRMS (ESI): Calcd. for C_24_H_32_ClN_4_O_6_S (M + H)^+^ 539.1726 and found 539.1738.

***tert*-Butyl *N*-[(1*E*)-{[(*tert*-butoxy)carbonyl]imino}({[3-(2,3-dichlorobenzene sulfonamido)phenyl]methyl}amino)methyl]carbamate (19c):** 107 mg, 93%, yellow foam. ^1^H NMR (400 MHz, CDCl_3_): *δ* 1.48 (9H, s, *t*-Bu), 1.49 (9H, s, *t*-Bu), 4.55 (2H, s, CH_2_), 6.98–7.04 (2H, m, 2 × ArH), 7.08 (1H, s, ArH), 7.17 (1H, t, *J* = 7.8 Hz, ArH), 7.27 (1H, t, *J* = 8.0 Hz, ArH), 7.46 (1H, br.s, NH), 7.60 (1H, d, *J* = 8.0 Hz, ArH), 7.97 (1H, d, *J* = 8.0 Hz, ArH), 8.61 (1H, br.s, NH), and 11.52 (1H, br.s, NH). HRMS (ESI): Calcd. for C_24_H_31_Cl_2_N_4_O_6_S (M + H)^+^ 573.1336 and found 573.1349.

***tert*-Butyl *N*-[(1*E*)-{[(*tert*-butoxy)carbonyl]imino}[({3-[3-(trifluoromethyl) benzenesulfonamido]phenyl}methyl)amino]methyl]carbamate (19d):** 107 mg, 93%, colourless glass. ^1^H NMR (400 MHz, CDCl_3_): *δ* 1.47 (18H, s, 2 × *t*-Bu), 4.52 (2H, s, CH_2_), 6.99–7.07 (3H, m, 3 × ArH), 7.18 (1H, t, *J* = 7.8 Hz, ArH), 7.55 (1H, t, *J* = 7.8 Hz, ArH), 7.68 (1H, br.s, NH), 7.75 (1H, d, *J* = 7.6 Hz, ArH), 7.95 (1H, d, *J* = 7.6 Hz, ArH), 8.05 (1H, s, ArH), 8.62 (1H, br.s, NH), and 11.51 (1H, br.s, NH). HRMS (ESI): Calcd. for C_25_H_32_F_3_N_4_O_6_S (M + H)^+^ 573.1989 and found 573.1998.

***tert*-Butyl *N*-[(1*E*)-{[(*tert*-butoxy)carbonyl]imino}({[4-(3-chloro benzenesulfonamido)phenyl]methyl}amino)methyl]carbamate (23a):** 105 mg, 97%, pale yellow foam. ^1^H NMR (400 MHz, CDCl_3_): *δ* 1.46 (9H, s, *t*-Bu), 1.47 (9H, s, *t*-Bu), 4.54 (2H, s, CH_2_), 7.07 (2H, d, *J* = 8.0 Hz, 2 × ArH), 7.13 (2H, d, *J* = 8.4 Hz, 2 × ArH), 7.34 (1H, t, *J* = 8.0 Hz, ArH), 7.47 (1H, dd, *J* = 8.0, 1.2 Hz, ArH), 7.65 (1H, d, *J* = 7.6 Hz, ArH), 7.73 (1H, br.s, NH), 7.78 (1H, s, ArH), 8.61 (1H, br.s, NH), and 11.50 (1H, br.s, NH). HRMS (ESI): Calcd. for C_24_H_32_ClN_4_O_6_S (M + H)^+^ 539.1726 and found 539.1739.

***tert*-Butyl *N*-({[(*tert*-butoxy)carbonyl]imino}({[4-(4-chlorobenzenesulfonamido) phenyl]methyl}amino)methyl)carbamate (23b):** 103 mg, 95%, white foam. ^1^H NMR (400 MHz, CDCl_3_): *δ* 1.46 (9H, s, *t*-Bu), 1.47 (9H, s, *t*-Bu), 4.55 (2H, s, CH_2_), 7.06 (2H, d, *J* = 8.4 Hz, 2 × ArH), 7.14 (2H, d, *J* = 8.4 Hz, 2 × ArH), 7.38 (2H, dt, *J* = 8.8, 2.2 Hz, 2 × ArH), 7.60 (1H, br.s, NH), 7.71 (2H, dd, *J* = 8.8, 2.2 Hz, 2 × ArH), 8.58 (1H, br.s, NH), and 11.51 (1H, br.s, NH). HRMS (ESI): Calcd. for C_24_H_32_ClN_4_O_6_S (M + H)^+^ 539.1726 and found 539.1741.

***tert*-Butyl *N*-({[(*tert*-butoxy)carbonyl]imino}({[4-(2,3-dichlorobenzene sulfonamido)phenyl]methyl}amino)methyl)carbamate (23c):** 113 mg, 98%, yellow foam. ^1^H NMR (400 MHz, CDCl_3_): *δ* 1.45 (9H, s, *t*-Bu), 1.47 (9H, s, *t*-Bu), 4.51 (2H, s, CH_2_), 7.08 (2H, d, *J* = 8.8 Hz, 2 × ArH), 7.13 (2H, d, *J* = 8.4 Hz, 2 × ArH), 7.27 (1H, t, *J* = 8.2 Hz, ArH), 7.49 (1H, br.s, NH), 7.61 (1H, dd, *J* = 8.0, 1.6 Hz, ArH), 7.93 (1H, dd, *J* = 8.0, 1.6 Hz, ArH), 8.51 (1H, br.s, NH), and 11.50 (1H, br.s, NH). HRMS (ESI): Calcd. for C_24_H_31_Cl_2_N_4_O_6_S (M + H)^+^ 573.1336 and found 573.1347.

***tert*-Butyl* N*-[(1*E*)-{[(*tert*-butoxy)carbonyl]imino}[({4-[3-(trifluoromethyl) benzenesulfonamido]phenyl}methyl)amino]methyl]carbamate (23d):** 111 mg, 97%, pale yellow foam. ^1^H NMR (400 MHz, CDCl_3_): *δ* 1.47 (9H, s, *t*-Bu), 1.48 (9H, s, *t*-Bu), 4.57 (2H, s, CH_2_), 7.07 (2H, d, *J* = 8.4 Hz, 2 × ArH), 7.13 (2H, d, *J* = 8.4 Hz, 2 × ArH), 7.56 (1H, t, *J* = 7.8 Hz, ArH), 7.69 (1H, br.s, NH), 7.76 (1H, d, *J* = 8.0 Hz, ArH), 7.96 (1H, d, *J* = 8.0 Hz, ArH), 8.04 (1H, s, ArH), 8.66 (1H, br.s, NH), and 11.50 (1H, br.s, NH). HRMS (ESI): Calcd. for C_25_H_32_F_3_N_4_O_6_S (M + H)^+^ 573.1989 and found 573.1996.

**General Procedure: Synthesis of Boc-protected amide derivatives (19e, 23e):** Compound **18** or **22** (0.5 mmol) and K_2_CO_3_ (1.0 mmol) were placed in an oven-dried 50 mL glass tube. Acetone (2.0 mL) and benzoyl chloride (0.75 mmol) were added successively, and the reaction mixture was stirred for 18 h at 80 °C. Water (80 mL) and brine (20 mL) were added, and the mixture was extracted with Et_2_O (2 × 100 mL). The organic layer was dried (Na_2_SO_4_), filtered, and concentrated to dryness. Purification by flash column chromatography (petrol ether to petrol ether/EtOAc 4:1) gave **19e** or **23e**.

***tert*-Butyl *N*-[(1*E*)-{[(3-benzamidophenyl)methyl]amino}({[(*tert*-butoxy) carbonyl]-imino})methyl]carbamate (19e):** 45 mg, 19%, colourless glass. ^1^H NMR (400 MHz, CDCl_3_): *δ* 1.47 (9H, s, *t*-Bu), 1.48 (9H, s, *t*-Bu), 4.59 (2H, d, *J* = 5.2 Hz, CH_2_), 7.01 (1H, d, *J* = 7.6 Hz, ArH), 7.30 (1H, t, *J* = 8.0 Hz, ArH), 7.42–7.49 (3H, m, 3 × ArH), 7.53 (1H, t, *J* = 7.6 Hz, CH), 7.70 (1H, t, *J* = 8.0 Hz, ArH), 7.87–7.93 (2H, m, 2 × ArH), 8.19 (1H, s, NH), 8.70 (1H, s, NH), and 11.53 (1H, br.s, NH). HRMS (ESI): Calcd. for C_25_H_33_N_4_O_5_ (M + H)^+^ 469.2445 and found 469.2452.

***tert*-Butyl *N*-[(1*E*)-{[(4-benzamidophenyl)methyl]amino}({[(*tert*-butoxy) carbonyl]-imino})methyl]carbamate (23e):** 27 mg, 11%, colourless glass. ^1^H NMR (400 MHz, CDCl_3_): *δ* 1.47 (9H, s, *t*-Bu), 1.48 (9H, s, *t*-Bu), 4.58 (2H, d, *J* = 5.2 Hz, CH_2_), 7.24 (2H, d, *J* = 8.4 Hz, 2 × ArH), 7.45 (2H, t, *J* = 7.4 Hz, 2 × ArH), 7.52 (1H, t, *J* = 7.2 Hz, ArH), 7.61 (2H, d, *J* = 8.8 Hz, 2 × ArH), 7.87 (2H, d, *J* = 8.4 Hz, 2 × ArH), 8.14 (1H, s, NH), 8.63 (1H, s, NH), and 11.53 (1H, br.s, NH). HRMS (ESI): Calcd. for C_25_H_33_N_4_O_5_ (M + H)^+^ 469.2445 and found 469.2451.

**General procedure: Synthesis of Amino((arylsulfonamido)benzyl) amino) meth-animinium 2,2,2-trifluoroacetate derivatives (20a–d, 24a–d):** Compound **19a–d**, **23a–d** (0.1 mmol) was dissolved in CH_2_Cl_2_ (0.8 mL) and then TFA (0.2 mL) was added. The reaction mixture was stirred for 2 h at r.t. and concentrated to dryness to give **20a–d** and **24a–d.**

**Amino((3-((3-chlorophenyl)sulfonamido)benzyl)amino)methaniminium 2,2,2-trifluoroacetate (20a):** 89 mg, 98%, glass. ^1^H NMR (400 MHz, CD_3_OD): *δ* 4.37 (2H, s, CH_2_), 7.07 (1H, d, *J* = 8.0 Hz, ArH), 7.13 (1H, d, *J* = 7.6 Hz, ArH), 7.23 (1H, s, ArH), 7.33 (1H, t, *J* = 7.8 Hz, ArH), 7.54 (1H, t, *J* = 8.0 Hz, ArH), 7.65 (1H, d, *J* = 8.0 Hz, ArH), 7.75 (1H, d, *J* = 8.0 Hz, ArH), and 7.81 (1H, s ArH). HRMS (ESI): Calcd. for C_14_H_16_ClN_4_O_2_S (M + H)^+^ 339.0677 and found 339.0681.

**Amino((3-((4-chlorophenyl)sulfonamido)benzyl)amino)methaniminium 2,2,2-trifluoroacetate (20b):** 88 mg, 97%, glass. ^1^H NMR (400 MHz, CD_3_OD): *δ* 4.38 (2H, s, CH_2_), 7.07 (1H, d, *J* = 8.0 Hz, ArH), 7.11 (1H, d, *J* = 7.6 Hz, ArH), 7.23 (1H, s, ArH), 7.31 (1H, t, *J* = 8.0 Hz, ArH), 7.54 (2H, d, *J* = 8.4 Hz, 2 × ArH), and 7.81 (2H, d, *J* = 8.4 Hz, 2 × ArH). HRMS (ESI): Calcd. for C_14_H_16_ClN_4_O_2_S (M + H)^+^ 339.0677 and found 339.0685.

**Amino((3-((2,3-dichlorophenyl)sulfonamido)benzyl)amino)methaniminium 2,2,2-trifluoroacetate (20c):** 96 mg, 98%, glass. ^1^H NMR (400 MHz, CD_3_OD): *δ* 4.39 (2H, s, CH_2_), 7.06 (1H, d, *J* = 7.6 Hz, ArH), 7.13 (1H, d, *J* = 8.0 Hz, ArH), 7.22 (1H, s, ArH), 7.30 (1H, t, *J* = 8.0 Hz, ArH), 7.49 (1H, dt, *J* = 8.2, 0.8 Hz, ArH), 7.81 (1H, d, *J* = 8.0 Hz, ArH), and 8.11 (1H, d, *J* = 8.0 Hz, ArH). HRMS (ESI): Calcd. for C_14_H_15_Cl_2_N_4_O_2_S (M + H)^+^ 373.0287 and found 373.0295.

**Amino((3-((3-(trifluoromethyl)phenyl)sulfonamido)benzyl)amino) methaniminium 2,2,2-trifluoroacetate (20d):** 95 mg, 97%, glass. ^1^H NMR (400 MHz, CD_3_OD): *δ* 4.42 (2H, s, CH_2_), 7.07 (1H, d, *J* = 8.0 Hz, ArH), 7.14 (1H, d, *J* = 7.6 Hz, ArH), 7.24 (1H, s, ArH), 7.32 (1H, t, *J* = 8.0 Hz, ArH), 7.77 (1H, d, *J* = 8.2 Hz, ArH), 7.96 (1H, d, *J* = 7.6 Hz, ArH), and 8.07 (2H, s, 2 × ArH). HRMS (ESI): Calcd. for C_15_H_16_F_3_N_4_O_2_S (M + H)^+^ 373.0941 and found 373.0946.

**Amino((4-((3-chlorophenyl)sulfonamido)benzyl)amino)methaniminium 2,2,2-trifluoroacetate (24a):** 90 mg, 99%, glass. ^1^H NMR (400 MHz, CD_3_OD): *δ* 4.38 (2H, s, CH_2_), 7.17–7.22 (2H, m, 2 × ArH), 7.24–7.31 (2H, m, 2 × ArH), 7.53 (1H, t, *J* = 7.4 Hz, ArH), 7.60–7.66 (1H, m, ArH), 7.71–7.77 (1H, m, ArH), and 7.77–7.81 (1H, m, ArH). HRMS (ESI): Calcd. for C_14_H_16_ClN_4_O_2_S (M + H)^+^ 339.0677 and found 339.0685.

**Amino((4-((4-chlorophenyl)sulfonamido)benzyl)amino)methaniminium 2,2,2-trifluoroacetate (24b):** 89 mg, 98%, glass, ^1^H NMR (400 MHz, CD_3_OD): *δ* 4.38 (2H, s, CH_2_), 7.20 (2H, d, *J* = 8.4 Hz, 2 × ArH), 7.27 (2H, d, *J* = 8.4 Hz, 2 × ArH), 7.55 (2H, d, *J* = 8.4 Hz, 2 × ArH), and 7.80 (2H, d, *J* = 8.4 Hz, 2 × ArH). HRMS (ESI): Calcd. for C_14_H_16_ClN_4_O_2_S (M + H)^+^ 339.0677 and found 339.0686.

**Amino((4-((2,3-dichlorophenyl)sulfonamido)benzyl)amino)methaniminium 2,2,2-trifluoroacetate (24c):** 97 mg, >99%, glass. ^1^H NMR (400 MHz, CD_3_OD): *δ* 4.34 (2H, s, CH_2_), 7.21–7.28 (4H, m, 4 × ArH), 7.46 (1H, t, *J* = 8.0 Hz, ArH), 7.81 (1H, dd, *J* = 8.0, 1.6 Hz, ArH), and 8.10 (1H, dd, *J* = 8.0, 1.6 Hz, ArH). HRMS (ESI): Calcd. for C_14_H_15_Cl_2_N_4_O_2_S (M + H)^+^ 373.0287 and found 373.0296.

**Amino((4-((3-trifluoromethyl)phenyl)sulfonamido)benzyl)amino) methaniminium 2,2,2-trifluoroacetate (24d):** 95 mg, 97%, glass. ^1^H NMR (400 MHz, CD_3_OD): *δ* 4.38 (2H, s, CH_2_), 7.20 (2H, d, *J* = 8.8 Hz, 2 × ArH), 7.29 (2H, d, *J* = 8.4 Hz, 2 × ArH), 7.76 (1H, t, *J* = 8.2 Hz, ArH), 7.95 (1H, d, *J* = 8.0 Hz, ArH), and 8.05–8.11 (2H, m, 2 × ArH). HRMS (ESI): Calcd. for C_15_H_16_F_3_N_4_O_2_S (M + H)^+^ 373.0941 and found 373.0945.

**General procedure: Synthesis of amino((benzamidobenzyl)amino) methaniminium 2,2,2-trifluoroacetate derivatives (20e**, **24e):** Compound **19e** or **23e** (0.1 mmol) was dissolved in CH_2_Cl_2_ (0.8 mL) and then TFA (0.2 mL) was added. The reaction mixture was stirred for 2 h at 0 °C. The mixture was concentrated to dryness to give **20a** or **24e**.

**Amino((3-benzamidobenzyl)amino)methaniminium 2,2,2-trifluoroacetate (20e):** 38 mg, 99%, pale yellow glass. ^1^H NMR (400 MHz, DMSO-*d_6_*): *δ* 4.44 (2H, s, CH_2_), 7.10 (1H, d, *J* = 8.0 Hz, ArH), 7.42 (1H, t, *J* = 8.0 Hz, ArH), 7.56–7.62 (3H, m, 3 × ArH), 7.65 (1H, d, *J* = 7.2 Hz, ArH), 7.90 (1H, s, ArH), 8.00 (2H, d, *J* = 7.2 Hz, 2 × ArH), and 8.01 (1H, s, NH). HRMS (ESI): Calcd. for C_15_H_17_N_4_O (M + H)^+^ 269.1397 and found: 269.1401.

**Amino((4-benzamidobenzyl)amino)methaniminium 2,2,2-trifluoroacetate (24e):** 38 mg, 99%, pale yellow glass. ^1^H NMR (400 MHz, DMSO-*d_6_*): *δ* 4.39 (2H, s, CH_2_), 7.35 (2H, d, *J* = 8.4 Hz, 2 × ArH), 7.59 (2H, t, *J* = 7.2 Hz, 2 × ArH), 7.66 (1H, t, *J* = 7.2 Hz, ArH), 7.84 (2H, d, *J* = 8.4 Hz, 2 × ArH), 8.00 (2H, d, *J* = 8.0 Hz, 2 × ArH), and 8.01 (1H, s, NH). HRMS (ESI): Calcd. for C_15_H_17_N_4_O (M + H)^+^ 269.1397 and found: 269.1402.

***tert*-Butyl 7-hydroxy-1,2,3,4-dihydroisoquinoline-2-carboxylate (26a):** In a solution of **25a** (349 mg, 2.34 mmol) in THF/water (5 mL/1 mL), Boc_2_O (545 mg, 2.5 mmol) and Et_3_N (0.4 mL, 2.8 mmol) were added. The mixture was stirred at r.t. for 16 h and partitioned between EtOAc (50 mL) and water (50 mL). The organic layer was washed with brine, dried (MgSO_4_), and concentrated in vacuo. Purification by flash column chromatography (petrol ether/EtOAc 7:3) gave a white solid (475 mg, 81% yield), m.p. 130–131 °C. ^1^H NMR (400 MHz, CDCl_3_): *δ* 1.49 (9H, s, *t*-Bu), 2.74 (2H, t, *J* = 5.9 Hz, CH_2_), 3.62 (2H, t, *J* = 6.0 Hz, CH_2_), 4.51 (2H, s, CH_2_), 6.62–6.65 (2H, m, 2 × ArH), and 6.98 (1H, d, *J* = 8.2 Hz, ArH).

***tert*-Butyl 7-(2,3-dichlorobenzyloxy)-1,2,3,4-tetrahydroisoquinoline-2-carboxylate (27a):** In a solution of **26a** (370 mg, 1.48 mmol) in acetone (15 mL), 2,3-dichlorobenzyl bromide (437 mg, 1.6 mmol) was added, followed by K_2_CO_3_ (262 mg, 1.9 mmol). The mixture was stirred at r.t. overnight and partitioned between EtOAc (30 mL) and water (30 mL). The organic layer was washed with brine, dried (MgSO_4_), and concentrated *in vacuo.* Purification by flash column chromatography (petrol ether to petrol ether/EtOAc 3:1) afforded **27a** as clear oil (500 mg, 83%). ^1^H NMR (400 MHz, CDCl_3_): *δ* 1.48 (9H, s, *t*-Bu), 2.76 (2H, t, *J* = 5.7 Hz, CH_2_), 3.62 (2H, t, *J* = 5.7 Hz, CH_2_), 4.52 (2H, s), 5.15 (2H, s), 6.75–6.79 (2H, m, 2 × ArH), 7.05 (1H, d, *J* = 8.4 Hz, ArH), 7.23 (1H, t, *J* = 7.9 Hz, ArH), and 7.45 (2H, m, 2 × ArH). HRMS (ESI): Calcd. for C_21_H_23_Cl_2_NNaO_3_ (M + Na)^+^ 430.0953 and found 430.0970.

***tert*-Butyl *N*-[{[(*tert*-butoxy)carbonyl]imino}({7-(2,3-dichlorobenzyloxy)-1,2,3,4-tetrahydroisoquinolin-2-yl})methyl]carbamate (28a):** In a solution of 27a (450 mg, 1.1 mmol) in CH_2_Cl_2_ (63 mL), TFA (2 mL) was added. The mixture was shaken at r.t. for 10 h and evaporated in vacuo to give to a yellow residue. The crude product was dissolved in DMF (5 mL) and Et_3_N (0.6 mL). *S*-Methyl-*N*,*N*’-bis(*tert*-butoxycarbonyl)isothiourea (390 mg, 1.32 mmol) was added, followed by HgCl_2_ (200 mg, 2.37 mmol). The mixture was stirred at r.t. for 16 h, diluted with EtOAc (50 mL), and filtered through Celite. The organic layer was washed with brine, dried (MgSO_4_), and concentrated *in vacuo.* Purification by flash column chromatography (petrol ether to petrol ether/EtOAc 3:2) afforded **28a** as a foamy powder solid (340 mg, 56% yield), m.p. 59–61 °C. ^1^H NMR (400 MHz, CDCl_3_): *δ* 1.50 (18H, s, 2 × *t*-Bu), 2.98 (2H, t, *J* = 5.7 Hz, CH_2_), 3.80 (2H, t, *J* = 5.7 Hz, CH_2_), 4.75 (2H, s), 5.12 (2H, s), 6.70 (1H, br.s, ArH), 6.81 (1H, dd, *J* = 8.0, 1.9 Hz, ArH), 7.05 (1H, d, *J* = 8.0 Hz, ArH), 7.22 (1H, d, *J* = 8.0 Hz, ArH), 7.40–7.46 (2H m, 2 × ArH), and 10.2 (1H, s, NH). HRMS (ESI): Calcd. for C_27_H_34_Cl_2_N_3_O_5_ (M + H)^+^ 550.1876 and found 550.1876.

**7-(2,3-Dichlorobenzyloxy)-1,2,3,4-tetrahydroisoquinoline-2-carboximidamide 2,2,2-trifluoroacetate (29a):** The compound was synthesised as described for **28a**. A white solid (30 mg, 82%) was obtained. ^1^H NMR (400 MHz, DMSO-*d_6_*): *δ* 2.89 (2H, t, *J* = 5.5 Hz, CH_2_), 3.50 (2H, t, *J* = 5.2 Hz, CH_2_), 4.51 (2H, s), 5.20 (2H, s), 6.85 (1H, d, *J* = 1.7 Hz, ArH), 6.95 (1H, dd, *J* = 7.8, 1.5 Hz, ArH), 7.25 (1H, d, *J* = 7.7 Hz, ArH), 7.45 (1H, t, *J* = 7.9 Hz, ArH), 7.60 (1H, d, *J* = 8.0 Hz, ArH), and 7.70 (1H, d, *J* = 8.0 Hz, ArH). HRMS (ESI): Calcd. for C_17_H_18_Cl_2_N_3_O (M + H)^+^ 350.0827 and found 350.0821.

***tert*-Butyl 5-hydroxy-1,2,3,4-tetrahydroisoquinoline-2-carboxylate (26b):** A solution of **25b** (1.0 g, 90% purity, 6.2 mmol) in AcOH (20 mL) was reacted over H_2_ (1 atm) and PtO_2_ (85 mg) at r.t. for 48 h. The reaction mixture was then filtered through Celite and concentrated *in vacuo.* The residue was dissolved in acetone (3 mL) and diluted with Et_2_O (3 mL). The precipitate was collected and dried *in vacuo.* The crude product (700 mg, 4.7 mmol) was suspended in THF/water (10 mL/2 mL). Boc_2_O (1.1 g, 5.0 mmol) and Et_3_N (1.5 mL, 10 mmol) were added. The mixture was stirred at r.t. for 16 h and partitioned between EtOAc (50 mL) and water (50 mL). The organic layer was washed with brine, dried (MgSO_4_), and concentrated *in vacuo.* Purification by flash column chromatography (petrol ether/EtOAc 7:3) gave **26b** as a white solid (490 mg, 42% yield), m.p. 156–158 °C. ^1^H NMR (400 MHz, CDCl_3_): *δ* 1.48 (s, 9H, *t*-Bu), 2.75 (2H, t, *J* = 6.0 Hz, CH_2_), 3.65 (2H, t, *J* = 6.1 Hz, CH_2_), 4.55 (2H, s, CH_2_), 6.63 (1H, d, *J* = 7.8 Hz, ArH), 6.68 (1H, d, *J* = 7.9 Hz, ArH), and 7.03 (1H, d, *J* = 7.8 Hz, ArH). HRMS (ESI): Calcd. for C_14_H_19_NNaO_3_ (M + Na)^+^ 272.1263 and found 272.1244.

***tert*-Butyl 5-(2,3-dichlorobenzyloxy)-1,2,3,4-tetrahydroisoquinoline-2-carboxylate (27b):** The compound was synthesised as described for **27a**. A white solid (480 mg, 79%) was obtained, m.p. 138–139 °C. ^1^H NMR (400 MHz, CDCl_3_): *δ* 1.49 (9H, s, *t*-Bu), 2.86 (2H, t, *J* = 5.8 Hz, CH_2_), 3.67 (2H, t, *J* = 5.9 Hz, CH_2_), 4.58 (2H, s), 5.16 (2H, s), 6.74–6.77 (2H, m, 2 × ArH), 7.14 (1H, t, *J* = 8.2 Hz, ArH), 7.22–7.26 (2H, m, 2 × ArH), 7.44 (1H, d, *J* = 8.7 Hz, ArH), and 7.49 (1H, d, *J* = 9.0 Hz, ArH). HRMS (ESI): Calcd. for C_21_H_23_Cl_2_NNaO_3_ (M + Na)^+^ 430.0953 and found 430.0917.

***tert*-Butyl *N*-[{[(*tert*-butoxy)carbonyl]amino}({5-(2,3-dichlorobenzyloxy)-1,2,3,4-tetrahydroisoquinolin-2-yl})methylidene]carbamate (28b):** The compound was synthesised as described for **28a**. A white solid (280 mg, 59%) was obtained, m.p. 129–130 °C. ^1^H NMR (400 MHz, CDCl_3_): *δ* 1.47 (9H, s, *t*-Bu), 1.51 (9H, s, *t*-Bu), 2.91 (2H, t, *J* = 5.5 Hz, CH_2_), 3.71 (2H, t, *J* = 5.5 Hz, CH_2_), 4.62 (2H, s), 5.21 (2H, s), 6.79–6.82 (2H, m, 2 × ArH), 7.18 (1H, t, *J* = 8.3 Hz, ArH)), 7.31–7.34 (2H, m, 2 × ArH), 7.46 (1H, d, *J* = 8.5 Hz, ArH), and 7.52 (1H, d, *J* = 8.3 Hz, ArH). HRMS (ESI): Calcd. for C_27_H_34_Cl_2_N_3_O_5_ (M + H)^+^ 550.1876 and found 550.1882.

**5-(2,3-Dichlorobenzyloxy)-1,2,3,4-tetrahydroisoquinoline-2-carboximidamide hydrochloride (29b):** The compound in TFA salt form (100 mg) was synthesised as described for **28a**. The TFA salt was converted to hydrochloride **29b** using HCl (0.5M in MeOH). ^1^H NMR (400 MHz, DMSO-*d_6_*): *δ* 2.85 (2H, t, *J* = 5.5 Hz, CH_2_), 3.52 (2H, t, *J* = 5.2 Hz, CH_2_), 4.60 (2H, s), 5.35 (2H, s), 6.82 (1H, d, *J* = 7.8 Hz, ArH), 7.05 (1H, d, *J* = 7.9 Hz, ArH), 7.28 (1H, t, *J* = 8.0 Hz, ArH), 7.47–7.51 (1H, m, ArH), 7.65 (1H, d, *J* = 8.3 Hz, ArH), and 7.73 (1H, d, *J* = 8.5 Hz, ArH). HRMS (ESI): Calcd. for C_17_H_18_Cl_2_N_3_O (M + H)^+^ 350.0827 and found 350.0899.

***tert*-Butyl *N*-[(7-bromo-1,2,3,4-tetrahydroisoquinolin-2-yl)({[(*tert*-butoxy) carbon yl]amino})methylidene]carbamate** (**31**): In a solution of 7-bromo-1,2,3,4-tetrahydroisoquinoline **30** (318 mg, 1.5 mmol) in DMF (5 mL), *S*-methyl-*N*,*N*’-bis(*tert*-butoxycarbonyl)isothiourea (436 mg, 1.5 mmol) was added, followed by Et_3_N (0.5 mL). The mixture was stirred at r.t. for 4 h and partitioned between EtOAc (100 mL) and citric acid (50 mL, 5% in water). The organic layer was washed with brine, dried (MgSO_4_), filtered, and concentrated *in vacuo.* Purification by flash column chromatography (petrol ether to petrol ether/EtOAc 3:1) afforded **31** as a white solid (500 mg, 73% yield), m.p. 131–132 °C. ^1^H NMR (400 MHz, CDCl_3_): *δ* 1.50 (18H, s, 2 × *t*-Bu), 2.90 (2H, t, *J* = 5.5 Hz, CH_2_), 3.87 (2H, t, *J* = 5.7 Hz, CH_2_), 4.70 (2H, s, CH_2_), 7.01 (1H, d, *J* = 8.0 Hz, ArH), 7.23 (1H, br.s, ArH), 7.28 (1H, dd, *J* = 8.2, 1.9 Hz, ArH), and 10.3 (1H, s, NH). HRMS (ESI): Calcd. for C_20_H_29_BrN_3_O_4_ (M + H)^+^ 454.1341 and found 454.1356.

***tert*-Butyl *N*-[7-(4-*tert*-butylphenyl)-1,2,3,4-tetrahydroisoquinoline-2-carboximido yl]carbamate** (**32**): In a solution of **31** (400 mg, 0.88 mmol) in dioxane (6 mL) and water (2 mL), 4-*tert*-butylphenylboronic acid (188 mg, 1.05 mmol) and K_2_CO_3_ (242 mg, 1.76 mmol) were added. The mixture was degassed under vacuum for 1 min and Pd(PPh_3_)_4_ (20 mg) was added. The reaction mixture was stirred at 100 °C under N_2_ for 4 h. After cooling to r.t., the mixture was partitioned between EtOAc (50 mL) and water (50 mL). The organic layer was washed with brine, dried (MgSO_4_), filtered, and concentrated *in vacuo.* Purification by flash column chromatography (CH_2_Cl_2_/MeOH (9:1) gave **32** as a clear oil (190 mg, 53% yield). ^1^H NMR (400 MHz, CDCl_3_): *δ* 1.35 (9H, s, *t*-Bu), 1.51 (9H, s, *t*-Bu), 2.90 (2H, t, *J* = 5.3 Hz, CH_2_), 3.75 (2H, t, *J* = 5.2 Hz, CH_2_), 4.73 (2H, s, CH_2_), 7.18 (1H, d, *J* = 8.3 Hz, ArH), 7.35 (1H, br.s, ArH), 7.41 (1H, dd, *J* = 8.1, 1.5 Hz, ArH), 7.46–7.50 (4H, m, 4 × ArH), and 10.1 (1H, s, NH). HRMS (ESI): Calcd. for C_25_H_34_N_3_O_2_ (M + H)^+^ 408.2651 and found 408.2687.

***N*-[7-(4-*tert*-Butylphenyl)-1,2,3,4-tetrahydroisoquinoline-2-carboximidoyl]cabam-ate 2,2,2-trifluoroacetate (33):** The compound was synthesised as described for **28a**. A white solid (35 mg, 77%) was obtained, m.p. 218–219 °C. ^1^H NMR (400 MHz, CD_3_OD): *δ* 1.35 (9H, s, *t*-Bu), 3.00 (2H, t, *J* = 5.5 Hz, CH_2_), 3.70 (2H, t, *J* = 5.5 Hz, CH_2_), 4.70 (2H, s, CH_2_), 7.39 (1H, d, *J* = 8.2 Hz, ArH), 7.46 (1H, d, *J* = 1.6 Hz, ArH), and 7.50–7.65 (5H, m, 5 × ArH). HRMS (ESI): Calcd. for C_20_H_26_N_3_ (M + H)^+^ 308.2127 and found 308.2131.

**General Procedure: Guanylation of *O*-benzylhydroxylamines (34a–c):** In a solution of the substituted amine (1.5 mmol) in DMF (5 mL), *S*-methyl-*N*,*N*’-bis(*tert*-butoxycarbonyl)isothiourea (285 mg, 0.98 mmol) was added, followed by Et_3_N (0.6 mL). The mixture was stirred at r.t. overnight and then partitioned between EtOAc (100) ml and brine (50 mL). The organic layer was washed with brine, dried (MgSO_4_), and concentrated in vacuo. Purification by flash column chromatography (petrol ether to petrol ether/EtOAc 11:9) afforded **35a–c**.

***tert*-Butyl *N*-[{[(*tert*-butoxy)carbonyl]amino}({[(3-phenylphenyl)methoxy]amino}) methylidene]carbamate** (**35a**): A foamy powder (190 mg, 69%) was obtained. ^1^H NMR (400 MHz, CDCl_3_): *δ* 1.46 (9H, s, *t*-Bu), 1.49 (9H, s, *t*-Bu), 5.13 (2H, s, CH_2_), 7.33–7.46 (5H, m, 5 × ArH), 7.56–7.63 (3H, m, 3 × ArH), 7.64–7.68 (1H, m, ArH), 7.73 (s, 1H, NH), and 9.16 (s, 1H, NH).

***tert*-Butyl *N*-[{[(*tert*-butoxy)carbonyl]amino}({[(3-phenoxyphenyl)methoxy] amino})methylidene]carbamate** (**35b**): A foamy powder (185 mg, 65%) was obtained. ^1^H NMR (400 MHz, CDCl_3_): *δ* 1.36 (18H, s, 2 × *t*-Bu), 5.28 (2H, s, CH_2_), 7.41–7.55 (5H, m, 4 × ArH, NH), 7.59 (1H, dt, *J* = 8.1, 1.5 Hz, ArH), 7.69–7.74 (2H, m, 2 × ArH), 7.76 (1H, s, NH), 7.81 (1H, d, *J* = 8.1 Hz, ArH), and 7.82 (1H, d, *J* = 8.2 Hz, ArH).

***tert*-Butyl *N*-[{[(*tert*-butoxy)carbonyl]amino}({[3-(4-tert-butylphenyl)phenyl]meth- oxy}amino)methylidene]carbamate** (**35c**): A foamy powder (200 mg, 80%) was obtained. ^1^H NMR (400 MHz, CDCl_3_): *δ* 1.36 (9H, s, *t*-Bu), 1.49 (9H, s, *t*-Bu), 1.50 (9H, s, *t*-Bu), 5.12 (2H, s, CH_2_), 7.38–7.45 (4H, m, 4 × ArH), 7.52–7.58 (3H, m, 3 × ArH), 7.62–7.66 (1H, m, ArH), 7.72 (br.s, 1H, NH), and 9.18 (1H, s, NH).

**General Procedure: Synthesis of benzyloxy guanidine derivatives (36a–c):** In a solution of the substituted *N,N*′-di-Boc-guanidino derivative (**35a–c**) (0.3 mmol) in CH_2_Cl_2_ (2 mL), TFA (1 mL) was added. The mixture was shaken at r.t. overnight and then evaporated to dryness. Et_2_O (1 mL) was added, and the precipitate was collected, washed with diethyl ether, and dried in vacuo to give **36a–c** as a white or off-white solid.

**1-[(3-Phenylphenyl)methoxy]guanidinium 2,2,2-trifluoroacetate (36a):** An off-white solid (65 mg, 92%) was obtained. ^1^H NMR (400 MHz, DMSO-*d_6_*): *δ* 5.00 (2H, s, CH_2_), 7.43–7.57 (4H, m, 4 × ArH), 7.72 (1H, t, *J* = 1.9 Hz, ArH), 7.70–7.76 (4H, m, 4 × ArH), 7.83 (1H, br.s, NH), and 11.2 (1H, s, NH). HRMS (ESI): Calcd. for C_14_H_16_N_3_O (M + H)^+^ 242.1293 and found 242.1282.

**1-[(3-Phenoxyphenyl)methoxy]guanidinium 2,2,2-trifluoroacetate (36b):** A white solid (95 mg, 97%) was obtained. ^1^H NMR (400 MHz, CDCl_3_): *δ* 4.76 (2H, s, CH_2_), 6.96–7.05 (4H, m, 4 × ArH), 7.05 (1H, dt, *J* = 8.5, 1.9 Hz, ArH), 7.12 (1H, td, *J* = 8.9, 2.1 Hz, ArH), 7.30–7.35 (3H, m, 3 × ArH), and 10.9 (1H, s, NH). HRMS (ESI): Calcd. for C_14_H_15_N_3_NaO_2_ (M + Na)^+^ 280.1062 and found 280.1066.

**1-{[3-(4-*tert*-Butylphenyl)phenyl]methoxy}guanidinium 2,2,2-trifluoroacetate (36c):** A white solid (75 mg, 77%) was obtained. ^1^H NMR (400 MHz, CD_3_OD): *δ* 1.43 (9H, s, *t*-Bu), 5.12 (2H, s, CH_2_), 7.46 (1H, dt, *J* = 8.9, 1.7 Hz, ArH), 7.52–7.56 (3H, m, 3 × ArH), 7.63–7.66 (2H, m, 2 × ArH), 7.72 (1H, dt, *J* = 8.8, 2.0 Hz, ArH), and 7.76–7.79 (1H, m, ArH). HRMS (ESI): Calcd. for C_18_H_24_N_3_O (M + H)^+^ 298.1919 and found 298.1938.

**General Procedure: Synthesis of 3-benzyloxybenzaldehyde derivatives (38a–t):** The 3-hydroxybenzaldehyde derivative **37a–c** (2 mmol) and K_2_CO_3_ (4 mmol) were placed in a round-bottom flask. DMF (3 mL) was added. Then, the corresponding benzyl halide (2.2 mmol) was added and the reaction mixture was stirred at r.t. for 18 h. Water (125 mL) and brine (25 mL) were added, and the mixture was extracted with Et_2_O (2 × 100 mL) or CH_2_Cl_2_ (for **38q**; 2 × 100 mL). The combined organic layers were dried (NaCl), filtered, and concentrated *in vacuo.* Crystallisation from Et_2_O or pentane/Et_2_O gave the corresponding aldehyde derivatives **38a–c** and **38h–t**. Aldehyde derivatives **38d–g** were purified by flash column chromatography.

**3-(2,3-Dichlorobenzyloxy)-benzaldehyde (38a):** 445 mg, 79%, white solid. ^1^H NMR (400 MHz, CDCl_3_): *δ* 5.22 (2H, s, CH_2_), 7.21–7.28 (3H, m, 3 × ArH), 7.42–7.55 (5H, m, 5 × ArH), and 9.99 (1H, s, CH=O). ^13^C NMR (100 MHz, CDCl_3_): *δ* 67.8, 113.5, 122.1, 124.2, 126.8, 127.6, 130.0, 130.4, 130.9, 133.4, 136.6, 138.1, 159.0, and 192.1. HRMS (ESI): Calcd. for C_14_H_11_Cl_2_O_2_ (M + H)^+^ 281.0131 and found 281.0142.

**3-(2-Chloro-3-(trifluoromethyl)benzyloxy)-benzaldehyde (38b):** 474 mg, 75%, white solid. ^1^H NMR (400 MHz, CDCl_3_): *δ* 5.27 (2H, s, CH_2_), 7.24–7.29 (1H, m, ArH), 7.42 (1H, t, *J* = 8.0 Hz, ArH), 7.46–7.54 (3H, m, 3 × ArH), 7.70 (1H, d, *J* = 7.6 Hz, ArH), 7.79 (1H, d, *J* = 7.6 Hz, ArH), and 9.99 (1H, s, CH=O). HRMS (ESI): Calcd. for C_15_H_11_ClF_3_O_2_ (M + H)^+^ 315.0394 and found 315.0403.

**3-(4-Chlorobenzyloxy)-benzaldehyde (38c):** 430 mg, 87%, white solid, m.p. 52–54 °C. ^1^H NMR (400 MHz, CDCl_3_): *δ* 5.08 (2H, s, CH_2_), 7.23 (1H, dt, *J* = 7.6, 2.0 Hz, ArH), 7.34–7.40 (4H, m, 4 × ArH), 7.43–7.51 (3H, m, 3 × ArH), and 9.97 (1H, s, CH=O). HRMS (ESI): Calcd. for C_14_H_12_ClO_2_ (M + H)^+^ 247.0520 and found 247.0531.

**3-(4-(Trifluoromethyl)benzyloxy)-benzaldehyde (38d):** Purification by flash column chromatography (petrol ether to petrol ether/EtOAc 9:1) gave **38d** (501 mg, 89%, colourless oil). ^1^H NMR (400 MHz, CDCl_3_): *δ* 5.18 (2H, s, CH_2_), 7.23–7.28 (1H, m, ArH), 7.44–7.52 (3H, m, 3 × ArH), 7.56 (2H, d, *J* = 8.0 Hz, 2 × ArH), 7.66 (2H, d, *J* = 8.0 Hz, 2 × ArH), and 9.97 (1H, d, *J* = 1.2 Hz, CH=O). HRMS (ESI): Calcd. for C_15_H_12_F_3_O_2_ (M + H)^+^ 281.0784 and found 281.0789.

**3-(3-Chlorobenzyloxy)-benzaldehyde (38e):** Purification by flash column chromatography (petrol ether to petrol ether/EtOAc 9:1) gave **38e** (424 mg, 86%, colourless oil). ^1^H NMR (400 MHz, CDCl_3_): *δ* 5.09 (2H, s, CH_2_), 7.22–7.26 (1H, m, ArH), 7.29–7.33 (3H, m, 3 × ArH), 7.44–7.51 (4H, m, 4 × ArH), and 9.97 (1H, s, CH=O). HRMS (ESI): Calcd. for C_14_H_12_ClO_2_ (M + H)^+^ 247.0520 and found 247.0525.

**3-(3-(Trifluoromethyl)benzyloxy)-benzaldehyde (38f):** Purification by flash column chromatography (petrol ether to petrol ether/EtOAc 9:1) gave **38f** (478 mg, 85%, colourless oil). ^1^H NMR (400 MHz, CDCl_3_): *δ* 5.17 (2H, s, CH_2_), 7.24–7.28 (1H, m, ArH), 7.45–7.51 (3H, m, 3 × ArH), 7.53 (1H, d, *J* = 8.0 Hz, ArH), 7.61 (1H, d, *J* = 8.8 Hz, ArH), 7.63 (1H, d, *J* = 8.0 Hz, ArH), 7.72 (1H, s, ArH), and 9.98 (1H, s, CH=O). HRMS (ESI) calcd. for C_15_H_12_F_3_O_2_^+^ (M + H)^+^ 281.0784 and found 281.0790.

**3-(2,3-Dichlorobenzyloxy)-4-methoxybenzaldehyde (38g):** Purification by flash column chromatography (CH_2_Cl_2_ to CH_2_Cl_2_/EtOAc 9:1) gave **38g** (436 mg, 87%). m.p. 117–119 °C. ^1^H NMR (400 MHz, CDCl_3_): *δ* 3.98 (3H, s, OCH_3_), 5.28 (2H, s, CH_2_), 7.08 (1H, d,* J* = 8.3 Hz, ArH), 7.23 (1H, d,* J* = 8.1 Hz, ArH), 7.43 (2H, d, *J* = 8.2 Hz, 2 × ArH), 7.52 (2H, dd, *J* = 8.0, 1.1 Hz, 2 × ArH), and 9.88 (1H, s, CH=O). HRMS (ESI): Calcd. for C_15_H_13_Cl_2_O_3_ (M + H)^+^ 311.0242 and found 311.0237.

**2-Chloro-3-(2-chloro-3-methoxybenzyloxy)-benzaldehyde (38h):** 597 mg, 96%, white solid, m.p. 124–126 °C. ^1^H NMR (400 MHz, CDCl_3_): *δ* 3.92 (3H, s, OCH_3_), 5.28 (2H, s, CH_2_), 6.92 (1H, dd, *J* = 7.0, 2.6 Hz, ArH), 7.19 (1H, dd, *J* = 8.0, 1.2 Hz, ArH), 7.24–7.33 (3H, m, 3 × ArH), 7.54 (1H, dd, *J* = 7.8, 1.4 Hz, ArH), and 10.53 (1H, d, *J* = 0.4 Hz, CH=O). HRMS (ESI): Calcd. for C_15_H_13_Cl_2_O_3_ (M + H)^+^ 311.0236 and found 311.0232.

**2-Chloro-3-benzyloxybenzaldehyde (38i):** 425 mg, 86%, white solid, m.p. 103–105 °C. ^1^H NMR (400 MHz, CDCl_3_): *δ* 5.20 (2H, s, CH_2_), 7.19 (1H, dd, *J* = 8.0, 1.2 Hz, ArH), 7.29 (1H, t, *J* = 7.8 Hz, ArH), 7.35 (1H, d, *J* = 6.8 Hz, ArH), 7.41 (2H, t, *J* = 7.4 Hz, 2 × ArH), 7.47 (2H, d, *J* = 7.2 Hz, 2 × ArH), 7.53 (1H, dd, *J* = 7.8, 1.4 Hz, ArH), and 10.54 (1H, s, CH=O). HRMS (ESI): Calcd. for C_14_H_12_ClO_2_ (M + H)^+^ 247.0520 and found 247.0528.

**2-Chloro-3-(4-chlorobenzyloxy)-benzaldehyde (38j):** 522 mg, 93%, white solid, m.p. 94–96 °C. ^1^H NMR (400 MHz, CDCl_3_): *δ* 5.15 (2H, s, CH_2_), 7.16 (1H, dd, *J* = 8.0, 1.6 Hz, ArH), 7.30 (1H, t, *J* = 8.0 Hz, ArH), 7.35–7.43 (4H, m, 4 × ArH), 7.54 (1H, dd, *J* = 7.8, 1.4 Hz, ArH), and 10.53 (1H, d, *J* = 0.8 Hz, CH=O). HRMS (ESI): Calcd. for C_14_H_11_Cl_2_O_2_ (M + H)^+^ 281.0131 and found 281.0127.

**2-Chloro-3-(2,3-dichlorobenzyloxy)-benzaldehyde (38k):** 600 mg, 95%, white solid, m.p. 150–152 °C. ^1^H NMR (400 MHz, CDCl_3_): *δ* 5.27 (2H, s, CH_2_), 7.20 (1H, d, *J* = 8.0 Hz, ArH), 7.28 (1H, t, *J* = 8.0 Hz, ArH), 7.34 (1H, t, *J* = 8.0 Hz, ArH), 7.46 (1H, d, *J* = 8.0 Hz, ArH), 7.57 (1H, d, *J* = 8.0 Hz, ArH), 7.61 (1H, d, *J* = 8.0 Hz, ArH), and 10.54 (1H, s, CH=O). HRMS (ESI): Calcd. for C_14_H_10_Cl_3_O_2_ (M + H)^+^ 314.9741 and found 314.9748.

**2-Chloro-3-(2-chloro-3-(trifluoromethyl)benzyloxy)-benzaldehyde (38m):** 632 mg, 90%, white solid, m.p. 128–131 °C. ^1^H NMR (400 MHz, CDCl_3_): *δ* 5.31 (2H, s, CH_2_), 7.23 (1H, dd, *J* = 8.2, 1.4 Hz, ArH), 7.36 (1H, t, *J* = 8.0 Hz, ArH), 7.47 (1H, t, *J* = 7.8 Hz, ArH), 7.59 (1H, dd, *J* = 7.8, 1.4 Hz, ArH), 7.71 (1H, d, *J* = 8.0 Hz, ArH), 7.94 (1H, d, *J* = 7.6 Hz, ArH), and 10.55 (1H, d, *J* = 0.8 Hz, CH=O). HRMS (ESI): Calcd. for C_15_H_10_Cl_2_F_3_O_2_ (M + H)^+^ 349.0004 and found 349.0011.

**2-Chloro-3-(2,4-dichlorobenzyloxy)-benzaldehyde (38n):** 580 mg, 92%, white solid, m.p. 115–116 °C. ^1^H NMR (400 MHz, CDCl_3_): *δ* 5.22 (2H, s, CH_2_), 7.20 (1H, dd, *J* = 8.2, 1.4 Hz, ArH), 7.30–7.36 (2H, m, 2 × ArH), 7.43 (1H, d, *J* = 2.4 Hz, ArH), 7.57 (1H, dd, *J* = 7.8, 1.4 Hz, ArH), 7.62 (1H, d, *J* = 8.4 Hz, ArH), and 10.54 (1H, d, *J* = 0.8 Hz, CH=O). HRMS (ESI): Calcd. for C_14_H_10_Cl_3_O_2_ (M + H)^+^ 314.9741 and found 314.9748.

**2-Chloro-3-(2,5-dichlorobenzyloxy)-benzaldehyde (38o):** 535 mg, 85%, white solid, m.p. 132–134 °C. ^1^H NMR (400 MHz, CDCl_3_): *δ* 5.22 (2H, s, CH_2_), 7.21 (1H, dd, *J* = 8.4, 1.2 Hz, ArH), 7.27 (1H, dd, *J* = 8.4, 2.4 Hz, ArH), 7.34 (1H, d, *J* = 8.8 Hz, ArH), 7.35 (1H, t, *J* = 8.0 Hz, ArH), 7.58 (1H, dd, *J* = 8.0, 1.6 Hz, ArH), 7.69 (1H, d, *J* = 2.4 Hz, ArH), and 10.55 (1H, s, CH=O). HRMS (ESI): Calcd. for C_14_H_10_Cl_3_O_2_ (M + H)^+^ 314.9741 and found 314.9745.

**2-Chloro-3-(3,4-dichlorobenzyloxy)-benzaldehyde (38p):** 610 mg, 97%, white solid, m.p. 140–142 °C. ^1^H NMR (400 MHz, CDCl_3_): *δ* 5.13 (2H, s, CH_2_), 7.15 (1H, dd, *J* = 8.2, 1.4 Hz, ArH), 7.29–7.34 (2H, m, 2 × ArH), 7.48 (1H, d, *J* = 8.4 Hz, ArH), 7.56 (1H, dd, *J* = 8.0, 1.2 Hz, ArH), 7.58 (1H, s, ArH), and 10.53 (1H, s, CH=O). HRMS (ESI): Calcd. for C_14_H_10_Cl_3_O_2_ (M + H)^+^ 314.9741 and found 314.9744.

**2-Chloro-3-(2,3,5-trichlorobenzyloxy)-benzaldehyde (38q):** 610 mg, 87%, white solid, m.p. 158–161 °C. ^1^H NMR (400 MHz, CDCl_3_): *δ* 5.22 (2H, s, CH_2_), 7.20 (1H, dd, *J* = 8.2, 1.4 Hz, ArH), 7.36 (1H, t, *J* = 8.0 Hz, ArH), 7.48 (1H, d, *J* = 2.4 Hz, ArH), 7.60 (1H, dd, *J* = 8.0, 1.2 Hz, ArH), 7.64 (1H, d, *J* = 2.4 Hz, ArH), and 10.55 (1H, d, *J* = 0.4 Hz, CH=O). HRMS (ESI): Calcd. for C_14_H_9_Cl_4_O_2_ (M + H)^+^ 348.9351 and found 348.9365.

**2-Chloro-3-(3-(trifluoromethyl)benzyloxy)-benzaldehyde (38r):** 617 mg, 98%, white solid, m.p. 83–85 °C. ^1^H NMR (400 MHz, CDCl_3_): *δ* 5.23 (2H, s, CH_2_), 7.19 (1H, dd, *J* = 8.0, 1.2 Hz, ArH), 7.33 (1H, t, *J* = 8.0 Hz, ArH), 7.54 (1H, t, *J* = 7.8 Hz, ArH), 7.57 (1H, dd, *J* = 7.8, 1.4 Hz, ArH), 7.62 (1H, d, *J* = 8.0 Hz, ArH), 7.69 (1H, d, *J* = 7.6 Hz, ArH), 7.74 (1H, s, ArH), and 10.54 (1H, d, *J* = 0.4 Hz, CH=O). HRMS (ESI): Calcd. for C_15_H_11_ClF_3_O_2_ (M + H)^+^ 315.0394 and found 315.0401.

**2-Chloro-3-(4-(trifluoromethyl)benzyloxy)-benzaldehyde (38s):** 605 mg, 96%, white solid, m.p. 82–84 °C. ^1^H NMR (400 MHz, CDCl_3_): *δ* 5.25 (2H, s, CH_2_), 7.18 (1H, dd, *J* = 7.8, 1.4 Hz, ArH), 7.32 (1H, dt, *J* = 8.0, 0.8 Hz, ArH), 7.56 (1H, dd, *J* = 7.8, 1.4 Hz, ArH), 7.60 (2H, d, *J* = 8.0 Hz, 2 × ArH), 7.67 (2H, d, *J* = 7.6 Hz, 2 × ArH), and 10.54 (1H, s, CH=O). HRMS (ESI): Calcd. for C_15_H_11_ClF_3_O_2_ (M + H)^+^ 315.0394 and found 315.0398.

**2-Chloro-3-(3-chlorobenzyloxy)-benzaldehyde (38t):** 418 mg, 74%, white solid. ^1^H NMR (400 MHz, CDCl_3_): *δ* 5.16 (2H, s, CH_2_), 7.17 (1H, dd, *J* = 8.2, 1.4 Hz, ArH), 7.31 (1H, t, *J* = 8.0 Hz, ArH), 7.31–7.37 (3H, m, 3 × ArH), 7.46–7.48 (1H, m, ArH), 7.56 (1H, dd, *J* = 7.8, 1.4 Hz, ArH), and 10.54 (1H, d, *J* = 0.8 Hz, CH=O). HRMS (ESI): Calcd. for C_14_H_11_Cl_2_O_2_ (M + H)^+^ 281.0131 and found 281.0139.

**(*E*)-Amino(2-(3-(2,3-dichlorobenzyloxy)benzylidene)hydrazineyl)methaniminium acetate (10a):** A mixture of **38a** (228 mg, 0.81 mmol) and *N*-aminoguanidine bicarbonate (110 mg, 0.81 mmol) in MeOH-AcOH (3 mL/0.2 mL) was refluxed under N_2_ for 4 h, cooled to r.t., and concentrated *in vacuo.* CH_2_Cl_2_ (1 mL) was added, and the precipitate was collected, washed with CH_2_Cl_2_, and dried in vacuo to give **10a** as a white solid (170 mg, 53%, m.p. 159–160 °C). ^1^H NMR (400 MHz, DMSO-*d_6_*): *δ* 1.87 (3H, s, CH_3_CO_2_), 5.29 (2H, s, CH_2_), 7.03 (1H, d, *J* = 8.9 Hz, ArH), 7.07 (4H, br.s, 2 × NH_2_), 7.48 (t, *J* = 7.9 Hz, 1H, ArH), 7.53–7.55 (2H, m, 2 × ArH), 7.66–7.72 (2H, m, 2 × ArH), 7.58 (1H, s, ArH), and 8.08 (1H, s, CH=N). HRMS (ESI): Calcd. for C_15_H_15_Cl_2_N_4_O (M + H)^+^ 337.0623 and found 337.0693.

**(*E*)-Amino(2-(3-(2-chloro-3-(trifluoromethyl)benzyloxy)benzylidene)hydrazine-yl)-methaniminium acetate (10b):** A mixture of **38b** (320 mg, 1.02 mmol) and *N*-aminoguanidine bicarbonate (138 mg, 1.02 mmol) in MeOH-AcOH (4 mL/0.2 mL) was refluxed under N_2_ for 6 h, cooled to r.t., and concentrated *in vacuo.* EtOAc (3 mL) was added, and the precipitate was collected, washed with EtOAc, and dried in vacuo to give **10b** as a white solid (283 mg, 75%, m.p. 190–192 °C). ^1^H NMR (400 MHz, DMSO-*d_6_*): *δ* 1.82 (3H, s, CH_3_CO_2_), 5.25 (2H, s, CH_2_), 6.95 (4H, br.s, 2 × NH_2_), 7.02 (1H, dd, *J* = 7.8, 1.9 Hz, ArH), 7.26–7.33 (2H, m, 2 × ArH), 7.50 (1H, s, ArH), 7.58 (1H, t, *J* = 7.8 Hz, ArH), 7.86 (1H, d, *J* = 8.0 Hz, ArH), 7.91 (1H, d, *J* = 7.8 Hz, ArH), and 7.99 (s, 1H, CH=N). HRMS (ESI): Calcd. for C_16_H_15_ClF_3_N_4_O (M + H)^+^ 371.0886 and found 371.0895.

**(*E*)-Amino(2-(3-(2,3-dichlorobenzyloxy)-4-methoxybenzylidene)hydrazineyl)-methaniminium acetate (10g):** A white solid was obtained (278 mg, 80%). ^1^H NMR (400 MHz, DMSO-*d_6_*): *δ* 1.89 (3H, s, CH_3_CO_2_), 3.85 (3H, s, OCH_3_), 5.30 (2H, s, CH_2_), 6.98 (4H, br.s, 2 × NH_2_), 7.07 (1H, d, *J* = 8.1 Hz, ArH), 7.28 (1H, d, *J* = 8.2 Hz, ArH), 7.50 (1H, t, *J* = 7.9 Hz, ArH), 7.65–7.75 (3H, m, 3 × ArH), and 8.19 (1H, s, CH=N). HRMS (ESI): Calcd. for C_16_H_17_Cl_2_N_4_O_2_ (M + H)^+^ 367.0729 and found 367.0701.

**General procedure: Synthesis of (*E*)-amino(2-(3-(benzyloxy)benzylidene) hydrazineyl)methaniminium chloride derivatives (10c–f, 10h–t):** 3-benzyloxybenzaldehyde derivatives **36c–t** (0.2 mmol) and *N*-aminoguanidine bicarbonate (0.205 mmol) were placed in a 50 mL round-bottom flask. HCl (0.5M in MeOH, 2.0 mL) was added, and the reaction mixture was stirred at 80 °C for 0.5 h and then evaporated to dryness. Crystallisation from Et_2_O (~5–6 mL) with a very small portion of MeOH (~0.3–0.5 mL) gave the corresponding *N*-aminoguanidinium chloride or acetate salts **10c–f** or **10h–t**.

**(*E*)-Amino(2-(3-(4-chlorobenzyloxy)benzylidene)hydrazineyl)methaniminium chloride (10c):** 54 mg, 79%, white solid, m.p. 191–193 °C. ^1^H NMR (400 MHz, DMSO-*d_6_*): *δ* 5.21 (2H, s, CH_2_), 7.11–7.18 (1H, m, ArH), 7.38–7.45 (2H, m, 2 × ArH), 7.52 (2H, d, *J* = 8.4 Hz, 2 × ArH), 7.56 (2H, d, *J* = 8.4 Hz, 2 × ArH), 7.67 (1H, s, ArH), 7.86 (4H, s, br, 2 × NH_2_), 8.20 (1H, s, CH=N), and 11.97 (1H, s, N-NH). HRMS (ESI): Calcd. for C_15_H_16_ClN_4_O (M + H)^+^ 303.1007 and found 303.1013.

**(*E*)-Amino(2-(3-(4-(trifluoromethyl)benzyloxy)benzylidene) hydrazineyl) methan-iminium chloride (10d):** 58 mg, 77%, white solid, m.p. 176–179 °C. ^1^H NMR (400 MHz, DMSO-*d_6_*): *δ* 5.33 (2H, s, CH_2_), 7.14–7.20 (1H, m, ArH), 7.40–7.47 (2H, m, 2 × ArH), 7.67 (1H, s, ArH), 7.75 (2H, d, *J* = 8.0 Hz, 2 × ArH), 7.83 (2H, d, *J* = 8.4 Hz, 2 × ArH), 7.85 (4H, s, br, 2 × NH_2_), 8.20 (1H, s, CH=N), and 11.96 (1H, s, N-NH). HRMS (ESI): Calcd. for C_16_H_16_F_3_N_4_O (M + H)^+^ 337.1271 and found 337.1278.

**(*E*)-Amino(2-(3-(3-chlorobenzyloxy)benzylidene)hydrazineyl)methaniminium chloride (10e):** 69 mg, >99%, beige glass. ^1^H NMR (400 MHz, DMSO-*d_6_*): *δ* 5.22 (2H, s, CH_2_), 7.12–7.18 (1H, m, ArH), 7.42 (2H, d, *J* = 6.0 Hz, 2 × ArH), 7.45 (1H, dd, *J* = 5.0, 2.0 Hz, ArH), 7.49 (2H, d, *J* = 6.0 Hz, 2 × ArH), 7.59 (1H, s, ArH), 7.66 (1H, d, *J* = 2.8 Hz, ArH), 7.84 (4H, s, br, 2 × NH_2_), 8.21 (1H, s, CH=N), and 11.97 (1H, s, N-NH). HRMS (ESI): Calcd. for C_15_H_16_ClN_4_O (M + H)^+^ 303.1007 and found 303.1015.

**(*E*)-Amino(2-(3-(4-(trifluoromethyl)benzyloxy)benzylidene)hydrazineyl)methan-iminium chloride (10f):** 68 mg, 92%, pale yellow glass. ^1^H NMR (400 MHz, DMSO-*d_6_*): *δ* 5.31 (2H, s, CH_2_), 7.15–7.21 (1H, m, ArH), 7.41–7.45 (2H, m, 2 × ArH), 7.69 (1H, d, *J* = 2.8 Hz, ArH), 7.71 (1H, d, *J* = 8.4 Hz, ArH), 7.76 (1H, d, *J* = 8.0 Hz, ArH), 7.84 (1H, d, *J* = 7.6 Hz, ArH), 7.86 (4H, s, br, 2 × NH_2_), 7.89 (1H, s, ArH), 8.22 (1H, s, CH=N), and 11.97 (1H, s, N-NH). HRMS (ESI): Calcd. for C_16_H_16_F_3_N_4_O (M + H)^+^ 337.1271 and found 337.1282.

**(*E*)-Amino(2-(2-chloro-3-(2-chloro-3-methoxybenzyloxy)benzylidene) hydrazine-yl)methaniminium chloride (10h):** 68 mg, 84%, white solid, m.p. 258–260 °C. ^1^H NMR (400 MHz, DMSO-*d_6_*): *δ* 5.33 (2H, s, CH_2_), 7.23 (1H, dd, *J* = 8.0, 1.2 Hz, ArH), 7.28 (1H, dd, *J* = 7.6 Hz, 1.2, ArH), 7.37 (1H, dd, *J* = 8.0, 1.6 Hz, ArH), 7.42 (1H, d, *J* = 8.0 Hz, ArH), 7.43 (1H, t, *J* = 7.8 Hz, ArH), 7.93 (4H, s, br, 2 × NH_2_), 7.96 (1H, dd, *J* = 7.8, 1.4 Hz, ArH), 8.65 (1H, s, CH=N), and 12.26 (1H, s, N-NH). HRMS (ESI): Calcd. for C_16_H_17_Cl_2_N_4_O_2_ (M + H)^+^ 367.0723 and found 367.0734.

**(*E*)-Amino(2-(3-(benzyloxy)-2-chlorobenzylidene)hydrazineyl)methaniminium chloride (10i):** 56 mg, 82%, white solid, m.p. 203–205 °C. ^1^H NMR (400 MHz, DMSO-*d_6_*): *δ* 5.30 (2H, s, CH_2_), 7.37 (1H, dd, *J* = 8.4, 2.0 Hz, ArH), 7.39–7.44 (2H, m, 2 × ArH), 7.47 (2H, t, *J* = 7.4 Hz, 2 × ArH), 7.53 (1H, s, ArH), 7.55 (1H, d, *J* = 1.6 Hz, ArH), 7.92 (4H, s, br, 2 × NH_2_), 7.94 (1H, dd, *J* = 7.6, 1.6 Hz, ArH), 8.65 (1H, s, CH=N), and 12.27 (1H, s, N-NH). HRMS (ESI): Calcd. for C_15_H_16_ClN_4_O (M + H)^+^ 303.1007 and found 303.1012.

**(*E*)-Amino(2-(2-chloro-3-(4-chlorobenzyloxy)benzylidene)hydrazineyl)methan-iminium chloride (10j):** 63 mg, 84%, white solid, m.p. 237–239 °C. ^1^H NMR (400 MHz, DMSO-*d_6_*): *δ* 5.30 (2H, s, CH_2_), 7.35 (1H, dd, *J* = 8.0, 1.2 Hz, ArH), 7.42 (1H, t, *J* = 8.0 Hz, ArH), 7.51–7.59 (4H, m, 4 × ArH), 7.93 (4H, s, br, 2 × NH_2_), 7.95 (1H, dd, *J* = 7.6, 1.6 Hz, ArH), 8.65 (1H, s, CH=N), and 12.32 (1H, s, N-NH). HRMS (ESI): Calcd. for C_15_H_15_Cl_2_N_4_O (M + H)^+^ 337.0617 and found 337.0625.

**(*E*)-Amino(2-(2-chloro-3-(2,3-dichlorobenzyloxy)benzylidene)hydrazineyl) meth-animinium chloride (10k):** 71 mg, 87%, white solid, m.p. 250–253 °C. ^1^H NMR (400 MHz, DMSO-*d_6_*): *δ* 5.38 (2H, s, CH_2_), 7.40 (1H, dd, *J* = 8.4, 1.6 Hz, ArH), 7.46 (1H, t, *J* = 7.6 Hz, ArH), 7.51 (1H, t, *J* = 8.0 Hz, ArH), 7.70 (1H, dd, *J* = 7.8, 1.4 Hz, ArH), 7.73 (1H, dd, *J* = 8.0, 1.2 Hz, ArH), 7.95 (4H, s, br, 2 × NH_2_), 7.98 (1H, dd, *J* = 7.6, 1.6 Hz, ArH), 8.65 (1H, s, CH=N), and 12.30 (1H, s, N-NH). HRMS (ESI): Calcd. for C_15_H_14_Cl_3_N_4_O (M + H)^+^ 371.0228 and found 371.0235.

**(*E*)-Amino(2-(2-chloro-3-(2-chloro-3-(trifluoromethyl)benzyloxy)benzylidene) hydrazineyl)methaniminium chloride (10m):** 73 mg, 82%, white solid, m.p. 274–277 °C. ^1^H NMR (400 MHz, DMSO-*d_6_*): *δ* 5.44 (2H, s, CH_2_), 7.42–7.48 (2H, m, 2 × ArH), 7.71 (1H, t, *J* = 7.8 Hz, ArH), 7.95 (1H, d, *J* = 8.0 Hz, ArH), 7.97 (4H, s, br, 2 × NH_2_), 7.99 (1H, dd, *J* = 7.0, 1.4 Hz, ArH), 8.04 (1H, d, *J* = 7.6 Hz, ArH), 8.66 (1H, s, CH=N), and 12.31 (1H, s, N-NH). ^13^C NMR (100 MHz, DMSO-*d_6_*): *δ* 67.6 (CH_2_), 115.5 (CH), 119.8 (CH), 122.3, 127.6 (CH), 127.8 (CH), 127.9 (CH), 132.0, 133.5 (CH), 136.7, 142.9 (CH), 153.5, and 155.2. HRMS (ESI): Calcd. for C_16_H_14_Cl_2_F_3_N_4_O (M + H)^+^ 405.0491 and found 405.0498.

**(*E*)-Amino(2-(2-chloro-3-(2,4-dichlorobenzyloxy)benzylidene)hydrazineyl) meth-animinium chloride (10n):** 73 mg, 89%, white solid, m.p. 268–270 °C. ^1^H NMR (400 MHz, DMSO-*d_6_*): *δ* 5.32 (2H, s, CH_2_), 7.40 (1H, dd, *J* = 8.4, 1.6 Hz, ArH), 7.44 (1H, t, *J* = 8.0 Hz, ArH), 7.58 (1H, dd, *J* = 8.4, 2.0 Hz, ArH), 7.73 (1H, d, *J* = 8.4 Hz, ArH), 7.77 (1H, d, *J* = 2.4 Hz, ArH), 7.95 (4H, s, br, 2 × NH_2_), 7.98 (1H, dd, *J* = 7.6, 1.6 Hz, ArH), 8.65 (1H, s, CH=N), and 12.34 (1H, s, N-NH). HRMS (ESI): Calcd. for C_15_H_14_Cl_3_N_4_O (M + H)^+^ 371.0228 and found 371.0239.

**(*E*)-Amino(2-(2-chloro-3-(2,5-dichlorobenzyloxy)benzylidene)hydrazineyl) meth-animinium chloride (10o):** 72 mg, 88%, white solid, m.p. 242–244 °C. ^1^H NMR (400 MHz, DMSO-*d_6_*): *δ* 5.33 (2H, s, CH_2_), 7.42 (1H, dd, *J* = 7.4, 1.6 Hz, ArH), 7.46 (1H, t, *J* = 7.6 Hz, ArH), 7.56 (1H, dd, *J* = 8.6, 2.2 Hz, ArH), 7.64 (1H, d, *J* = 8.8 Hz, ArH), 7.79 (1H, d, *J* = 2.4 Hz, ArH), 7.93 (4H, s, br, 2 × NH_2_), 7.99 (1H, dd, *J* = 7.2, 1.6 Hz, ArH), 8.65 (1H, s, CH=N), and 12.25 (1H, s, N-NH). HRMS (ESI): Calcd. for C_15_H_14_Cl_3_N_4_O (M + H)^+^ 371.0228 and found 371.0237.

**(*E*)-Amino(2-(2-chloro-3-(3,4-dichlorobenzyloxy)benzylidene)hydrazineyl) meth-animinium chloride (10p):** 69 mg, 84%, white solid, m.p. 230–233 °C. ^1^H NMR (400 MHz, DMSO-*d_6_*): *δ* 5.32 (2H, s, CH_2_), 7.35 (1H, dd, *J* = 8.4, 1.2 Hz, ArH), 7.43 (1H, t, *J* = 7.8 Hz, ArH), 7.53 (1H, dd, *J* = 8.0, 2.0 Hz, ArH), 7.75 (1H, d, *J* = 8.4 Hz, ArH), 7.81 (1H, d, *J* = 2.0 Hz, ArH), 7.92 (4H, s, br, 2 × NH_2_), 7.96 (1H, dd, *J* = 8.0, 1.2 Hz, ArH), 8.65 (1H, s, CH=N), and 12.25 (1H, s, N-NH). HRMS (ESI): Calcd. for C_15_H_14_Cl_3_N_4_O (M + H)^+^ 371.0228 and found 371.0239.

**(*E*)-Amino(2-(2-chloro-3-(2,3,5-trichlorobenzyloxy)benzylidene)hydrazineyl) methaniminium chloride (10q):** 52 mg, 59%, white solid, m.p. 273–276 °C. ^1^H NMR (400 MHz, DMSO-*d_6_*): *δ* 5.36 (2H, s, CH_2_), 7.42 (1H, dd, *J* = 7.4, 2.0 Hz, ArH), 7.46 (1H, t, *J* = 7.6 Hz, ArH), 7.77 (1H, d, *J* = 2.4 Hz, ArH), 7.96 (4H, s, br, 2 × NH_2_), 7.96 (1H, d, *J* = 2.8 Hz, ArH), 7.99 (1H, dd, *J* = 7.4, 1.8 Hz, ArH), 8.65 (1H, s, CH=N), and 12.32 (1H, s, N-NH). HRMS (ESI): Calcd. for C_15_H_13_Cl_4_N_4_O (M + H)^+^ 404.9838 and found 404.9851.

**(*E*)-Amino(2-(2-chloro-3-(3-(trifluoromethyl)benzyloxy)benzylidene)hydrazineyl) methaniminium chloride (10r):** 69 mg, 84%, white powder, m.p. 198–201 °C. ^1^H NMR (400 MHz, DMSO-*d_6_*): *δ* 5.41 (2H, s, CH_2_), 7.39 (1H, d, *J* = 8.4 Hz, ArH), 7.44 (1H, t, *J* = 7.8 Hz, ArH), 7.73 (1H, t, *J* = 7.6 Hz, ArH), 7.78 (1H, d, *J* = 8.0 Hz, ArH), 7.85 (1H, d, *J* = 7.2 Hz, ArH), 7.91 (1H, s, ArH), 7.94 (4H, s, br, 2 × NH_2_), 7.97 (1H, d, *J* = 7.6 Hz, ArH), 8.65 (1H, s, CH=N), and 12.33 (1H, s, N-NH). HRMS (ESI): Calcd. for C_16_H_15_ClF_3_N_4_O (M + H)^+^ 371.0881 and found 371.0891.

**(*E*)-Amino(2-(2-chloro-3-(4-(trifluoromethyl)benzyloxy)benzylidene)hydrazineyl) methaniminium chloride (10s):** 65 mg, 80%, white solid, m.p. 245–247 °C. ^1^H NMR (400 MHz, DMSO-*d_6_*): *δ* 5.43 (2H, s, CH_2_), 7.36 (1H, d, *J* = 8.0 Hz, ArH), 7.43 (1H, t, *J* = 8.0 Hz, ArH), 7.76 (2H, d, *J* = 8.0 Hz, 2 × ArH), 7.85 (2H, d, *J* = 7.6 Hz, 2 × ArH), 7.95 (4H, s, br, 2 × NH_2_), 7.96 (1H, d, *J* = 7.6 Hz, ArH), 8.66 (1H, s, CH=N), and 12.31 (1H, s, N-NH). HRMS (ESI): Calcd. for C_16_H_15_ClF_3_N_4_O (M + H)^+^ 371.0881 and found 371.0887.

**(*E*)-Amino(2-(2-chloro-3-(3-chlorobenzyloxy)benzylidene)hydrazineyl) methaniminium chloride (10t):** 75 mg, >99%, pale yellow glass. ^1^H NMR (400 MHz, DMSO-*d_6_*): *δ* 5.31 (2H, s, CH_2_), 7.35 (1H, dd, *J* = 8.0, 1.2 Hz, ArH), 7.41 (1H, t, *J* = 8.0 Hz, ArH), 7.43–7.48 (1H, m, ArH), 7.48–7.51 (2H, m, 2 × ArH), 7.59 (1H, s, ArH), 7.92 (4H, s, br, 2 × NH_2_), 7.94 (1H, dd, *J* = 8.0, 1.2 Hz, ArH), 8.65 (1H, s, CH=N), and 12.23 (1H, s, N-NH). HRMS (ESI): Calcd. for C_15_H_15_Cl_2_N_4_O (M + H)^+^ 337.0617 and found 337.0629.

**General Procedure: Synthesis of 1-benzyl-1*H*-imidazole-2-carbaldehyde derivatives (40a–d**) and 1-benzyl-1*H*-pyrrole-2-carbaldehyde derivatives (**40e–h**): Imidazole 2-carboxaldehyde 39a (1 mmol) or pyrrole 2-carboxaldehyde **39b** (1 mmol) and K_2_CO_3_ (4 mmol) were placed in a 25 mL round-bottom flask. DMF (1 mL) was added. Then, the corresponding benzyl halide (1.2 mmol) was added and the reaction mixture was stirred at r.t. for 18 h. Water (80 mL) and brine (20 mL) were added, and the mixture was extracted with Et_2_O (100 mL). The organic layer was dried (NaCl), filtered, and concentrated in vacuo. All crude compounds **40a–d** were purified by flash column chromatography.

**1-(3-Chlorobenzyl)-1*H*-imidazole-2-carbaldehyde (40a):** Purification by flash column chromatography (CH_2_Cl_2_ to CH_2_Cl_2_/EtOAc 9:1) gave **40a** (177 mg, 80%, colourless oil). ^1^H NMR (400 MHz, CDCl_3_): *δ* 5.58 (2H, s, CH_2_), 7.05–7.08 (1H, m, ArH), 7.15 (2H, s, 2 × ArH), 7.25–7.28 (2H, m, 2 × ArH), 7.32 (1H, s, ArH), and 9.84 (1H, d, *J* = 0.5 Hz, CH=O). HRMS (ESI): Calcd. for C_11_H_10_ClN_2_O (M + H)^+^ 221.0476 and found 221.0469.

**1-(4-Chlorobenzyl)-1*H*-imidazole-2-carbaldehyde (40b):** Purification by flash column chromatography (CH_2_Cl_2_ to CH_2_Cl_2_/EtOAc 9:1) gave **40b** (184 mg, 83%, beige solid). ^1^H NMR (400 MHz, CDCl_3_): *δ* 5.57 (2H, s, CH_2_), 7.11–7.13 (1H, m, ArH), 7.13–7.15 (2H, m, 2 × ArH), 7.30 (2H, dt, *J* = 8.4, 2.2 Hz, 2 × ArH), 7.32 (1H, s, ArH), and 9.86 (1H, s, CH=O). HRMS (ESI): Calcd. for C_11_H_10_ClN_2_O (M + H)^+^ 221.0476 and found 221.0471.

**1-(3-(Trifluoromethyl)benzyl)-1*H*-imidazole-2-carbaldehyde (40c):** Purification by flash column chromatography (CH_2_Cl_2_ to CH_2_Cl_2_/EtOAc 9:1) gave **40c** (197 mg, 77%, colourless oil). ^1^H NMR (400 MHz, DMSO-*d_6_*): *δ* 5.75 (2H, s, CH_2_), 7.42 (1H, d, *J* = 0.8 Hz, ArH), 7.52 (1H, d, *J* = 8.0 Hz, ArH), 7.62–7.67 (2H, m, 2 × ArH), 7.70–7.74 (1H, m, ArH), 7.90 (1H, s, ArH), and 9.75 (1H, d, *J* = 0.8 Hz, CH=O). HRMS (ESI): Calcd. for C_12_H_10_F_3_N_2_O (M + H)^+^ 255.0740 and found 255.0747.

**1-(4-(Trifluoromethyl)benzyl)-1*H*-imidazole-2-carbaldehyde (40d):** Purification by flash column chromatography (CH_2_Cl_2_ to CH_2_Cl_2_/EtOAc 9:1) gave **40d** (100 mg, 39%, beige solid). ^1^H NMR (400 MHz, CDCl_3_): *δ* 5.68 (2H, s, CH_2_), 7.19 (1H, s, ArH), 7.29 (2H, d, *J* = 8.0 Hz, 2 × ArH), 7.37 (1H, s, ArH), 7.60 (2H, d, *J* = 8.0 Hz, 2 × ArH), and 9.88 (1H, s, CH=O). HRMS (ESI): Calcd. for C_12_H_10_F_3_N_2_O (M + H)^+^ 255.0740 and found 255.0749.

**1-(3-Chlorobenzyl)-1*H*-pyrrole-2-carbaldehyde (40e):** Purification by flash column chromatography (petrol ether to petrol ether/EtOAc 9:1) gave **40e** (165 mg, 75%, colourless oil). ^1^H NMR (400 MHz, CDCl_3_): *δ* 5.53 (2H, s, CH_2_), 6.29 (1H, dd, *J* = 3.8, 2.6 Hz, ArH), 6.96–6.99 (2H, m, 2 × ArH), 7.07–7.09 (1H, m, ArH), 7.21–7.23 (2H, m, 2 × ArH), 7.25–7.27 (1H, m, ArH), and 9.54 (1H, d, *J* = 0.8 Hz, CH=O). HRMS (ESI): Calcd. for C_12_H_11_ClNO (M + H)^+^ 220.0524 and found 220.0519.

**1-(4-Chlorobenzyl)-1*H*-pyrrole-2-carbaldehyde (40f):** Purification by flash column chromatography (petrol ether to petrol ether/EtOAc 9:1) gave **40f** (185 mg, 84%, beige solid). ^1^H NMR (400 MHz, CDCl_3_): *δ* 5.51 (2H, s, CH_2_), 6.28 (1H, t, *J* = 3.2 Hz, ArH), 6.95–6.98 (2H, m, 2 × ArH), 7.07 (2H, dt, *J* = 8.4, 2.2 Hz, 2 × ArH), 7.26 (2H, dt, *J* = 8.4, 2.2 Hz, 2 × ArH), and 9.54 (1H, s, CH=O). HRMS (ESI): Calcd. for C_12_H_11_ClNO (M + H)^+^ 220.0524 and found 220.0521.

**1-(3-(Trifluoromethyl)benzyl)-1*H*-pyrrole-2-carbaldehyde (40g):** Purification by flash column chromatography (petrol ether to petrol ether/EtOAc 9:1) gave **40g** (216 mg, 85%, beige-brown oil). ^1^H NMR (400 MHz, CDCl_3_): *δ* 5.61 (2H, s, CH_2_), 6.31 (1H, t, *J* = 3.2 Hz, ArH), 6.98–7.01 (2H, m, 2 × ArH), 7.30 (1H, d, *J* = 7.6 Hz, ArH), 7.37 (1H, s, ArH), 7.42 (1H, t, *J* = 7.8 Hz, ArH), 7.52 (1H, d, *J* = 8.0 Hz, ArH), and 9.55 (1H, s, CH=O). HRMS (ESI): Calcd. for C_13_H_11_F_3_NO (M + H)^+^ 254.0787 and found 254.0793.

**1-(4-(Trifluoromethyl)benzyl)-1*H*-pyrrole-2-carbaldehyde (40h):** Purification by flash column chromatography (petrol ether to petrol ether/EtOAc 9:1) gave **40h** (82 mg, 32%, beige solid). ^1^H NMR (400 MHz, CDCl_3_): *δ* 5.61 (2H, s, CH_2_), 6.31 (1H, t, *J* = 3.0 Hz, ArH), 6.98–7.01 (2H, m, 2 × ArH), 7.21 (2H, d, *J* = 8.0 Hz, 2 × ArH), 7.55 (2H, d, *J* = 8.0 Hz, 2 × ArH), and 9.54 (1H, s, CH=O). HRMS (ESI): Calcd. for C_13_H_11_F_3_NO (M + H)^+^ 254.0787 and found 254.0795.

**General Procedure: Synthesis of (*E*)-amino(2-((1-benzyl-1*H*-imidazol-2-yl)methylene)hydrazineyl)methaniminium chloride derivatives (41a–d) and (*E*)-Amino(2-((1-benzyl-1*H*-pyrrol-2-yl)methylene)hydrazineyl)methaniminium chloride derivatives** (**41e–h**): Aldehyde derivatives (**40a–h**) (0.2 mmol) and *N*-aminoguanidine bicarbonate (0.24 mmol) were placed in a 50 mL round-bottom flask. HCl (0.5M in MeOH, 2.0 mL) was added, and the reaction mixture was stirred at 80 °C for 2 h and then evaporated to dryness.

**(*E*)-Amino(2-((1-(3-chlorobenzyl)-1*H*-imidazol-2-yl)methylene)hydrazineyl) methaniminium chloride (41a):** 87 mg, >99%, pale yellow glass. ^1^H NMR (400 MHz, DMSO-*d_6_*): *δ* 5.71 (2H, s, CH_2_), 7.35 (1H, dt, *J* = 4.4, 1.6 Hz, ArH), 7.46–7.50 (2H, m, 2 × ArH), 7.52 (1H, s, ArH), 7.88 (1H, d, *J* = 2.2 Hz, ArH), 7.94 (1H, d, *J* = 2.2 Hz, ArH), 8.35 (4H, s, br, 2 × NH_2_), 8.56 (1H, s, CH=N), and 12.85 (1H, s, br, N-NH). HRMS (ESI): Calcd. for C_12_H_14_ClN_6_ (M + H)^+^ 277.0963 and found 277.0956.

**(*E*)-Amino(2-((1-(4-chlorobenzyl)-1*H*-imidazol-2-yl)methylene)hydrazineyl) methaniminium chloride (41b):** 88 mg, >99%, pale yellow glass. ^1^H NMR (400 MHz, DMSO-*d_6_*): *δ* 5.70 (2H, s, CH_2_), 7.42 (2H, dt, *J* = 8.4, 2.2 Hz, 2 × ArH), 7.52 (2H, dt, *J* = 8.4, 2.2 Hz, 2 × ArH), 7.86 (1H, d, *J* = 1.8 Hz, ArH), 7.91 (1H, d, *J* = 1.8 Hz, ArH), 8.35 (4H, s, br, 2 × NH_2_), 8.53 (1H, s, CH=N), and 12.82 (1H, s, br, N-NH). HRMS (ESI): Calcd. for C_12_H_14_ClN_6_ (M + H)^+^ 277.0963 and found 277.0955.

**(*E*)-Amino(2-((1-(3-(trifluoromethyl)benzyl)-1*H*-imidazol-2-yl)methylene) hydrazineyl)methaniminium chloride (41c):** 94 mg, >99%, pale yellow glass. ^1^H NMR (400 MHz, DMSO-*d_6_*): *δ* 5.80 (2H, s, CH_2_), 7.65 (1H, d, *J* = 7.8 Hz, ArH), 7.70 (1H, t, *J* = 7.8 Hz, ArH), 7.80 (1H, d, *J* = 7.6 Hz, ArH), 7.85 (1H, s, ArH), 7.90 (1H, d, *J* = 1.8 Hz, ArH), 7.94 (1H, d, *J* = 1.8 Hz, ArH), 8.35 (4H, s, br, 2 × NH_2_), 8.59 (1H, s, CH=N), and 12.85 (1H, s, br, N-NH). HRMS (ESI): Calcd. for C_13_H_14_F_3_N_6_ (M + H)^+^ 311.1227 and found 311.1234.

**(*E*)-Amino(2-((1-(4-(trifluoromethyl)benzyl)-1*H*-imidazol-2-yl)methylene) hydrazineyl)methaniminium chloride (41d):** 95 mg, >99%, pale yellow glass. ^1^H NMR (400 MHz, DMSO-*d_6_*): *δ* 5.83 (2H, s, CH_2_), 7.58 (2H, d, *J* = 8.0 Hz, 2 × ArH), 7.82 (2H, d, *J* = 8.0 Hz, 2 × ArH), 7.88 (1H, d, *J* = 1.6 Hz, ArH), 7.94 (1H, d, *J* = 1.6 Hz, ArH), 8.29 (4H, s, br, 2 × NH_2_), 8.51 (1H, s, CH=N), and 12.73 (1H, s, br, N-NH). HRMS (ESI): Calcd. for C_13_H_14_F_3_N_6_ (M + H)^+^ 311.1227 and found 311.1235.

**(*E*)-Amino(2-((1-(3-chlorobenzyl)-1*H*-pyrrol-2-yl)methylene)hydrazineyl) methan-iminium chloride (41e):** 64 mg, >99%, purple-black glass. ^1^H NMR (400 MHz, DMSO-*d_6_*): *δ* 5.61 (2H, s, CH_2_), 6.29 (1H, dd, *J* = 3.8, 2.6 Hz, ArH), 6.77 (1H, dd, *J* = 3.8, 1.8 Hz, ArH), 7.01 (1H, dt, *J* = 7.2, 1.6 Hz, ArH), 7.07 (1H, t, *J* = 1.6 Hz, ArH), 7.26 (1H, dd, *J* = 2.4, 2.0 Hz, ArH), 7.35 (1H, dt, *J* = 8.0, 1.8 Hz, ArH), 7.39 (1H, t, *J* = 7.6 Hz, ArH), 7.59 (4H, s, br, 2 × NH_2_), 8.09 (1H, s, CH=N), and 11.55 (1H, s, N-NH). HRMS (ESI): Calcd. for C_13_H_15_ClN_5_ (M + H)^+^ 276.1010 and found 276.1006.

**(*E*)-Amino(2-((1-(4-chlorobenzyl)-1*H*-pyrrol-2-yl)methylene)hydrazineyl) methan-iminium chloride (41f):** 64 mg, >99%, purple-black glass. ^1^H NMR (400 MHz, DMSO-*d_6_*): *δ* 5.58 (2H, s, CH_2_), 6.27 (1H, dd, *J* = 3.8, 1.4 Hz, ArH), 6.76 (1H, dd, *J* = 3.8, 1.8 Hz, ArH), 7.07 (2H, d, *J* = 8.0 Hz, 2 × ArH), 7.25 (1H, dd, *J* = 6.4, 1.6 Hz, ArH), 7.42 (2H, d, *J* = 8.0 Hz, 2 × ArH), 7.49 (4H, s, br, 2 × NH_2_), 8.07 (1H, s, CH=N), and 11.54 (1H, s, N-NH). HRMS (ESI): Calcd. for C_13_H_15_ClN_5_ (M + H)^+^ 276.1010 and found 276.1004.

**(*E*)-Amino(2-((1-(3-(trifluoromethyl)benzyl)-1*H*-pyrrol-2-yl)methylene) hydrazin-eyl)methaniminium chloride (41g):** 81 mg, >99%, purple-black glass. ^1^H NMR (400 MHz, DMSO-*d_6_*): *δ* 5.72 (2H, s, CH_2_), 6.30 (1H, dd, *J* = 3.8, 2.6 Hz, ArH), 6.77 (1H, dd, *J* = 3.8, 1.8 Hz, ArH), 7.28–7.32 (2H, m, 2 × ArH), 7.44 (1H, s, ArH), 7.50 (4H, s, br, 2 × NH_2_), 7.60 (1H, t, *J* = 7.8 Hz, ArH), 7.65 (1H, d, *J* = 7.6 Hz, ArH), 8.08 (1H, s, CH=N), and 11.57 (1H, s, N-NH). HRMS (ESI): Calcd. for C_14_H_15_F_3_N_5_ (M + H)^+^ 310.1274 and found 310.1278.

**(*E*)-Amino(2-((1-(4-(trifluoromethyl)benzyl)-1*H*-pyrrol-2-yl)methylene) hydrazin-eyl)methaniminium chloride (41h):** 80 mg, >99%, purple-black glass. ^1^H NMR (400 MHz, DMSO-*d_6_*): *δ* 5.71 (2H, s, CH_2_), 6.31 (1H, t, *J* = 2.8 Hz, ArH), 6.78–6.79 (1H, m, ArH), 7.23 (2H, d, *J* = 8.0 Hz, 2 × ArH), 7.27 (1H, t, *J* = 2.6 Hz, ArH), 7.46 (4H, s, br, 2 × NH_2_), 7.73 (2H, d, *J* = 8.0 Hz, 2 × ArH), 8.07 (1H, s, CH=N), and 11.57 (1H, s, N-NH). HRMS (ESI): Calcd. for C_14_H_15_F_3_N_5_ (M + H)^+^ 310.1274 and found 310.1281.

***tert*-Butyl *N*-[{[(*tert*-butoxy)carbonyl]imino}[(4-hydroxyphenyl)amino] methyl]-carbamate (43):** In a solution of 4-aminophenol **42** (273 mg, 2.5 mmol) in DMF (6 mL), *S*-methyl-*N*,*N*’-bis(*tert*-butoxycarbonyl)isothiourea (690 mg, 2.37 mmol) was added, followed by Et_3_N (0.7 mL) and HgCl_2_ (640 mg, 2.37 mmol). The mixture was stirred at r.t. overnight, and then diluted with EtOAc (30 mL) and filtered through Celite. The organic layer was washed with brine, dried (MgSO_4_), and concentrated *in vacuo.* Purification by flash chromatography (petrol ether to petrol ether/EtOAc 3:2) afforded **43** as a white solid (535 mg, 64% yield), m.p. 182–184 °C. ^1^H NMR (400 MHz, CDCl_3_): *δ* 1.44 (9H, s, *t*-Bu), 1.47 (9H, s, *t*-Bu), 6.56 (2H, br.s, 2 × ArH), 7.02 (3H, br.s, 2 × ArH and OH), 9.93 (1H, s, NH), and 11.6 (1H, s, NH). HRMS (ESI): Calcd. for C_17_H_25_N_3_NaO_5_ (M + Na)^+^ 374.1692 and found 374.1697.

**General Procedure: Benzylation of 4-(*N,N*′-di-Boc-guanydino)phenol (43):** In a solution of 4-(*N,N*′-di-Boc-guanydino)phenol **43** (110 mg, 0.53 mmol) in acetone (5 mL), the substituted benzyl bromide (0.37 mmol) was added, followed by K_2_CO_3_ (72 mg, 0.52 mmol). The mixture was stirred at r.t. overnight and partitioned between EtOAc (30 mL) and water (30 mL). The organic layer was washed with brine, dried (MgSO_4_), and concentrated in vacuo to give an off-white solid. Purification by flash column chromatography (petrol ether to petrol ether/EtOAc 3:1) afforded **44a–c**.

***tert*-Butyl *N*-[{[4-(benzyloxy)phenyl]amino}({[(*tert*-butoxy)carbonyl]imino}) meth-yl]-carbamate (44a):** A white solid was obtained (79% yield), m.p. 141–143 °C. ^1^H NMR (400 MHz, CDCl_3_): *δ* 1.48 (9H, s, *t*-Bu), 1.52 (9H, s, *t*-Bu), 5.05 (2H, s, OCH_2_), 6.92 (2H, d, *J* = 8.2 Hz, 2 × ArH), 7.30–7.42 (5H, m, 5 × ArH), 7.47 (2H, d, *J* = 8.3 Hz, 2 × ArH), 10.2 (1H, br.s, NH), and 11.6 (1H, s, NH). HRMS (ESI): Calcd. for C_24_H_31_N_3_NaO_5_ (M + Na)^+^ 464.2161 and found 464.2179.

***tert*-Butyl *N*-[{[(*tert*-butoxy)carbonyl]imino}({4-[(4-chlorophenyl)methoxy] phenyl}amino)methyl]carbamate (44b):** A white solid was obtained (89% yield), m.p. 95–97 °C. ^1^H NMR (400 MHz, CDCl_3_): *δ* 1.48 (9H, s, *t*-Bu), 1.52 (9H, s, *t*-Bu), 5.00 (2H, s, OCH_2_), 6.91 (2H, dd, *J* = 7.8, 2.4 Hz, 2 × ArH), 7.30 (4H, s, 4 × ArH), 7.51 (2H, dd, *J* = 7.8, 2.4 Hz, 2 × ArH), 10.2 (1H, br.s, NH), and 11.6 (1H, s, NH).

***tert*-Butyl *N*-[{[3-(benzyloxy)phenyl]amino}({[(*tert*-butoxy)carbonyl]imino}) methyl]carbamate (44c):** A white solid was obtained (62% yield), m.p. 79–83 °C. ^1^H NMR (400 MHz, CDCl_3_): *δ* 1.47 (9H, s, *t*-Bu), 1.51 (9H, s, *t*-Bu), 5.05 (2H, s, OCH_2_), 6.92 (2H, d, *J* = 8.4 Hz, 2 × ArH), 7.25–7.36 (4H, m, 4 × ArH), 7.40 (2H, d, *J* = 8.3 Hz, 2 × ArH), 10.8 (1H, br.s, NH), and 11.8 (1H, s, NH).

**General Procedure: Synthesis of the phenyl guanidine derivatives (45a–c):** In a solution of the *para*-substituted *N,N*′-di-Boc-(4-guanydino)benzene (100 mg) (**44a–c)** in CH_2_Cl_2_ (2 mL), TFA (1 mL) was added. The mixture was shaken at r.t. overnight and then evaporated to dryness. Et_2_O (1 mL) was added, and the precipitate was collected, washed with Et_2_O, and dried in vacuo to give **45a–c** as a white or off-white solid.

**1-[4-(Benzyloxy)phenyl]guanidinium 2,2,2-trifluoroacetate (45a):** A white solid was obtained (95% yield). ^1^H NMR (400 MHz, CD_3_OD): *δ* 5.12 (2H, s, OCH_2_), 7.12 (2H, d, *J* = 8.1 Hz, 2 × ArH), 7.22 (2H, d, *J* = 8.1 Hz, 2 × ArH), 7.32–7.40 (3H, m, 3 × ArH), and 7.46 (2H, d, *J* = 8.3 Hz, 2 × ArH). HRMS (ESI): Calcd. for C_14_H_16_N_3_O (M + H)^+^ 242.1293 and found 242.1283.

**1-[4-(4-Chlorobenzyloxy)phenyl]guanidinium 2,2,2-trifluoroacetate (45b):** A white solid was obtained (88% yield). ^1^H NMR (400 MHz, CD_3_OD): *δ* 5.12 (2H, s, OCH_2_), 7.21–7.30 (4H, m, 4 × ArH), 7.37 (2H, d, *J* = 8.2 Hz, 2 × ArH), and 7.55 (2H, d, *J* = 8.2 Hz, 2 × ArH). HRMS (ESI): Calcd. for C_14_H_15_ClN_3_O (M + H)^+^ 276.0904 and found 276.0932.

**1-[4-(3-Chlorobenzyloxy)phenyl}guanidinium 2,2,2-trifluoroacetate (45c):** A white solid was obtained (92% yield). ^1^H NMR (400 MHz, CD_3_OD): *δ* 5.21 (s, 2H, OCH_2_), 7.32 (2H, d, *J* = 8.0 Hz, 2 × ArH), 7.38 (1H, s, ArH), 7.47–7.53 (3H, m, 3 × ArH), and 7.59 (2H, d, *J* = 8.3 Hz, 2 × ArH). HRMS (ESI) calcd. for C_14_H_15_ClN_3_O (M + H)^+^ 276.0904 and found 276.0911.

**General Procedure: Synthesis of Boc-protected amide/sulphonamide derivative (47a-b):** 4-Aminobenzylamine **21** (0.5 mmol) was dissolved in DMF (1.0 mL) and Et_3_N (2.0 mmol). Benzoyl chloride or benzenesulphonyl chloride (0.5 mmol) was added successively, and the reaction mixture was stirred for 2 h at 0 °C. HgCl_2_ (0.5 mmol) and *S*-methyl-*N,N*′-bis(*tert*-butoxycarbonyl) isothiourea (0.5 mmol) were added, and the reaction mixture was stirred at r.t. for 18 h. Et_2_O (100 mL) was added, and the mixture was filtered through a paper filter. The organic layer was washed with water (80 mL) and brine (20 mL), dried (NaCl), filtered, and concentrated to dryness. Crystallisation from Et_2_O gave **47a–b**.

***tert*-Butyl *N*-[(1*Z*)-{[(*tert*-butoxy)carbonyl]imino}({4-[(phenylformamido)methyl] phenyl}amino)methyl]carbamate (47a):** 113 mg, 48%, white solid, m.p. 152–156 °C, mixture of conformers as revealed by ^1^H NMR. Major conformer: ^1^H NMR (400 MHz, CDCl_3_): *δ* 1.45 (9H, s, *t*-Bu), 1.53 (9H, s, *t*-Bu), 4.55 (2H, d, *J* = 5.2 Hz, CH_2_), 6.64 (1H, br.s, NH), 7.25 (2H, d, *J* = 8.0 Hz, 2 × ArH), 7.39–7.52 (5H, m, 5 × ArH), 7.81 (2H, d, *J* = 8.0 Hz, 2 × ArH), 10.40 (1H, br.s, NH), and 11.66 (1H, br.s, NH). HRMS (ESI): Calcd. for C_25_H_33_N_4_O_5_ (M + H)^+^ 469.2445 and found 469.2451.

***tert*-Butyl *N*-[(1*Z*)-{[4-(benzenesulfonamidomethyl)phenyl]amino}({[(*tert*-butoxy)-carbonyl]imino})methyl]carbamate (47b):** 128 mg, 50%, white solid, m.p. 154–158 °C. ^1^H NMR (400 MHz, CDCl_3_): *δ* 1.47 (9H, s, *t*-Bu), 1.53 (9H, s, *t*-Bu), 4.10 (2H, d, *J* = 6.0 Hz, CH_2_), 4.90 (1H, br.s, NH), 7.17 (2H, d, *J* = 8.0 Hz, 2 × ArH), 7.46 (2H, d, *J* = 8.4 Hz, 2 × ArH), 7.49–7.55 (2H, m, 2 × ArH), 7.56–7.62 (1H, m, ArH), 7.87 (1H, d, *J* = 1.6 Hz, ArH), 7.89 (1H, s, ArH), 10.51 (1H, br.s, NH), and 11.65 (1H, br.s, NH). HRMS (ESI): Calcd. for C_24_H_33_N_4_O_6_S (M + H)^+^ 505.2115 and found 505.2127.

**Amino((4-(benzamidomethyl)phenyl)amino)methaniminium 2,2,2-trifluoroacet-ate (48a):** Compound **47a** (0.1 mmol) was dissolved in CH_2_Cl_2_ (0.8 mL) and TFA (0.2 mL) was added, and the reaction mixture was stirred for 2 h at 0 °C. The mixture was concentrated to dryness to give **48a** as a pale yellow glass (38 mg, 99%). The product was a mixture of conformers as revealed by ^1^H NMR. Major conformer: ^1^H NMR (400 MHz, DMSO-*d_6_*): *δ* 4.54 (2H, s, CH_2_), 7.25 (2H, d, *J* = 8.0 Hz, 2 × ArH), 7.44 (4H, br.s, 2 × NH_2_), 7.49–7.67 (5H, m, 5 × ArH), 7.94 (2H, d, *J* = 7.2 Hz, 2 × ArH), 9.17 (1H, t, *J* = 5.8 Hz, NH), and 9.79 (1H, s, NH). HRMS (ESI): Calcd. for C_15_H_17_N_4_O (M + H)^+^ 269.1397 and found 269.1405.

**Amino((4-(phenylsulfonamidomethyl)phenyl)amino)methaniminium 2,2,2-trifluoroacetate (48b):** Compound **47b** (0.1 mmol) was dissolved in CH_2_Cl_2_ (0.8 mL) and TFA (0.2 mL) was added, and the reaction mixture was stirred for 2 h at 0 °C. The mixture was concentrated to dryness to give **48b** as a pale yellow glass (38 mg, 99%). ^1^H NMR (400 MHz, DMSO-*d_6_*): *δ* 4.03 (2H, d, *J* = 6.4 Hz, CH_2_), 7.21 (2H, d, *J* = 8.0 Hz, 2 × ArH), 7.37 (2H, d, *J* = 8.0 Hz, 2 × ArH), 7.45 (4H, br.s, 2 × NH_2_), 7.61–7.72 (3H, m, 3 × ArH), 7.87 (2H, d, *J* = 7.2 Hz, 2 × ArH), 8.27 (1H, t, *J* = 6.4 Hz, NH), and 9.78 (1H, br.s, NH). HRMS (ESI): Calcd. for C_14_H_17_N_4_O_2_S (M + H)^+^ 305.1067 and found 305.1078.

**(3-[2,3-Dichlorobenzyloxy]benzyl)(methyl)amine (49a):** In a solution of **38a** (330 mg, 1.17 mmol) in MeOH (5 mL), MeNH_2_ (2.0 M in MeOH, 0.76 mL, 1.5 mmol) was added. The mixture was stirred at r.t. for 10 h and then cooled to 0 °C. NaBH_4_ (53 mg, 1.4 mmol) was added slowly. The mixture was stirred at r.t. for 4 h and then partitioned between NaOH (1M in water) and EtOAc. The organic layer was washed with brine, dried (MgSO_4_), and concentrated in vacuo to give **49a** as a clear oil (340 mg, 98%). ^1^H NMR (400 MHz, CDCl_3_): *δ* 2.45 (3H, s, NCH_3_), 3.74 (2H, s, CH_2_), 5.15 (2H, s, OCH_2_), 6.85 (1H, dd, *J* = 8.0, 1.5 Hz, ArH), 6.92–7.00 (2H, m, 2 × ArH), 7.18–7.28 (2H, m, 2 × ArH), 7.40 (1H, d, *J* = 7.9 Hz, ArH), and 7.47 (1H, d, *J* = 7.9 Hz, ArH). HRMS (ESI): Calcd. for C_15_H_16_Cl_2_NO (M + H)^+^ 296.0609 and found 296.0680.

**(3-[2,3-Dichlorobenzyloxy]benzyl)(2-methoxyethyl)amine (49b):** In a solution of **38a** (330 mg, 1.17 mmol) in MeOH (5 mL), 2-methoxyethan-1-amine (113 mg, 1.5 mmol) was added. The mixture was stirred at r.t. for 10 h and then cooled to 0°C. NaBH_4_ (57 mg, 1.5 mmol) was added slowly. The mixture was stirred at r.t. for 4 h and then partitioned between NaOH (1M in water) and EtOAc. The organic layer was washed with brine, dried (MgSO_4_), and concentrated in vacuo to give **49b** as a clear oil (350 mg, 88%). ^1^H NMR (400 MHz, CDCl_3_): *δ* 2.80 (2H, t, *J* = 5.2 Hz, OCH_2_C*H*_2_N), 3.35 (3H, s, OCH_3_), 3.51 (2H, t, *J* = 5.3 Hz, OC*H*_2_CH_2_N), 3.80 (2H, s, CH_2_), 5.16 (2H, s, OCH_2_), 6.85 (1H, dd, *J* = 7.9, 2.6 Hz, ArH), 6.96 (1H, d, *J* = 7.5 Hz, ArH), 6.99 (1H, s, ArH), 7.20–7.26 (2H, m, 2 × ArH), 7.43 (1H, d, *J* = 8.0 Hz, ArH), and 7.48 (1H, d, *J* = 7.9 Hz, ArH). HRMS (ESI): Calcd. for C_17_H_20_Cl_2_NO_2_ (M + H)^+^ 340.0871 and found 340.0848.

***tert*-Butyl *N*-[{[(*tert*-butoxy)carbonyl]imino}[(3-(2,3-dichlorobenzyl) benzyl)(methyl)amino]methyl]carbamate (50a):** In a solution of **49a** (330 mg, 1.11 mmol) in DMF (5 mL), *S*-methyl-*N*,*N*’-bis(*tert*-butoxycarbonyl)isothiourea (389 mg, 1.3 mmol) was added, followed by Et_3_N (0.3 mL) and HgCl_2_ (200 mg). The mixture was stirred at r.t. overnight, and then diluted with EtOAc (50 mL) and filtered through Celite. The organic layer was washed with brine, dried (MgSO_4_), and concentrated in vacuo to give a colourless oil. Purification by flash column chromatography (petrol ether to petrol ether/EtOAc 4:1) afforded **50a** as a white solid (350 mg, 59% yield), m.p. 127–129 °C. ^1^H NMR (400 MHz, CDCl_3_): *δ* 1.50 (18H, s, 2 × *t*-Bu), 2.90 (3H, s, NCH_3_), 4.70 (2H, s, CH_2_), 5.16 (2H, s, OCH_2_), 6.88–6.97 (2H, m, 2 × ArH), 7.22–7.28 (3H, m, 3 × ArH), 7.41 (1H, d, *J* = 7.9 Hz, ArH), 7.49 (1H, d, *J* = 7.9 Hz, ArH), and 10.2 (1H, s, NH). HRMS (ESI): Calcd. for C_26_H_33_Cl_2_N_3_NaO_5_ (M + Na)^+^ 560.1695 and found 560.1697.

***tert*-Butyl *N*-[{[(*tert*-butoxy)carbonyl]imino}[(3-(2,3-dichlorobenzyl)benzyl)(2-methoxyethyl)amino]methyl]carbamate (50b):** In a solution of **49b** (320 mg, 0.94 mmol) in DMF (3 mL), *S*-methyl-*N*,*N*’-bis(*tert*-butoxycarbonyl)isothiourea (330 mg, 1.13 mmol) was added, followed by Et_3_N (0.5 mL) and HgCl_2_ (306 mg). The mixture was stirred at r.t. overnight, and then diluted with EtOAc (50 mL) and filtered through Celite. The organic layer was washed with brine, dried (MgSO_4_), and concentrated in vacuo to give a colourless oil. Purification by flash column chromatography eluting with a gradient solvent (petrol ether to petrol ether/EtOAc 4:1) afforded **50b** as a white solid (340 mg, 62% yield), m.p. 125–126 °C. ^1^H NMR (400 MHz, CDCl_3_): *δ* 1.50 (18H, s, 2 × *t*-Bu), 3.30 (3H, s, OCH_3_), 3.45 (2H, s, CH_2_), 3.65 (2H, br.s, OCH_2_C*H*_2_N), 4.89 (2H, br.s, OC*H*_2_CH_2_N), 5.20 (2H, s, OCH_2_), 6.86–6.97 (2H, m, 2 × ArH), 7.21–7.28 (3H, m, 3 × ArH), 7.43 (1H, d, *J* = 8.3 Hz, ArH), 7.49 (1H, d, *J* = 8.2 Hz, ArH), and 9.56 (1H, s, NH). HRMS (ESI): Calcd. for C_28_H_38_Cl_2_N_3_O_6_ (M + H)^+^ 582.2138 and found 582.2177.

**1-(3-(2,3-Dichlorobenzyloxy)benzyl)-1-methylguanidinium chloride (51a):** In a solution of **50a** (126 mg, 0.23 mmol) in CH_2_Cl_2_ (1.5 mL), TFA (0.75 mL) was added. The mixture was shaken at r.t. overnight and then evaporated to dryness. Et_2_O (1 mL) was added, and the precipitate was collected, washed with Et_2_O, and dried in vacuo to give a white solid (69 mg, 87%). The solid (18 mg) was dissolved in HCl (0.5M in MeOH, 5 mL), and concentrated in vacuo to give **51a** as a white solid (15 mg). ^1^H NMR (400 MHz, CDCl_3_): *δ* 2.96 (3H, s, NCH_3_), 4.56 (2H, br.s, CH_2_), 5.18 (2H, s, OCH_2_), 6.76–6.86 (2H, m, 2 × ArH), 6.98 (1H, d, *J* = 8.2 Hz, ArH), 7.18–7.36 (2H, m, 2 × ArH), and 7.49 (2H, br.s, 2 × ArH). HRMS (ESI): Calcd. for C_16_H_18_Cl_2_N_3_O (M + H)^+^ 338.0827 and found 338.0909.

**1-(3-(2,3-Dichlorobenzyl)benzyl)-1-(2-methoxyethyl)guanidinium 2,2,2-trifluoroacetate (51b):** In a solution of the intermediate **50b** (98 mg, 0.17 mmol) in CH_2_Cl_2_ (1.5 mL), TFA (0.75 mL) was added. The mixture was shaken at r.t. overnight and then evaporated to dryness. Et_2_O (1 mL) was added, and the precipitate was collected, washed with Et_2_O, and dried in vacuo to give **51b** as a white solid (70 mg, 86%). ^1^H NMR (400 MHz, CDCl_3_): *δ* 3.31 (3H, s, NCH_3_), 3.56 (4H, m, OCH_2_C*H*_2_N and CH_2_), 4.67 (2H, br.s, OC*H*_2_CH_2_N), 5.26 (2H, s, OCH_2_), 6.85–6.95 (2H, m, 2 × ArH), 7.06 (1H, dd, *J* = 7.9, 2.1 Hz, ArH), 7.37–7.50 (2H, m, 2 × ArH), 7.62 (1H, dd, *J* = 8.1, 1.8 Hz, ArH), and 7.73 (1H, dd, *J* = 8.1, 1.7 Hz, ArH). HRMS (ESI): Calcd. for C_18_H_22_Cl_2_N_3_O_2_ (M + H)^+^ 382.1089 and found 382.1195.

**3-(2,3-Dichlorobenzyloxy)phenylmethanol (52):** Compound **38a** (1.3 g, 4.6 mmol) was dissolved in EtOH (20 mL) and THF (5 mL) and cooled to 0 °C. NaNH_4_ (176 mg, 4.6 mmol) was added portionwise and the mixture was stirred at r.t. overnight. Acetone (2 mL) was added, and the mixture was partitioned between EtOAc and a saturated NH_4_Cl solution. The organic layer was washed with brine, dried (MgSO_4_), and concentrated in vacuo to give **52** as a white solid (1.2 g, 92% yield), m.p. 79–80 °C. ^1^H NMR (400 MHz, CDCl_3_): *δ* 4.64 (2H, s, OCH_2_), 5.24 (2H, s, OCH_2_), 6.90 (1H, dd, *J* = 8.0, 1.9 Hz, ArH), 6.96–7.03 (2H, m, 2 × ArH), 7.20–7.31 (2H, m, 2 × ArH), 7.43 (1H, d, *J* = 8.2 Hz, ArH), and 7.49 (1H, d, *J* = 8.2 Hz, ArH).

**1,2-Dichloro-3-[3-(chloromethyl)phenoxymethyl]benzene (53):** In a solution of **52** (1.1 g, 3.9 mmol) in CH_2_Cl_2_ (20 mL), Et_3_N (1.1 mL, 7.8 mmol) was added, followed by methanesulfonyl chloride (0.6 mL, 7.8 mmol). The mixture was stirred at r.t. overnight and partitioned between CH_2_Cl_2_ and a saturated NaHCO_3_ solution. The organic layer was washed with brine, dried (MgSO_4_), and concentrated in vacuo to give **53** as a white solid (0.95 g, 91% yield), m.p. 91–95 °C. ^1^H NMR (400 MHz, CDCl_3_): *δ* 4.53 (2H, s, CH_2_), 5.17 (2H, s, OCH_2_), 6.92 (1H, dd, *J* = 8.1, 1.7 Hz, ArH), 7.00–7.05 (2H, m, 2 × ArH), 7.21–7.32 (2H, m, 2 × ArH), 7.44 (1H, d, *J* = 8.1 Hz, ArH), and 7.49 (1H, d, *J* = 8.1 Hz, ArH).

***tert*-Butyl *N*-[{[(*tert*-butoxy)carbonyl](3-(2,3-dichlorobenzyloxy) benzyl)amino}-(methylsulfanyl)methylidene]carbamate (54):** In a solution of **53** (330 mg, 1.1 mmol) in CH_2_Cl_2_ (5 mL), KOH (112 mg, 2.2 mmol) in water (5 mL) and Bu_4_NHSO_4_ (34 mg, 0.1 mmol) were added. The mixture was stirred at r.t. overnight and partitioned between CH_2_Cl_2_ and water. The organic layer was washed with brine, dried (MgSO_4_), and concentrated in vacuo to give **54** as a clear oil (300 mg, 49% yield). ^1^H NMR (400 MHz, CDCl_3_): *δ* 1.39 (9H, s, *t*-Bu), 1.51 (9H, s, *t*-Bu), 2.40 (3H, s, SCH_3_), 4.75 (2H, s, CH_2_), 5.15 (2H, s, OCH_2_), 6.86 (1H, dd, *J* = 7.9, 1.7 Hz, ArH), 6.95–7.01 (2H, m, 2 × ArH), 7.18–7.26 (2H, m, 2 × ArH), 7.38 (1H, d, *J* = 8.2 Hz, ArH), and 7.47 (1H, d, *J* = 8.1 Hz, ArH). HRMS (ESI): Calcd. for C_26_H_32_Cl_2_N_2_NaO_5_S (M + Na)^+^ 577.1306 and found 577.1303.

***tert*-Butyl *N*-[{[(*tert*-butoxy)carbonyl](3-(2,3-dichlorobenzyl) benzyl)amino}(methylamino)methylidene]carbamate (55):** In a solution of **54** (200 mg, 0.36 mmol) in DMF (1 mL), MeNH_2_ (2M in MeOH, 0.27 mL, 0.54 mmol) was added, followed by Et_3_N (0.2 mL) and HgCl_2_ (116 mg). The mixture was stirred at r.t. for 1.5 h, and then diluted with EtOAc (30 mL) and filtered through Celite. The organic layer was washed with brine, dried (MgSO_4_), and concentrated in vacuo to give a colourless oil. Purification by flash column chromatography (petrol ether to petrol ether/EtOAc 4:1) afforded **55** as a white solid (100 mg, 52% yield), m.p. 111–113 °C. ^1^H NMR (400 MHz, CDCl_3_): *δ* 1.46 (9H, s, *t*-Bu), 1.51 (9H, s, *t*-Bu), 2.52 (3H, s, NCH_3_), 4.57 (2H, s, CH_2_), 5.15 (2H, s, OCH_2_), 6.87–6.96 (3H, m, 3 × ArH), 7.19–7.26 (2H, m, 2 × ArH), 7.43 (1H, d, *J* = 7.9 Hz, ArH), 7.49 (1H, d, *J* = 8.0 Hz, ArH), and 9.87 (1H, s, NH).

**3-(3-(2,3-Dichlorobenzyloxy)benzyl)-1-methylguanidinium 2,2,2-trifluoroacetate (56):** In a solution of **55** (85 mg, 0.16 mmol) in CH_2_Cl_2_ (1 mL), TFA (0.5 mL) was added. The mixture was shaken at r.t. overnight and then evaporated to dryness. The residue was dissolved in MeOH and evaporated to dryness to give **56** as a foamy powder (60 mg, 87%). ^1^H NMR (400 MHz, CDCl_3_): *δ* 2.85 (3H, s, NCH_3_), 4.39 (2H, s, CH_2_), 5.19 (2H, s, OCH_2_), 6.88–6.95 (3H, m, 3 × ArH), 7.24–7.33 (2H, m, 2 × ArH), and 7.52 (2H, d, *J* = 7.6 Hz, 2 × ArH). HRMS (ESI): Calcd. for C_16_H_18_Cl_2_N_3_O (M + H)^+^ 338.0827 and found 338.0945.

### 3.2. Biology

#### 3.2.1. Materials

*Staphylococcus aureus* 9518 and *Escherichia coli* K12 were purchased from the National Collections of Industrial and Marine Bacteria (NCIMB), Aberdeen, UK. MRSA strains were a gift from Dr Llinos Harris, Swansea University.

#### 3.2.2. Methods

##### Cell Culture

The bacteria were grown for 17 h in 20 mL of tryptic soy broth (TSB) at 30 °C with oscillation (90 oscillations per minute). A total of 1 mL of the overnight culture was taken and centrifuged at 880 g for 7 min. The supernatant was removed, and the pellet resuspended in PBS. The centrifugation step was then repeated, and the resulting pellet redissolved in an appropriate volume to adjust the concentration of the bacterial solution to a density of 1 × 10^5^ cells/mL based on the haemocytometer calculation in a 30 mL universal test tube.

##### Treatment Protocol

The synthetic compounds were dissolved initially at a concentration of 1 mg/mL in a suitable solvent (ethanol, methanol, or DMSO) before dilution with water in order to generate the relevant concentrations used within the bioassay. Each solvent was tested separately for its own toxicity, and it was ensured that the dilution required to produce the working solutions for the assay was sufficient to remove any toxic effect of the initial solvent used. The bacterial solution (10 µL) was added to 50 µL of either the synthetic compound solution or water as a negative control in a 96-well plate. This was sealed with a polyethylene seal prior to being analysed in a Skanit platereader (Thermoscientific, Cambridge, UK). The optical density at 550 nm was then recorded once every hour for 24 h, with a shaking step immediately prior to each reading being taken.

##### Quantification Method

The acquired data were then used to determine the growth of each species with or without the addition of each synthetic compound solution. The MIC values were determined as the minimum concentration of compound required to reduce the survival index to less than 50%. The survival indices (Table 7) were determined from the absorbance data as the ratio of the optical density of the control bacteria at the mid-log point of growth to the comparative optical density of the treatment bacteria multiplied by 100, as published previously [36].

## 4. Conclusions

A range of benzyl, phenyl guanidine, and aminoguanidine hydrazone derivatives were synthesised and evaluated. Some derivatives displayed excellent antimicrobial activity. The best benzyl guanidine derivatives **9g**, **9m**, and **9v** displayed antimicrobial MICs of 0.5–1.0 µg/mL against *S. aureus* and 1–4 µg/mL against *E. coli*. The best aminoguanidine hydrazone derivative **10d** showed very good potency against *S. aureus* (MIC 1 µg/mL) but was significantly less potent against *E. coli* (16 µg/mL), as indeed was benzyl guanidine **9p** (0.5 µg/mL and 16 µg/mL, respectively). Overall, **10a**, **10j**, and **10r–s** were less potent against *S. aureus* (MIC of 4 µg/mL) and more potent against *E. coli* (MIC of 4 µg/mL) compared to **10d**. The most potent benzyl guanidines **9m** and **9v** were tested against methicillin-resistant *Staphylococcus aureus* (MRSA 3 and MRSA 15) and showed very promising potencies with MICs of 0.5–2.0 µg/mL and low survival indices (1.76–12.76%). Further work is currently underway to establish the mechanism of action of these new guanidine derivatives, and it seems clear, as expected, that the guanidino/amidino functionality is a key contributor to the antibacterial activity. However, a preliminary further evaluation of a selection of the most potent compounds shows potentially complex profiles that may not include the most expected bacterial cell division machinery, such as FtsZ, as a direct target. Since it is known that the lead agent TXA497 **8** targets both the bacterial membrane in addition to FtsZ, with some cells exhibiting the effects of membrane disruption [31], the focus of future studies on the most potent subset of optimised compounds will address both FtsZ and the bacterial cell membrane, and also potential targets elsewhere.

## Figures and Tables

**Table 1:** Antibacterial activities of benzyl guanidine derivatives **9a–v**, **51a–b**, and **56** against *S. aureus* and *E. coli*.

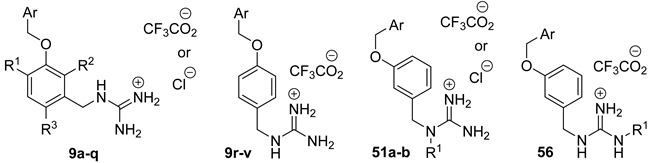						
Cpd	Ar	R^1^	R^2^	R^3^	MIC/µg/mL	
					*S. aureus*	*E. coli*
**9a**	C_6_H_5_	H	H	H	8	128
**9b**	4-Cl-C_6_H_4_	H	H	H	8	8
**9c**	3-Cl-C_6_H_4_	H	H	H	16	32
**9d**	2,4-di-Cl-C_6_H_3_	H	H	H	>256	8
**9e**	3,4-di-Cl-C_6_H_3_	H	H	H	4	16
**9f**	2,5-di-Cl-C_6_H_3_	H	H	H	2	16
9g *	2,3-di-Cl-C_6_H_3_	H	H	H	1	1
**9h**	4-CF_3_-C_6_H_4_	H	H	H	8	4
**9i**	3-CF_3_-C_6_H_4_	H	H	H	1	8
**9j**	4-Br-C_6_H_4_	H	H	H	4	32
**9k**	4-F-C_6_H_4_	H	H	H	64	32
9m *	2-Cl-3-CF_3_-C_6_H_3_	H	H	H	0.5	1
**9n**	3-Cl-C_6_H_4_	MeO	H	H	128	128
**9o**	4-Cl-C_6_H_4_	MeO	H	H	8	64
**9p**	2,3-di-Cl-C_6_H_3_	H	F	F	0.5	16
**9q**	3-CF_3_-C_6_H_4_	H	F	F	1	8
**9r**	C_6_H_5_	--	--	--	16	128
**9s**	4-Cl-C_6_H_4_	--	--	--	32	32
**9t**	3-Cl-C_6_H_4_	--	--	--	16	16
**9u**	3,4-di-Cl-C_6_H_3_	--	--	--	2	128
**9v**	2,3-di-Cl-C_6_H_3_	--	--	--	0.5	4
51a*	2,3-di-Cl-C_6_H_3_	Me	--	--	32	32
**51b**	2,3-di-Cl-C_6_H_3_	MeOCH_2_CH_2_	--	--	32	32
**56**	2,3-di-Cl-C_6_H_3_	Me	--	--	16	32

* Chloride salt.

**Table 2:** Antibacterial activities of benzyl guanidine derivatives **20a–e** and **24a–e** against *S. aureus* and *E. coli*.

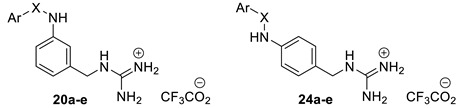				
Cpd	Ar	X	MIC/µg/mL	
			*S. aureus*	*E. coli*
**20a**	3-Cl-C_6_H_4_	SO_2_	>32	>32
**20b**	4-Cl-C_6_H_4_	SO_2_	>32	>32
**20c**	2,3-di-Cl-C_6_H_3_	SO_2_	>32	>32
**20d**	3-CF_3_-C_6_H_4_	SO_2_	>32	>32
**20e**	C_6_H_5_	CO	>32	>32
**24a**	3-Cl-C_6_H_4_	SO_2_	>32	>32
**24b**	4-Cl-C_6_H_4_	SO_2_	>32	>32
**24c**	2,3-di-Cl-C_6_H_3_	SO_2_	>32	>32
**24d**	3-CF_3_-C_6_H_4_	SO_2_	>32	>32
**24e**	C_6_H_5_	CO	>32	>32

**Table 3:** Antibacterial activities of tetrahydroisoquinoline derivatives **29a–b** and **33** against *S. aureus* and *E. coli*.

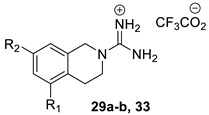				
Cpd	R^1^	R^2^	MIC/µg/mL	
			*S. aureus*	*E. coli*
**29a**	H	2,3-di-Cl-C_6_H_3_CH_2_O	16	32
**29b**	2,3-di-Cl-C_6_H_3_CH_2_O	H	8	8
**33**	H	4-*t*-Bu-C_6_H_4_	64	>128

**Table 4:** Antibacterial activities of benzyloxy guanidine derivatives **36a–c** against *S. aureus* and *E. coli*.

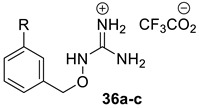			
Cpd	R	MIC/µg/mL	
		*S. aureus*	*E. coli*
**36a**	C_6_H_5_	128	>128
**36b**	C_6_H_5_O	>128	>128
**36c**	4-*t*-Bu-C_6_H_4_	>64	>64

**Table 5:** Antibacterial activities of aminoguanidine hydrazone derivatives **10a–t** and **41a–h** against *S. aureus* and *E. coli*.

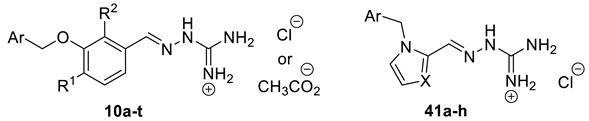						
Cpd	Ar	R^1^	R^2^	X	MIC/µg/mL	
					*S. aureus*	*E. coli*
10a *	2,3-di-Cl-C_6_H_3_	H	H	--	4	4
10b *	2-Cl-3-CF_3_-C_6_H_3_	H	H	--	4	8
**10c**	4-Cl-C_6_H_4_	H	H	--	16	8
**10d**	4-CF_3_-C_6_H_4_	H	H	--	1	16
**10e**	3-Cl-C_6_H_4_	H	H	--	8	16
**10f**	3-CF_3_-C_6_H_4_	H	H	--	32	8
10g *	2,3-di-Cl-C_6_H_3_	MeO	H	--	16	8
**10h**	2-Cl-3-MeO-C_6_H_3_	H	Cl	--	32	32
**10i**	C_6_H_5_	H	Cl	--	8	8
**10j**	4-Cl-C_6_H_4_	H	Cl	--	4	4
**10k**	2,3-di-Cl-C_6_H_3_	H	Cl	--	8	32
**10m**	2-Cl-3-CF_3_-C_6_H_3_	H	Cl	--	8	32
**10n**	2,4-di-Cl-C_6_H_3_	H	Cl	--	8	32
**10o**	2,5-di-Cl-C_6_H_3_	H	Cl	--	32	32
**10p**	3,4-di-Cl-C_6_H_3_	H	Cl	--	8	8
**10q**	2,3,5-tri-Cl-C_6_H_2_	H	Cl	--	32	32
**10r**	3-CF_3_-C_6_H_4_	H	Cl	--	4	4
**10s**	4-CF_3_-C_6_H_4_	H	Cl	--	4	4
**10t**	3-Cl-C_6_H_4_	H	Cl	--	16	8
**41a**	3-Cl-C_6_H_4_	--	--	N	16	32
**41b**	4-Cl-C_6_H_4_	--	--	N	16	16
**41c**	3-CF_3_-C_6_H_4_	--	--	N	16	32
**41d**	4-CF_3_-C_6_H_4_	--	--	N	8	16
**41e**	3-Cl-C_6_H_4_	--	--	CH	16	32
**41f**	4-Cl-C_6_H_4_	--	--	CH	16	32
**41g**	3-CF_3_-C_6_H_4_	--	--	CH	16	16
**41h**	4-CF_3_-C_6_H_4_	--	--	CH	16	16

* Acetate salt.

**Table 6:** Antibacterial activities of phenyl guanidine derivatives **45a–c** and **48a–b** against *S. aureus* and *E. coli*.

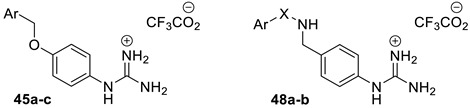				
Cpd	Ar	X	MIC/µg/mL		
			*S. aureus*	*E. coli*
**45a**	C_6_H_5_	--	8	>128
**45b**	4-Cl-C_6_H_4_	--	128	128
**45c**	3-Cl-C_6_H_4_	--	16	64
**48a**	C_6_H_5_	CO	>32	>32
**48b**	C_6_H_5_	SO_2_	>32	>32

**Table 7:** Antibacterial activities and survival indices of **9m** and **9v** against MRSA 3 and MRSA 15.

Compound	MIC/µg/mL	Survival Index/%
**9m** vs. MRSA 3	0.5	12.76
**9m** vs. MRSA 15	1	1.90
**9v** vs. MRSA 3	1	1.76
**9v** vs. MRSA 15	2	8.48

## Data Availability

All relevant data are available in the article.

## Sample Availability

Samples of some compounds may be available from the authors upon request.
